# Transcriptomic and Functional Landscape of Adult Human Spinal Cord NSPCs Compared to iPSC-Derived Neural Progenitor Cells

**DOI:** 10.3390/cells14020064

**Published:** 2025-01-07

**Authors:** Sasi Kumar Jagadeesan, Ahmad Galuta, Ryan Vimukthi Sandarage, Eve Chung Tsai

**Affiliations:** 1 Department of Neurosciences, Faculty of Medicine, University of Ottawa, Ottawa, ON K1H 8M5, Canada; sjagadeesan@ohri.ca (S.K.J.); agaluta083@uottawa.ca (A.G.); 2 Neuroscience Program, Ottawa Hospital Research Institute, The Ottawa Hospital, Ottawa, ON K1Y 4E9, Canada; 3 Division of Neurosurgery, Department of Surgery, The Ottawa Hospital, Ottawa, ON K1H 8L6, Canada; rsandarage@toh.ca

**Keywords:** spinal cord injury, neural stem/progenitor cells, induced pluripotent stem cells, transcriptomics, neurogenesis, differentiation potential, regenerative medicine, cellular heterogeneity, proliferation dynamics, patient-specific variability

## Abstract

The adult human spinal cord harbors diverse populations of neural stem/progenitor cells (NSPCs) essential for neuroregeneration and central nervous system repair. While induced pluripotent stem cell (iPSC)-derived NSPCs offer significant therapeutic potential, understanding their molecular and functional alignment with bona fide spinal cord NSPCs is crucial for developing autologous cell therapies that enhance spinal cord regeneration and minimize immune rejection. In this study, we present the first direct transcriptomic and functional comparison of syngeneic adult human NSPC populations, including bona fide spinal cord NSPCs and iPSC-derived NSPCs regionalized to the spinal cord (iPSC-SC) and forebrain (iPSC-Br). RNA sequencing analysis revealed distinct transcriptomic profiles and functional disparities among NSPC types. iPSC-Br NSPCs exhibited a close resemblance to bona fide spinal cord NSPCs, characterized by enriched expression of neurogenesis, axon guidance, synaptic signaling, and voltage-gated calcium channel activity pathways. Conversely, iPSC-SC NSPCs displayed significant heterogeneity, suboptimal regional specification, and elevated expression of neural crest and immune response-associated genes. Functional assays corroborated the transcriptomic findings, demonstrating superior neurogenic potential in iPSC-Br NSPCs. Additionally, we assessed donor-specific influences on NSPC behavior by analyzing gene expression and differentiation outcomes across syngeneic populations from multiple individuals. Donor-specific factors significantly modulated transcriptomic profiles, with notable variability in the alignment of iPSC-derived NSPCs to bona fide spinal cord NSPCs. Enrichment of pathways related to neurogenesis, axon guidance, and synaptic signaling varied across donors, highlighting the impact of genetic and epigenetic individuality on NSPC behavior.

## 1. Introduction

Spinal cord injuries (SCIs) are devastating conditions characterized by disrupted motor and sensory functions, often resulting in permanent disability [1,2]. Recovery outcomes depend on the severity and location of the injury, with limited regeneration observed in the adult spinal cord, especially in severe cases [3]. Key barriers to recovery include the inability to replace lost neurons essential for signal transmission, remyelinate axons for efficient conduction, and restore functional synaptic connections necessary for communication between disrupted neural circuits [1,4,5]. These deficits highlight the urgent need for regenerative strategies capable of promoting effective repair and functional recovery in SCI patients.

Neural stem/progenitor cells (NSPCs) are pivotal to spinal cord repair due to their intrinsic ability to self-renew and differentiate into neurons, astrocytes, and oligodendrocytes, the primary cell types required for central nervous system (CNS) regeneration [6,7,8,9]. Bona fide NSPCs, isolated directly from the adult human spinal cord, represent an endogenous source for repair. However, their limited availability and tightly regulated behavior in vivo pose significant challenges for therapeutic applications [8,10,11]. Induced pluripotent stem cells (iPSCs) offer a scalable, syngeneic alternative, enabling the generation of patient-specific NSPCs for autologous therapies that minimize immune rejection and align with the unique needs of each individual [12,13]. Advances in iPSC differentiation protocols have enabled the generation of regionally specified NSPCs, including spinal cord-like (iPSC-SC) and forebrain-like (iPSC-Br) NSPCs, by mimicking embryonic developmental cues through the modulation of the Wnt, SHH, and FGF signaling pathways [14,15,16]. While preclinical studies have demonstrated the potential of iPSC-derived NSPCs in promoting neuroregeneration, their ability to accurately replicate the transcriptomic and functional profiles of bona fide adult spinal cord NSPCs remains insufficiently explored [12,17]. Most studies to date have relied on animal models or non-syngeneic comparisons, leaving critical gaps in their translational relevance to human SCI repair [2,4,17]. Bona fide spinal cord NSPCs serve as a critical benchmark for evaluating iPSC-derived NSPCs due to their natural adaptation to the spinal cord microenvironment and tightly regulated proliferation and differentiation essential for CNS function [18,19]. However, challenges persist with iPSC-derived NSPCs, including variability in differentiation outcomes and residual traits linked to pluripotency [20]. iPSC-SC NSPCs, generated through neuromesodermal progenitor intermediates, often exhibit heterogeneity and reduced neurogenic potential [21]. In contrast, iPSC-Br NSPCs, patterned using dual SMAD inhibition, have shown greater transcriptomic similarity to bona fide spinal cord NSPCs and enhanced neurogenic potential [22]. Despite these findings, systematic comparisons of transcriptomic and functional properties between these NSPC types in human tissues remain lacking [23,24].

To address these challenges, we conducted a syngeneic comparison of bona fide spinal cord NSPCs, iPSC-SC NSPCs, and iPSC-Br NSPCs derived from adult human donors. This approach minimizes inter-donor variability, enabling a direct evaluation of NSPC behavior within a unified genetic and epigenetic background populations. Our transcriptomic analyses revealed that iPSC-Br NSPCs align closely with bona fide spinal cord NSPCs, particularly in pathways linked to neurogenesis, axon guidance, and synaptic function. Conversely, iPSC-SC NSPCs displayed markers of immature regional specification, including elevated expression of neural crest and immune-response-associated genes. Functional assays demonstrated robust neurogenic potential in iPSC-Br NSPCs, reflected in their ability to differentiate into β-III tubulin+ neurons and GFAP+ astrocytes, compared to the limited differentiation capacity of iPSC-SC NSPCs. Donor-specific factors, including genetic variability influencing differential gene expression and epigenetic modifications such as DNA methylation and histone alterations, emerged as critical modulators of NSPC behavior, shaping their proliferation, differentiation potential, and alignment with bona fide spinal cord NSPCs.

## 2. Methods

### 2.1. Ethics Statement

Ethical approval for the extraction of human spinal cord tissue was granted by the Ottawa Health Science Network Research Ethics Board. The consent process and documentation adhered to the template provided by the University of Ottawa and were in compliance with the Tri-Council Policy Statement Guidelines (Canadian Institutes of Health Research, 2022) [25,26,27]. Informed written consent was obtained from the next of kin of the deceased organ donor [28].

### 2.2. Spinal Cord Harvest and Primary NSPC Culture

Human spinal cords were harvested using an anterior approach as previously described [27]. Briefly, after the organ harvest exposure was completed, an osteotome was used to make angled axial cuts to partially resect the L2 vertebral body and expose the dura. Using a sternal saw or osteotomes, the anterior vertebral bodies were removed to expose the dura cranially to the limit of the organ harvest exposure. The dura was transversely incised with scissors. The dura was retracted to expose the nerve root sleeves bilaterally and the nerve roots and their dural sleeves were transected with scissors bilaterally to the rostral extent of the organ harvest exposure. The most rostal extent of the spinal cord and thecal sac were then transversely incised with scissors to resect the spinal cord specimen. The dura was then incised to expose the spinal cord, which was then divided with scalpel. The harvested spinal cord segments were then placed in cold, sterile dissection media, consisting of 1X Hank’ Balanced Salt Solution (HBSS) + 0.6% D-glucose + 2% penicillin–streptomycin (PS) (Gibco, 15140122). The spinal cord tissue was carefully processed for micro-dissection under a dissection microscope to isolate the ependymal cell-rich region of the central canal. This novel technique, as opposed to prior methods significantly reduces contamination from surrounding gray and white matter [27]. The spinal cord was first cleaned of its meninges, sectioned into 1–2 mm slices with a scalpel, and then micro-dissected to extract a cuboidal sample containing the central canal. The excised samples were then dissociated using the papain dissociation kit Worthington Biochemical Inc. (Lakewood, NJ, USA, LK003150) according to the manufacturer’s instructions. After purification, the cells were resuspended in a serum-free medium (SFM) containing: Neurobasal-A medium (ThermoFisher Scientific, Waltham, MA, USA, 10888022), 2 mM L-glutamine (Gibco, Waltham, MA, USA, A2916801), 100 U/mL PS, 1% B27-vitamin A (Gibco, Waltham, MA, USA, 12587010), and 10% hormone mix (1:1 DMEM/F12 (Sigma, St. Louis, MO, USA, D8900), 0.6% glucose, 3 mM NaHCO_3_, 5 mM HEPES, 25 µg/mL insulin, 100 µg/mL apo-transferrin, 10 µM putrescine, 30 nM selenium, and 20 nM progesterone in distilled water). The cells were seeded in Matrigel-coated six-well plates (Corning, Corning, NY, USA) at 20 cells/µL in 4 mL of proliferation media (1X EFH):— SFM, 20 ng/mL human epidermal growth factor (EGF) (Sigma, St. Louis, MO, USA, E9644), 20 ng/mL human basic fibroblast growth factor (FGF2) (Sigma, St. Louis, MO, USA, F3685), and 2 µg/mL heparin (Sigma, St. Louis, MO, USA, H3149)—and incubated undisturbed for one week at 37°C, 5% CO_2_, and 20% O_2_. The cultures were maintained by replacing half of the medium with 2X EFH every 2–3 days for 1–2 weeks. Once the cultures were visibly expanding, the entire media were replaced with 2 mL of 1X EFH until the cultures reached confluence. At this point, NSPCs were passaged and assessed. 

### 2.3. Skin Harvest and Primary Fibroblast Culture

Human skin tissue samples were harvested under sterile conditions in the operating room. A full-thickness elliptical sample (2 cm × 2 cm) was excised from the periumbilical region, placed in cold, sterile HBSS on ice, and transported to the lab. In a biohood, subcutaneous fat was removed from the tissue using scissors, and a 0.5 cm × 0.5 cm piece of the dermis layer was excised. The dermal chunks were transferred to a 1.5 mL microcentrifuge tube and minced into smaller pieces using micro-scissors. The minced tissue was placed in a sterile 30 mL beaker containing DMEM/F12 media with 0.14 Wunsch units/mL of Liberase Blendzyme 3 and 1% penicillin–streptomycin. The beaker was covered with foil and heated at 37 °C for 60 min on a magnetic hot plate. After enzymatic digestion, the media containing dermal tissue were transferred to a 50 mL conical tube, and 25 mL of fibroblast media (FM: 10% FBS and 1% P-S in DMEM/F12) was added. The mixture was triturated with a 10 mL serological pipette and centrifuged at 300× *g* for 5 min. The supernatant was removed, and the cells were resuspended in 10 mL of FM. The suspension was filtered through a 40 μm mesh and plated in a tissue-culture-treated T75 flask. Primary fibroblasts were allowed to adhere and expand for one week, with the medium replaced every 2–3 days until ~80% confluency was reached. The cultures were then passaged by aspirating the media, adding 3.5 mL of TrypLE Express, and incubating for 5 min at 37 °C. The reaction was inactivated by adding 10 mL of FM, and the cell suspension was centrifuged at 300× *g* for 5 min. After aspirating the supernatant, the cells were resuspended in 1 mL of FM for counting. The cells were seeded at 10 cells/μL in T75 flasks, fed with FM every 2–3 days, and passaged or frozen once 80% confluency was attained.

### 2.4. iPSC Reprogramming, Culture, and Differentiation

Fibroblasts were reprogrammed into iPSCs by episomal transfection [16] with 20 μg/mL episomal vectors (pCXLE-Oct3/4, pCXLE-hsk, pCXLE-hμl; Addgene, Watertown, MA, USA) in DMEM/F12 as the electroporation buffer. Secondary fibroblasts were pre-treated with 0.167 mg/mL valproic acid (Millipore Sigma, Burlington, MA, USA) for 2 h before passaging. For electroporation, 5 × 10^5^ cells were resuspended in 0.5 mL DMEM/F12, mixed with vectors, and electroporated at 200 V with two 20-ms pulses (Bio-Rad Gene Pulser Xcell, Hercules, CA, USA). Transfected cells were seeded onto Matrigel-coated 6-well plates in DMEM/F12 with 10% FBS, and the media were changed daily. From day 3, TeSR^TM^-E7^TM^ (STEMCELL Technologies, Vancouver, BC, Canada) was used for 21–28 days. iPSC colonies emerged within 3–4 weeks and were manually isolated and expanded into stable lines.

### 2.5. iPSC Primary Culture, Propagation and Freezing

Primary iPSCs were manually passaged onto Matrigel-coated 6-well plates. Fibroblasts were scraped away using a pipette tip, leaving intact iPSC colonies. The colonies were cut horizontally and vertically with a sterile 22-gauge needle, and aggregates were transferred individually to wells containing TeSR^TM^-E8^TM^ media (STEMCELL Technologies, Vancouver, BC, Canada). The plates were rocked to disperse colonies and incubated at 37 °C, with daily media changes for 5–7 days. Confluent iPSCs were passaged uSsing ReLeSR^TM^ (STEMCELL Technologies, Vancouver, BC, Canada) following the manufacturer’s protocol. Detached colonies were diluted (1:10–1:50) in TeSR^TM^-E8^TM^ and replated. Excess cells were centrifuged, resuspended in mFreSR^TM^ (STEMCELL Technologies, Vancouver, BC, Canada), and frozen in cryovials using a Mr. Frosty^TM^ container at −80 °C before transfer to liquid nitrogen. Tertiary iPSCs were cultured in TeSR^TM^-E8^TM^ with daily media changes for 5–7 days.

### 2.6. Generation and Differentiation of iPSC-Derived Forebrain and Spinal Cord NSPCs

Passage 5 iPSCs were differentiated into dorsal forebrain NSPCs (iPSC-Br) using the dual-SMAD inhibition protocol. iPSC colonies were cleaned of differentiated cells, dissociated with Accutase, and resuspended in TeSR^TM^-E8^TM^ for counting. The cells (1000 cells/μL) were seeded onto Matrigel-coated plates in STEMdiff^TM^ Neural Induction Media with 10 μM Y-27632 and fed daily for 7 days. The cells were passaged with Accutase and reseeded at lower densities (20 cells/μL) in STEMdiff^TM^ Neural Progenitor Medium for further development into a forebrain phenotype. Excess cells were cryopreserved in STEMdiff^TM^ Neural Progenitor Freezing Medium and stored in liquid nitrogen. For spinal cord-specific NSPCs (iPSC-SC), iPSCs were similarly dissociated and seeded into Matrigel-coated plates in Neural Induction Media (N2B27 with growth factors) at 100 cells/μL, fed daily for 5 days, and switched to Neural Maintenance Media for expansion over 18 days [29]. By day 28, iPSC-SC NSPCs were prepared for differentiation, proliferation assays, or RNA sequencing. Excess cells were cryopreserved as described for iPSC-Br NSPCs.

### 2.7. Proliferation and Differentiation of iPSC-Derived NSPCs

On day 28, iPSC-SC and iPSC-Br NSPCs were passaged with Accutase and seeded at 5 cells/μL in proliferation media (STEMdiff™ Neural Progenitor Medium for iPSC-Br; Neural Maintenance Medium for iPSC-SC) with 10 μM Y-27632. After two days, the media were replaced for proliferation (EFH) or differentiation (BrainPhys™ or SFM with 1% FBS for iPSC-Br; N2B27 or SFM with 1% FBS for iPSC-SC). The media were refreshed every 2–3 days for 1–2 weeks before fixation in 4% PFA. Fixed cells were blocked, permeabilized, and stained with primary antibodies (e.g., O4, β-III tubulin, GFAP, Nestin, Sox2, BrdU) and Alexa-Fluor-conjugated secondary antibodies. Hoechst counterstaining was performed for immunofluorescence analysis. For all assays, three biological replicates (H17, H18, H25) were used, and each functional assay (e.g., percentage of β-III tubulin+ cells) included three technical replicates. Each technical replicate consisted of 10 standardized images captured from a 96-well plate.

### 2.8. RNA Sequencing and Data Analysis

The RNA sequencing analysis was conducted on five adult human donors (H17, H18, H25, H38, and H39). RNA was extracted using the RNeasy Micro Kit (Qiagen, Germantown, MD, USA), yielding 20 µL of RNA in RNase-free water, which was stored at −20 °C. RNA concentration was measured with NanoDrop™, and the quality was verified using a Fragment Analyzer, selecting only samples with RQN values above seven for sequencing. For RNA sequencing, 50 ng of RNA per sample was sent to StemCore Laboratories, Ottawa, ON, Canada for library preparation and sequencing on the NextSeq platform. Quality control of the sequencing data was conducted using FastQC, with reads mapped to the human genome/transcriptome via HISAT2. Picard’s CollectRnaSeqMetrics tool was employed to determine the fraction of reads mapping to coding and non-coding sequences. Quantification of transcripts was performed using Salmon, utilizing human transcript sequences from GENCODE v35. Post-quantification, data for 24,006 genes with five or more counts in at least two NSPC types were retained and combined into a single DESeq2 dataset. Differential gene expression between NSPC types was analyzed using DESeq2, with rlog-transformed normalized counts used for principal component analysis (PCA) and hierarchical clustering. PCA was based on the 500 most variable genes, while hierarchical clustering employed Euclidean distance between rlog-transformed counts. DESeq2 provided fold changes and *p*-values, with significance adjusted via the Benjamini–Hochberg method to calculate the false discovery rate (FDR), considering genes with a 5% FDR as differentially expressed. Visualization of DE genes was achieved through volcano plots and heatmaps, and scatter plots were generated for all genes and protein-coding genes specifically. Gene set enrichment analysis (GSEA) was conducted to identify functional changes in gene expression. Genes were ranked by log2FoldChange for comparisons between NSPC types, with GSEA run against subsets of gene sets from MSigDB, including hallmark gene sets, curated gene sets, canonical pathways, Gene Ontology annotations, and cell type signatures. STRING v12.0 software was used to create protein–protein interaction networks for the 30 most upregulated and 30 most downregulated genes in iPSC-derived NSPCs compared to bona fide NSPCs, using a minimum interaction score of 0.400 and a PPI enrichment *p*-value < 1.0 × 10⁻¹⁶ with network maps displaying a maximum of 20 indicators in the first shell and 5 in the second. 

## 3. Results

### 3.1. iPSC-Br NSPCs Exhibit Closer Morphological and Marker Resemblance to Bona Fide NSPCs

**Figure 1:**
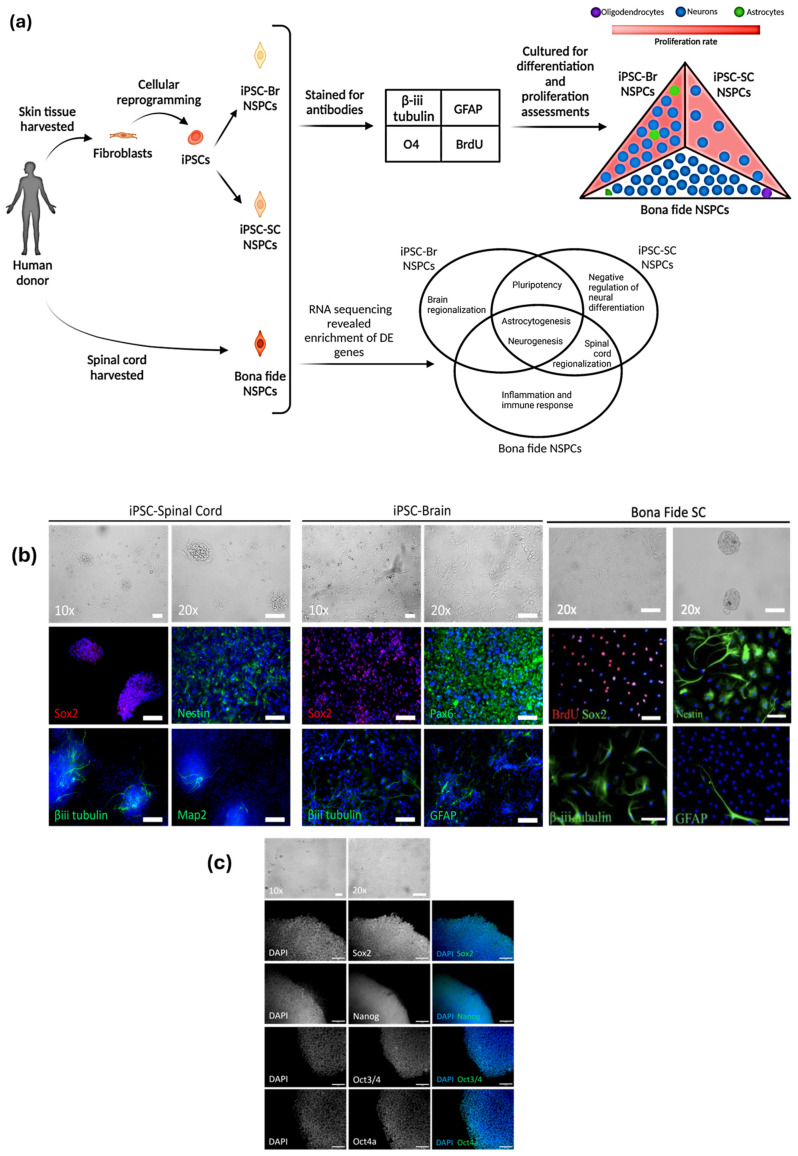
Differentiation, proliferation, and characterization of NSPCs derived from iPSCs and bona fide source. iPSC-Br NSPCs more closely resemble bona fide NSPCs in morphology and marker expression compared to iPSC-SC NSPCs. (**a**) Schematic illustration of the experimental workflow. Skin tissue samples from human donors were cultured into primary fibroblasts and reprogrammed into iPSCs. (**b**) Morphological and immunocytochemical analysis of NSPCs. iPSC-SC NSPCs form flat and neurosphere-like colonies, expressing Sox2, Nestin, β-III tubulin, and Map2. iPSC-Br NSPCs exhibit multipolar spindle-like cells, expressing Sox2, Pax6, β-III tubulin, and GFAP. Bona fide spinal cord NSPCs form adherent layers and neurosphere-like colonies, expressing Nestin, Sox2, β-III tubulin, and GFAP. (**c**) Pluripotency markers (Sox2, Nanog, Oct3/4, Oct4a) in iPSC-SC colonies confirm retention of stemness. DAPI shows nuclei in all panels.

The schematic overview presented in Figure 1a outlines the experimental workflow, demonstrating how fibroblasts derived from human skin tissue samples were successfully reprogrammed into induced pluripotent stem cells (iPSCs) and subsequently differentiated into neural stem/progenitor cells (NSPCs) with brain (iPSC-Br) or spinal cord (iPSC-SC) phenotypes. The generated iPSC colonies, shown in Figure 1b, exhibited sharply defined boundaries and a densely packed cellular architecture, characterized by a high nucleus-to-cytoplasm ratio indicative of their pluripotent, undifferentiated state. Immunocytochemical staining (Figure 1c) confirmed the expression of key pluripotency markers, including Oct3/4, Oct4a, Nanog, and Sox2, validating the colonies’ pluripotent identity. Upon differentiation, iPSC-Br NSPCs, developed via dual SMAD inhibition, displayed a uniform multipolar spindle-like morphology (Figure 1b), while iPSC-SC NSPCs, differentiated through a neuro-mesodermal progenitor (NMP) intermediate, formed both flat colonies and neurosphere-like structures. Bona fide spinal cord NSPCs resembled iPSC-SC NSPCs in forming neurosphere-like colonies under the same conditions, while both iPSC-Br NSPCs and bona fide NSPCs formed adherent layers when grown on Matrigel. Additionally, all NSPCs, including bona fide, iPSC-Br, and iPSC-SC, formed neurospheres when grown in the suspension culture.

Immunocytochemistry confirmed Sox2 expression across all NSPC types (Figure 1b), highlighting their neural stem/progenitor cell identity. Negative staining revealed that Nestin was expressed in iPSC-SC and bona fide NSPCs but was absent in iPSC-Br NSPCs, while GFAP expression was observed exclusively in iPSC-Br and bona fide NSPCs, and absent in iPSC-SC NSPCs (Supplementary Figure S1). Neuronal differentiation potential was evident in all NSPC types, as β-III tubulin and Map2 expression confirmed the generation of neurons from these cells (Figure 1b). Astrocytic differentiation, marked by GFAP expression, was observed exclusively in iPSC-Br and bona fide NSPCs, while the absence of GFAP expression in iPSC-SC NSPCs is demonstrated in Supplementary Figure S1. Interestingly, Pax6 expressions were detected only in iPSC-Br NSPCs (Figure 1b), aligning with their dorsal forebrain identity. The distinct morphological and marker profiles highlight the unique differentiation pathways and regional identities of these NSPC populations. Overall, these findings emphasize the ability of iPSCs to generate NSPCs with spinal-cord-specific traits, positioning iPSC-derived NSPCs as a promising resource for spinal cord repair therapies.

### 3.2. IPSC-Br NSPCs Exhibit Increased Neurogenic Differentiation Potential and Proliferation Rates

**Figure 2:**
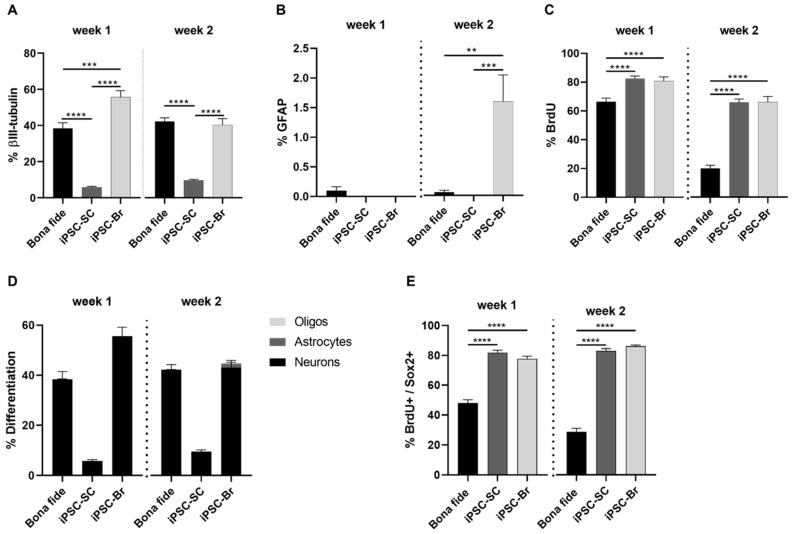
Comparative analysis of differentiation and proliferation profiles between bona fide and iPSC-derived NSPC. iPSC-Br NSPCs showed greater neuronal and astrocyte differentiation and higher proliferation rates than both iPSC-SC and bona fide NSPCs, indicating their superior neurogenic potential. (**A**) The percentage of β-III tubulin+ neurons was significantly higher in bona fide, and iPSC-Br NSPCs compared to iPSC-SC NSPCs at both time points. (**B**) By week two, GFAP+ astrocyte differentiation was markedly higher in iPSC-Br NSPCs compared to bona fide and iPSC-SC NSPCs, with minimal astrocytic differentiation observed in the latter. (**C**) Proliferation rates, measured as BrdU+ cell percentages, were significantly higher in iPSC-derived NSPCs compared to bona fide NSPCs under differentiation conditions. (**D**) Lineage differentiation profiles revealed that iPSC-Br NSPCs exhibited contributions from neuronal, astrocytic, and minimal oligodendrocytic lineages, while iPSC-SC NSPCs were predominantly neuronal. Bona fide NSPCs were also predominantly neuronal with negligible astrocytic and almost no oligodendrocytic differentiation. (**E**) Self-renewal capacity, as indicated by BrdU+/Sox2+ cells, was higher in iPSC-derived NSPCs compared to bona fide NSPCs at both time points. Data are presented as mean ± s.e.m.; ** *p* < 0.01, *** *p* < 0.001, **** *p* < 0.0001; n = 3 for bona fide, iPSC-SC, and iPSC-Br NSPCs. Each assay was conducted with three biological replicates (H17, H18, H25), and each functional assay, such as the percentage of β-III tubulin + cells, was performed with three technical replicates. Each technical replicate involved capturing 10 standardized images from a 96-well plate.

The differentiation and proliferation capacities of iPSC-Br, iPSC-SC, and bona fide spinal cord NSPCs were evaluated under identical culture conditions over two weeks. Neuronal differentiation, assessed by β-III tubulin expression, was significantly higher in iPSC-Br NSPCs compared to bona fide and iPSC-SC NSPCs at week one (bona fide: ~25%, iPSC-SC: ~5%, iPSC-Br: ~70%) (Figure 2A). By week two, β-III tubulin+ neuronal differentiation in iPSC-Br NSPCs decreased slightly (~40%) but remained comparable to bona fide NSPCs (~45%), whereas iPSC-SC NSPCs continued to exhibit significantly lower levels (~10%) (Figure 2A). Astrocytic differentiation, indicated by GFAP expression, was markedly higher in iPSC-Br NSPCs (~2.5% by week two) compared to bona fide NSPCs (~0.05%), with no astrocytic differentiation observed in iPSC-SC NSPCs (Figure 2B). Proliferation rates, assessed by BrdU incorporation, revealed robust activity in all NSPC populations at week one, with iPSC-SC and iPSC-Br NSPCs exhibiting higher rates (~83% and ~80%, respectively) than bona fide NSPCs (~66%) (Figure 2C). By week two, proliferation declined across all groups, but iPSC-derived NSPCs maintained significantly higher rates (~66%) compared to bona fide NSPCs (~20%) (Figure 2C). To further explore individual donor-specific patterns, we presented detailed differentiation and proliferation profiles for NSPCs derived from donors H17, H18, and H25 (Supplementary Figure S2). These profiles illustrate that iPSC-Br NSPCs consistently demonstrate enhanced neuronal and astrocytic differentiation and higher proliferation rates across donors, closely mirroring bona fide NSPCs, while iPSC-SC NSPCs exhibit lower differentiation and proliferation capacities.

Furthermore, lineage differentiation profiles revealed that iPSC-Br NSPCs exhibited a higher proportion of neuronal differentiation alongside some astrocytic and minimal oligodendrocytic contributions. In contrast, iPSC-SC NSPCs demonstrated predominantly neuronal differentiation with negligible astrocytic and no detectable oligodendrocytic differentiation, while bona fide NSPCs were predominantly neuronal with very limited astrocytic and almost no oligodendrocytic differentiation (Figure 2D). Under self-renewing conditions supplemented with mitogens epidermal growth factor (EGF) and fibroblast growth factor (FGF), along with the FGF cofactor heparin, both iPSC-Br and iPSC-SC NSPCs exhibited significantly higher BrdU+/Sox2+ proliferation rates than bona fide NSPCs at both week one (~83% and ~78% vs. ~48%) and week two (~83–86% vs. ~29%) (Figure 2E). In summary, iPSC-Br NSPCs exhibited superior developmental capacities compared to iPSC-SC NSPCs, including robust neuronal and astrocytic differentiation, higher and sustained proliferation rates, and the ability to generate diverse neural lineages, closely mirroring bona fide NSPCs. These findings highlight iPSC-Br NSPCs as a promising potential model for neural biology research and regenerative therapies, offering valuable insights into cell differentiation and proliferation dynamics under defined culture conditions.

### 3.3. iPSC-Br NSPCs Exhibit Transcriptomic Parity with Bona Fide NSPCs

**Figure 3:**
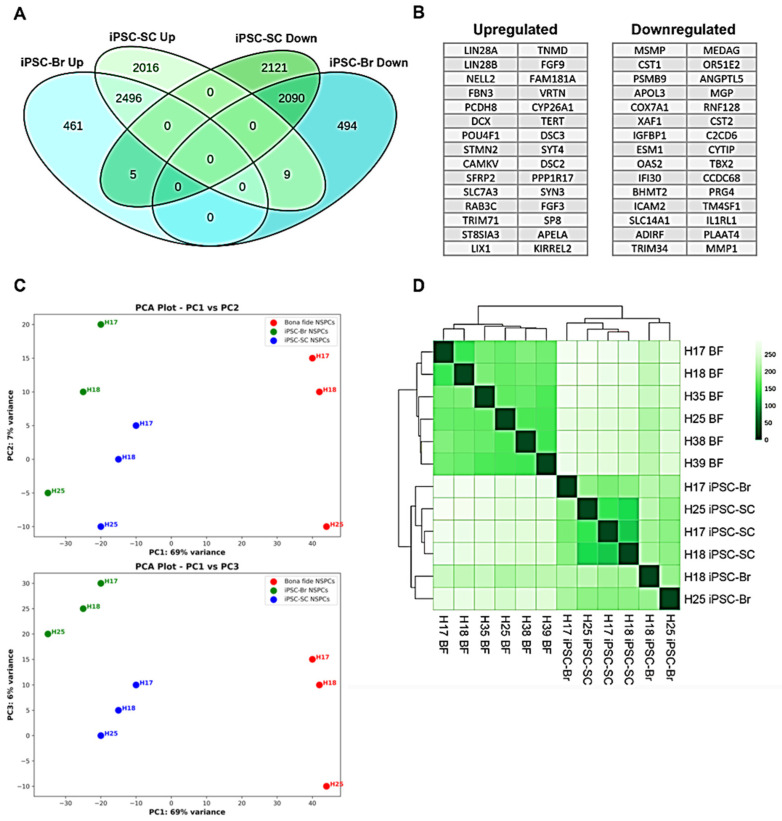
The transcriptomes of iPSC-Br and bona fide NSPCs are more similar than iPSC-SC and bona fide NSPCs. The gene expression analysis reveals that iPSC−Br NSPCs retain transcriptomic features similar to bona fide NSPCs, unlike the more distinct iPSC−SC NSPCs (**A**) Venn diagram of DE genes in both iPSC−SC and iPSC−Br NSPCs relative to bona fide NSPCs, including directionality (upregulation or downregulation). A total of 4600 genes were DE in both iPSC−SC and iPSC−Br NSPCs with a fold change ≥ 2 and q-value < 0.05. (**B**) The top 30 genes DE in the same direction (upregulation or downregulation) in both iPSC−SC and iPSC−Br NSPCs relative to bona fide NSPCs. (**C**) PCA of the 500 most variable genes across bona fide, iPSC-SC, and iPSC-Br NSPCs. Each dot represents a sample (H17, H18, H25), with donor-specific identifiers labeled. PC1 (69% variance) explains the largest transcriptomic differences, while PC2 (7% variance) and PC3 (6% variance) capture additional variation. iPSC-Br NSPCs cluster closer to bona fide NSPCs than iPSC-SC NSPCs, reflecting greater transcriptomic similarity. Donor-specific differences are evident along PC2, while PC3 highlights intrinsic differences between iPSC-SC and iPSC-Br NSPCs. All differences are statistically significant, * *p* < 0.05 for all comparisons. (**D**) Hierarchical clustering analysis for all transcripts (24,006 genes) found in common among bona fide, iPSC−SC, and iPSC−Br NSPCs. The clustering illustrates that bona fide NSPCs group closely together, with iPSC-Br NSPCs forming a cluster more similar to bona fide NSPCs than iPSC-SC NSPCs. The intensity of the green shading reflects expression levels, with darker shades indicating higher expression.

To investigate the transcriptomic differences among bona fide NSPCs, iPSC-derived NSPC-Spinal Cord (iPSC-SC), and iPSC-derived NSPC-Brain (iPSC-Br), we performed RNA sequencing analysis on three NSPC types, derived from five different donors (H17, H18, H25, H38, and H39). A total of approximately 24,006 genes were analyzed across all samples. Comparative analysis revealed that 4600 genes (19% of the total) were differentially expressed (DE) in both iPSC-SC and iPSC-Br NSPCs relative to bona fide NSPCs (Figure 3A). Among the DE genes, 2496 were upregulated, indicating higher activity in the iPSC-derived NSPCs compared to bona fide NSPCs, while 2090 were downregulated, indicating lower activity. Interestingly, 14 genes exhibited differential expression in opposite directions, being more active in one type of iPSC-derived NSPC and less active in the other. Among the top 30 upregulated and downregulated protein-coding genes, the pluripotency markers LIN28A and LIN28B were highly enriched in both iPSC-SC and iPSC-Br NSPCs compared to bona fide NSPCs, indicating the retention of pluripotent characteristics in the iPSC-derived NSPCs (Figure 3B).

The first PCA plot, constructed using the PC1 (69% variance) and PC2 (7% variance) axes, demonstrated a clear separation of the NSPC types primarily along the PC1 axis. This analysis revealed that iPSC-derived NSPCs (iPSC-SC and iPSC-Br) are more similar to each other than to bona fide NSPCs, likely reflecting shared transcriptomic characteristics linked to their reprogrammed origin and residual pluripotency-associated gene expression. Despite this overarching similarity, iPSC-Br NSPCs exhibited a closer proximity to bona fide NSPCs compared to iPSC-SC NSPCs (Figure 3C, upper panel). Analysis along the PC2 axis revealed additional variance linked to donor-specific differences. For example, NSPCs derived from donor “H17” consistently displayed the highest scores on PC2, whereas those from donor “H25” had the lowest, except for bona fide NSPCs from donors “H17” and “H18”, where the order was reversed. This suggests that some of the transcriptomic variation between NSPC types is influenced by the donor origin, likely due to inherent genetic variability or specific epigenetic factors unique to each donor. In contrast, when examining the PCA plot using the PC1 (69% variance) and PC3 (6% variance) axes, a distinct separation between iPSC-SC and iPSC-Br NSPCs emerged, highlighting transcriptomic differences between these two iPSC-derived cell types. Notably, the donor-specific associations observed along the PC2 axis were no longer evident in this configuration, indicating that the variation captured by PC3 reflects intrinsic differences between iPSC-derived NSPCs rather than donor-specific factors (Figure 3C, lower panel). Hierarchical clustering of gene expression data (Figure 3D) further emphasized the distinction between bona fide NSPCs and iPSC-derived NSPCs while also illustrating the shared transcriptomic similarities between iPSC-SC and iPSC-Br NSPCs. The raw analysis data has been submitted in Supplementary File S1.

These results suggest that while iPSC-derived NSPCs retain certain pluripotency-associated characteristics and exhibit shared transcriptomic features distinct from bona fide NSPCs, iPSC-Br NSPCs demonstrate a closer resemblance to bona fide NSPCs. This nuanced understanding highlights the need for further optimization of differentiation protocols to reduce the transcriptomic divergence between iPSC-derived and bona fide NSPCs.

**Figure 4:**
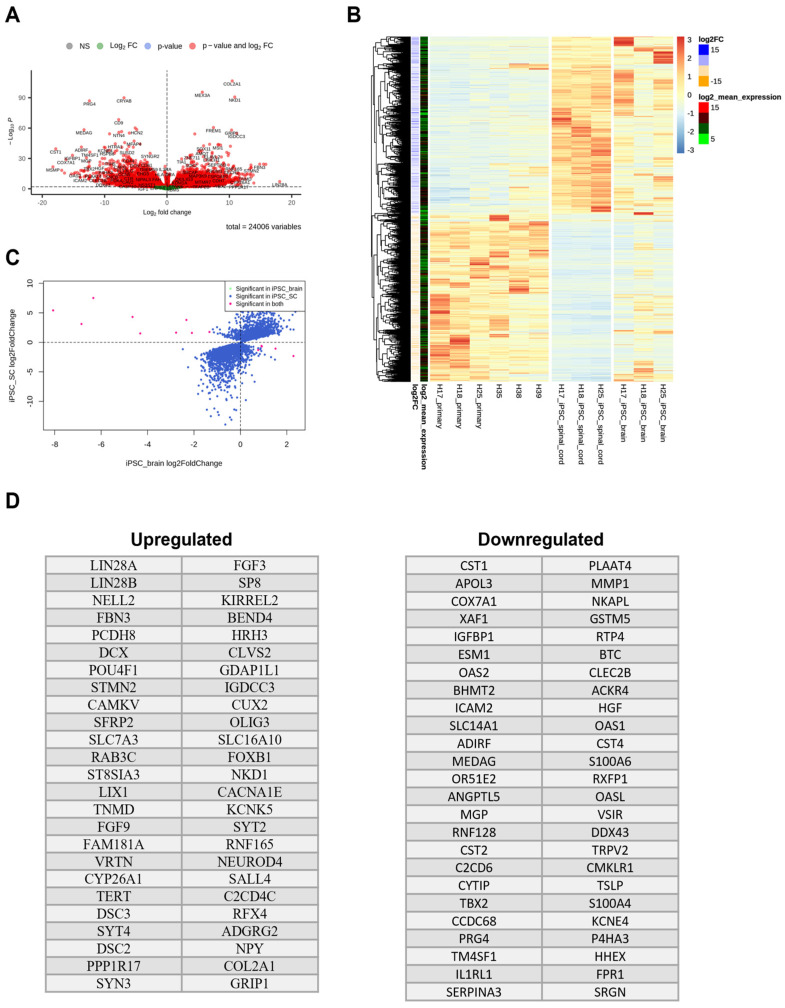
Comparative gene expression analysis of iPSC-derived NSPCs and bona fide NSPCs. iPSC-SC NSPCs may reflect a distinct reprogramming pathway compared to iPSC-Br NSPCs. (**A**) Volcano plots show differentially expressed (DE) genes between iPSC-SC NSPCs (left panel) and iPSC-Br NSPCs (right panel) relative to bona fide NSPCs, with red dots indicating significant DE genes (fold change ≥ 2, *p*-value < 0.05). (**B**) Heatmaps with hierarchical clustering depict gene expression patterns, comparing iPSC-SC NSPCs (left panel) and iPSC-Br NSPCs (right panel) to bona fide NSPCs. (**C**) Scatter plots highlight genes uniquely differentially expressed in iPSC-SC NSPCs (left panel) and iPSC-Br NSPCs (right panel) compared to bona fide NSPCs. (**D**) Lists of the top 50 upregulated and downregulated genes in iPSC-SC NSPCs and iPSC-Br NSPCs, each relative to bona fide NSPCs.

Differentially expressed genes in brain-derived iPSC-Br NSPCs closely resembled bona fide NSPCs in their gene expression profiles compared to spinal cord-derived iPSC-SC NSPCs. The volcano plot in Figure 4A emphasizes these distinctions by comparing the unique gene expression patterns in iPSC-SC and iPSC-Br NSPCs relative to bona fide NSPCs. Heatmaps of the DE genes, shown in Figure 4B, further illustrate these findings, with hierarchical clustering revealing that iPSC-Br NSPCs (right panel) exhibit a gene expression pattern more similar to bona fide NSPCs, while iPSC-SC NSPCs (left panel) display distinct clusters of upregulated and downregulated genes. The scatter plot in Figure 4C highlights the red dots representing genes that are significantly differentially expressed (DE), meeting predefined thresholds for statistical significance, and showcasing substantial transcriptional variation between the NSPC types.

Interestingly, 83% of the DE genes in iPSC-Br NSPCs were also differentially expressed in iPSC-SC NSPCs, indicating a substantial overlap in transcriptomic changes between these two iPSC-derived NSPC populations. Additional insights into the differential gene expression between iPSC-Br NSPCs and bona fide NSPCs are provided in Supplementary Figure S3, where 5555 DE genes were identified.

The top 50 upregulated and downregulated genes in iPSC-SC NSPCs and iPSC-Br NSPCs, relative to bona fide NSPCs, are shown in Figure 4D, respectively. Among the most enriched genes in iPSC-derived NSPCs are the pluripotency markers *LIN28A* and *LIN28B*, which are RNA-binding proteins critical for maintaining pluripotency and regulating developmental stages [30,31]. Their significantly higher expression in iPSC-SC NSPCs indicates that these cells may retain residual pluripotent characteristics, potentially affecting their differentiation potential and self-renewal capacity. Additionally, POU4F1 (BRN3A), a transcription factor associated with neural crest stem cells and early neural development [32], is more enriched in iPSC-SC NSPCs compared to iPSC-Br NSPCs and bona fide NSPCs, indicating a predisposition toward neural crest lineage differentiation. The transcriptomic profiles of iPSC-Br, closely resembling those of bona fide NSPCs, provide a critical molecular framework for studying neural differentiation and lineage identity. Differentially expressed genes reveal key regulatory pathways and gene networks involved in neural development, including those governing lineage specification, self-renewal, and differentiation potential. This genetic insight allows for the exploration of transcriptional hierarchies and signaling cascades that drive neural cell fate decisions, advancing our understanding of neural biology and informing strategies for regenerative medicine and disease modeling.

### 3.4. Transcriptomic Divergence and Pluripotency Retention in iPSC-Derived NSPCs Relative to Bona Fide NSPCs

**Figure 5:**
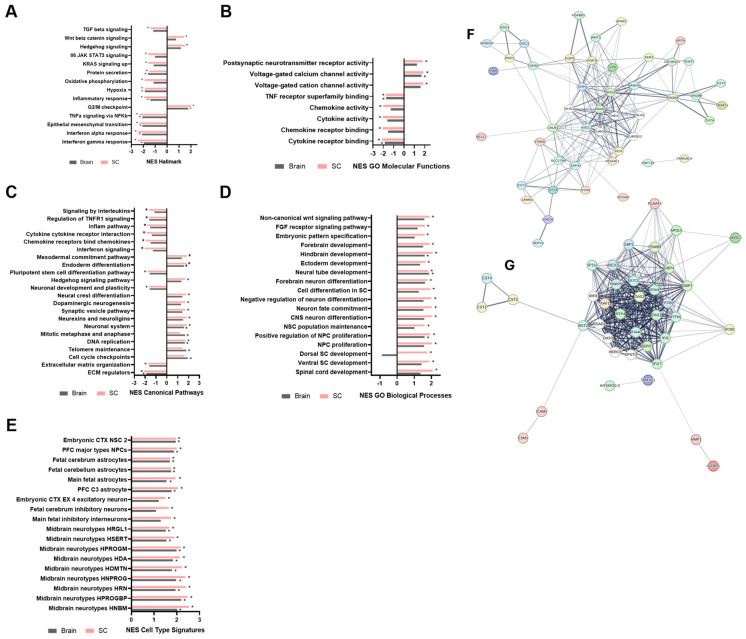
Comparative gene set enrichment in iPSC-derived NSPCs relative to bona fide NSPCs. Gene set enrichment analysis was conducted using the GSEA Preranked algorithm against a subset of MSigDB gene sets. A normalized enrichment score (NES) was used to compare gene sets across NSPCs in the categories of (**A**) hallmark gene sets, (**B**) GO molecular functions, (**C**) canonical pathways, (**D**) GO biological processes, and (**E**) cell-type signatures. Gene sets with significant positive or negative NES are indicated by *, representing a q-value < 0.05. iPSC-SC (n = 3) and iPSC-Brain (n = 3) are represented by pink and gray bars, respectively. Protein–protein interaction network maps of the top 30 (**F**) upregulated and (**G**) downregulated DE genes in both iPSC-SC and iPSC-Br NSPCs relative to bona fide NSPCs were generated using STRING v12.0 software (PPI enrichment *p*-value < 1 × 10⁻¹⁶). Each node represents proteins from a single, protein-coding gene locus, with colored nodes indicating query proteins and first-shell interactions, and white nodes representing second-shell interactions. Edge confidence is proportional to line thickness, indicating the strength of protein–protein associations.

Our study evaluated key expression markers associated with neural stem/progenitor cell (NSPC) identity, proliferation, neurogenesis, and gliogenesis (Figure 5A–E), revealing significant differences between induced pluripotent stem-cell-derived spinal cord (iPSC-SC) and brain (iPSC-Br) NSPCs. Both iPSC-derived NSPCs exhibited enrichment in essential NSPC markers such as *CD24*, *MSI1*, *SOX2*, *NES*, and *PROM1*, confirming their robust stem cell identity. *CD24* is a cell surface glycoprotein implicated in cell adhesion and signaling, commonly used as a marker to identify and isolate NSPCs [33]. *MSI1* (*Musashi-1*) is an RNA-binding protein that plays a crucial role in maintaining stem cell self-renewal and regulating differentiation, highly expressed in neural progenitor cells and essential for normal brain development [34]. In our study, we also observed that iPSC-SC NSPCs exhibited unique enrichment in *BMI1*, a polycomb group protein essential for the self-renewal and proliferation of neural stem cells [35]. Conversely, iPSC-Br NSPCs displayed relatively lower expression of EGFR compared to established NSPC profiles. EGFR plays a significant role in brain development, being expressed in regions where neuronal and glial cells are generated and is essential for the proliferation of multipotent neural precursors [36]. Distinct gene expression patterns were also observed in proliferation markers, with iPSC-derived NSPCs demonstrating enrichment in *TENM2*, *BCAT1*, *DCC*, *BCAR3*, and *CDK6*. *TENM2*, a transmembrane protein involved in cell signaling and adhesion, plays a crucial role in regulating neural progenitor proliferation and differentiation, essential for early neural development. Similarly, *BCAT1*, an enzyme pivotal in branched-chain amino acid metabolism, supports the metabolic demands of proliferating neural stem cells [37]. The elevated expression of these genes suggests that iPSC-derived NSPCs are primed for self-renewal and proliferation, underlying their potential utility in regenerative applications targeting neural tissue repair. To further investigate transcriptomic divergence between iPSC-derived NSPCs and bona fide NSPCs, we analyzed differential gene expression for key marker categories, as shown in Supplementary Figure S4. The differential gene expression analysis highlights distinct functional characteristics across iPSC-Br, iPSC-SC, and bona fide NSPCs. iPSC-Br NSPCs exhibit gene expression profiles that more closely resemble bona fide NSPCs, particularly for neurogenesis markers such as Doublecortin (DCX) and Neurogenic Differentiation 1 (NEUROD1)*,* and gliogenesis markers, including *Glial Fibrillary Acidic Protein* (*GFAP*) for astrogenesis and SRY-Box Transcription Factor 10 (SOX10) for oligodendrogenesis, depicting their enhanced neurogenic and glial differentiation potential [38,39]. Conversely, iPSC-SC NSPCs are enriched in proliferation-associated markers like Marker of Proliferation Ki-67 (MKI67), reflecting a stronger proliferative capacity but reduced neurogenic potential [38]. Markers indicative of general neural stem cell (NSC) identity, such as *Nestin* (*NES*) and *SRY-Box Transcription Factor 2* (*SOX2*), are consistently expressed across all NSPC types [38,39]. However, iPSC-SC NSPCs display unique upregulation of *Prominin-1* (*PROM1*), also known as *CD133*, highlighting a bias toward self-renewal over differentiation. CD133, a transmembrane glycoprotein, is widely regarded as a marker of normal and cancerous stem cells, particularly in the central nervous system (CNS) [40]. In neural stem cells, CD133 is associated with preserving self-renewal capacity and promoting migration during development, underscoring its role in maintaining stemness [40,41]. These results emphasize the therapeutic advantages of iPSC-Br NSPCs for applications requiring neural regeneration and differentiation, while further optimization is needed for iPSC-SC NSPCs to replicate the functional versatility of bona fide NSPCs.

Gene set enrichment analysis (GSEA) of differentially expressed genes provided key insights into the developmental and functional distinctions between iPSC-SC and iPSC-Br NSPCs (Figure 5F,G). iPSC-SC NSPCs exhibited significant enrichment in Wnt/β-catenin and FGFR signaling pathways, with notable downregulation of TGFβ signaling, suggesting a more primitive or early developmental stage [42,43]. iPSC-SC NSPCs also displayed unique enrichment in postsynaptic neurotransmitter receptor activity, indicating distinct functional properties [44]. In contrast, iPSC-Br NSPCs showed enrichment in mature neural pathways, including those involved in forebrain, hindbrain, and diencephalon development. These features, along with enrichment in brain-specific gene sets, suggest that iPSC-Br NSPCs align more closely with neural characteristics typically associated with bona fide NSPCs described in prior studies. Both iPSC-Br and iPSC-SC NSPCs shared enrichment in voltage-gated calcium channel activity, essential for neural signaling [45]. While iPSC-SC NSPCs showed distinct enrichment in early developmental processes, such as neural crest differentiation, iPSC-Br NSPCs displayed stronger resemblance to mature neural characteristics, highlighting their potential for modeling neural development and therapeutic strategies. Oligodendrocyte differentiation processes were notably enriched in iPSC-derived NSPCs, particularly through genes like *OLIG2*, *PAX6*, and *ASCL1* [45,46]. Network analysis further distinguished iPSC-SC NSPCs, with downregulation of pathways involved in ribonuclease activity, interferon signaling, and immune responses. The results signify the distinct developmental and functional profiles of iPSC-SC and iPSC-Br NSPCs, with bonafide NSPCs demonstrating greater alignment with neural maturity.

## 4. Discussion

The role of neural stem/progenitor cells (NSPCs) in neural development and repair has long been a focal point in neurobiology, highlighting the significance of their molecular and functional attributes [47,48,49]. In the current study, a notable similarity was observed between iPSC-Br NSPCs, which exhibit forebrain characteristics, and bona fide spinal cord NSPCs, particularly in their transcriptomic profiles and differentiation capacities. The RNA sequencing data indicated that iPSC-Br NSPCs shared 83% of differentially expressed (DE) genes with bona fide NSPCs, suggesting a substantial overlap in the transcriptomic signatures critical for neurogenesis and the maintenance of neural progenitors. This significant overlap points to the ability of iPSC-Br NSPCs to effectively mimic key neural developmental processes, including the activation of neurogenic pathways and the consistent expression of essential stem cell markers like Nestin and Sox2, found in both iPSC-Br and bona fide NSPCs. During differentiation studies, iPSC-Br NSPCs demonstrated uniform morphology and heightened neurogenic potential, as evidenced by the presence of GFAP+ astrocytes and the expression of Pax6, a marker linked to dorsal forebrain identity. In contrast, iPSC-SC NSPCs exhibited more variability in morphology and a diminished capacity for neuronal differentiation. While both iPSC-SC and bona fide NSPCs expressed the neural progenitor marker Nestin, iPSC-SC NSPCs showed reduced neuronal differentiation. This reduction may be attributed to the enrichment of genes in iPSC-SC NSPCs that primarily act to negatively regulate neuron differentiation, raising concerns about their potential efficacy in spinal cord injury (SCI) repair. Moreover, iPSC-SC NSPCs exhibited tighter clustering in their gene expression profiles, indicating a more homogeneous transcriptional identity. However, iPSC-Br NSPCs displayed a closer resemblance to bona fide NSPCs, particularly in samples from specific donors such as H18, suggesting that iPSC-Br cells retain critical transcriptomic features that more accurately reflect those of bona fide adult NSPCs. In addition to that, iPSC-SC NSPCs exhibited elevated expression of *POU4F1*, also known as *BRN3A*, a transcription factor from the POU domain family that plays a pivotal role in the development and differentiation of sensory neurons within the neural crest lineage [32]. *POU4F1* is also implicated in regulating neuronal survival and axon growth [50], and its heightened expression in iPSC-SC NSPCs suggests that these cells may retain traits characteristic of an earlier developmental stage. This developmental retention likely contributes to their divergence from bona fide NSPCs and may underline their less favorable differentiation outcomes.

The closer resemblance of iPSC-Br NSPCs to bona fide NSPCs is influenced by multiple factors, including donor-specific genetic and epigenetic landscapes. While previous studies have highlighted the functional similarities of iPSC-derived NSPCs to neural stem/progenitor cells, no studies till date have explored their ability to fully mimic bona fide NSPCs within syngeneic adult human populations [20,21,51]. Notably, in our study, iPSC-Br NSPCs derived from human donor H18 exhibited transcriptomic variations that closely mirrored those of bona fide NSPCs, particularly in the expression of critical neurogenic markers such as Nestin and Sox2, highlighting their capacity to replicate the molecular and functional attributes essential for neural development and repair. These findings position iPSC-Br NSPCs as a promising candidate for regenerative therapies, given their ability to emulate the fundamental characteristics of bona fide NSPCs. In contrast, iPSC-SC NSPCs derived from donor H18 demonstrated more pronounced transcriptomic differences, particularly in pathways linked to neurogenesis and extracellular matrix interactions, which may limit their capacity to fully replicate the properties of bona fide NSPCs. Although this divergence was less evident in certain cases, such as with donor H25, the overall findings reinforce the superior resemblance of iPSC-Br NSPCs to bona fide NSPCs. These results also emphasize the critical role of donor-specific genetic and epigenetic landscapes in shaping the molecular and functional profiles of iPSC-derived NSPCs, highlighting the need to consider individual variations when optimizing regenerative therapies. 

The closer resemblance of iPSC-Br NSPCs can also be attributed to their intrinsic plasticity, preserved regional specification signaling, and transcriptional and epigenetic landscapes, which collectively enhance their ability to replicate neural developmental processes, particularly neuronal and astrocytic differentiation. Region-specific differentiation protocols further strengthen their fidelity to bona fide NSPCs, supporting their use in therapeutic and modeling applications. In contrast, iPSC-SC NSPCs face challenges in replicating bona fide NSPCs due to the unique biochemical and biomechanical properties of the spinal cord. The extracellular matrix components, growth factors, and signaling molecules critical to spinal cord NSPC function may not be fully replicated in vitro, contributing to disparities in differentiation and proliferative potential. These observations emphasize that the success of iPSC-derived NSPCs depends not only on region-specific protocols and cellular characteristics but also on donor-specific factors that shape their developmental and functional profiles. Recognizing and addressing these patient-specific variables is essential for advancing the therapeutic potential of iPSC-derived NSPCs and achieving outcomes that closely mimic bona fide NSPCs in neural regeneration.

In conclusion, this study systematically compared iPSC-derived spinal cord neural stem/progenitor cells (iPSC-SC NSPCs), forebrain NSPCs (iPSC-Br NSPCs), and bona fide spinal cord NSPCs to evaluate their molecular, functional, and morphological characteristics. The results identify iPSC-Br NSPCs as a promising model for neural research, exhibiting greater similarity to bona fide spinal cord NSPCs. These findings highlight the need for continued refinement of differentiation protocols, robust molecular profiling, and functional validation to enhance the therapeutic potential of iPSC-derived models for spinal cord injury. Future directions for stem cell therapies should prioritize addressing critical challenges, including patient-specific and region-specific variability. Tackling these issues is essential to overcoming the limitations that have historically contributed to clinical setbacks and advancing the development of more effective and personalized treatment approaches. Overall, this study provides valuable insights into optimizing iPSC-derived therapies for spinal cord injury repair by incorporating patient-specific and transcriptomic considerations, and also highlights the need for further investigation to refine treatments tailored to patient-specific factors.

## Data Availability

The original contributions presented in this study are included in the article/Supplementary Materials. Further inquiries can be directed to the corresponding authors.

## Supplementary Materials

The following supporting information can be downloaded at: https://www.mdpi.com/article/10.3390/cells14020064/s1. Supplementary File S1: The raw analysis data, including graphs, for RNA sequencing and differential gene expression analysis of bona fide, iPSC-SC, and iPSC-Br NSPCs has been compiled in this file. Figure S1: Negative Staining Validation for Neural Stem/Progenitor Cell Markers in iPSC-Derived and Bona Fide NSPCs. Figure S2: Donor-Specific Differentiation and Proliferation Profiles of Bona Fide and iPSC-Derived NSPCs. Figure S3: iPSC-Br and bona fide NSPCs differentially express 5555 genes. Figure S4: Differential Expression of Genetic Markers for NSPCs and Functional Pathways Across Bona Fide, iPSC-SC, and iPSC-Br NSPCs.
