# Supplementary material for: Transcriptomic and Functional Landscape of Adult Human Spinal Cord NSPCs Compared to iPSC-Derived Neural Progenitor Cells
Source: Cells. 2025 Jan 7;14(2):64. doi: 10.3390/cells14020064 (PMC11763936; doi:10.3390/cells14020064)
Supplement: Supplementary file 1 [file cells-14-00064-s001.zip › cells-3390501-supplementary file S1.pdf]

(http://multiqc.info)

A modular tool to aggregate results from bioinformatics analyses across many samples into a single report.

Report generated on 2021-11-25, 14:49 based on data in: `/global/online/ohri/ohri1/projects/ahmad_galuta/human_spinal_cord_SC_iPSC/analysis`

General Statistics

Copy table | Configure Columns | Plot | Showing 9/9 rows and 14/19 columns.

Sample Name	M Reads Mapped	% rRNA	% mRNA	% Dups	% Aligned	M Aligned	% Aligned	% Duplication	GC content	% PF	%
H17_iPSC_brain											
H17_iPSC_spinal_cord											
H17_primary											
H18_iPSC_brain											
H18_iPSC_spinal_cord											
H18_primary											
H25_iPSC_brain											
H25_iPSC_spinal_cord											
H25_primary											

Picard

Picard (http://broadinstitute.github.io/picard/) is a set of Java command line tools for manipulating high-throughput sequencing data.

Mark Duplicates

Help

Number of reads, categorised by duplication state. **Pair counts are doubled** - see help text for details.

Number of Reads | Percentages

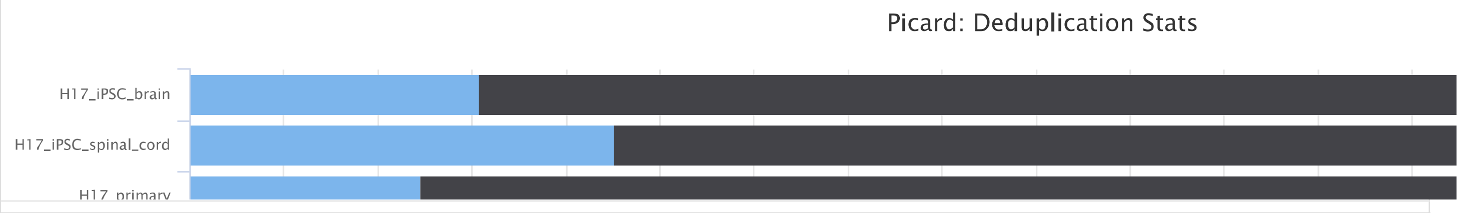

RnaSeqMetrics Assignment

Number of bases in primary alignments that align to regions in the reference genome.

Counts | Percentages

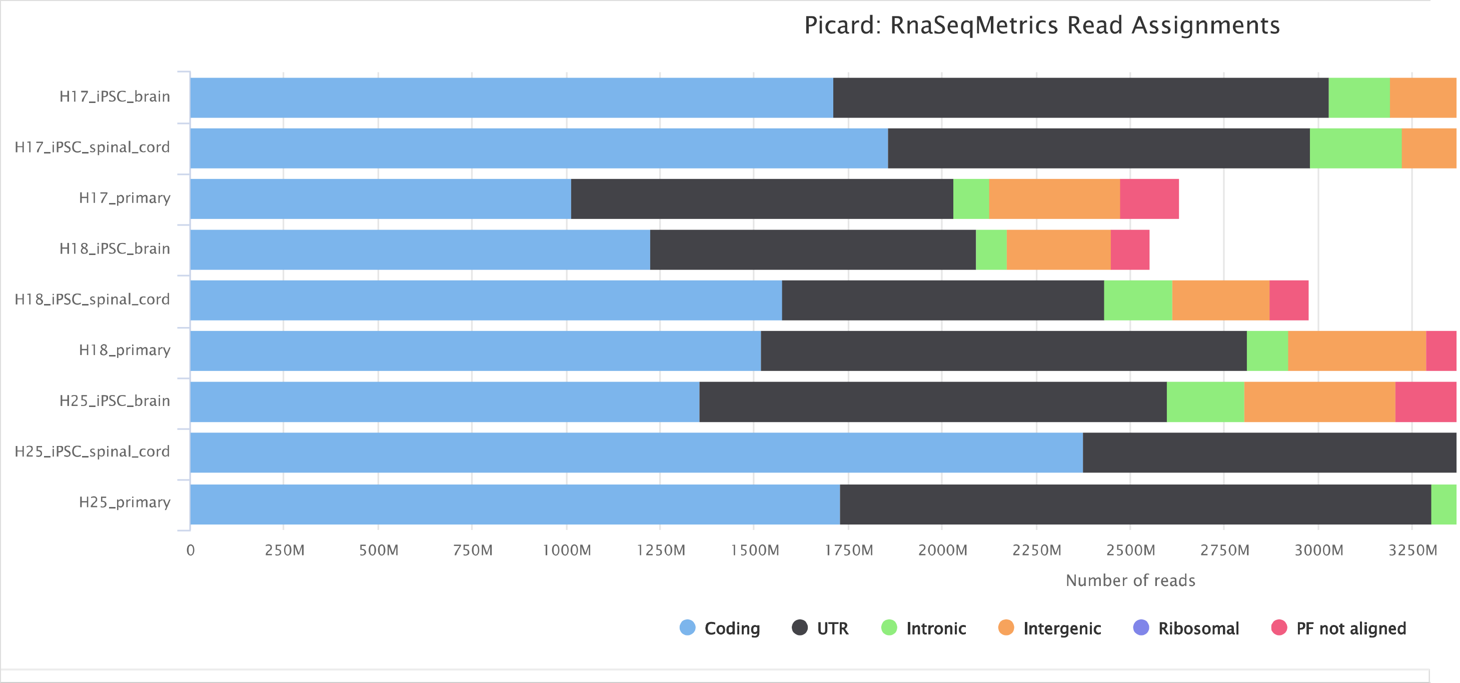

RnaSeqMetrics Strand Mapping

Number of aligned reads that map to the correct strand.

Counts | Percentages

Gene Coverage

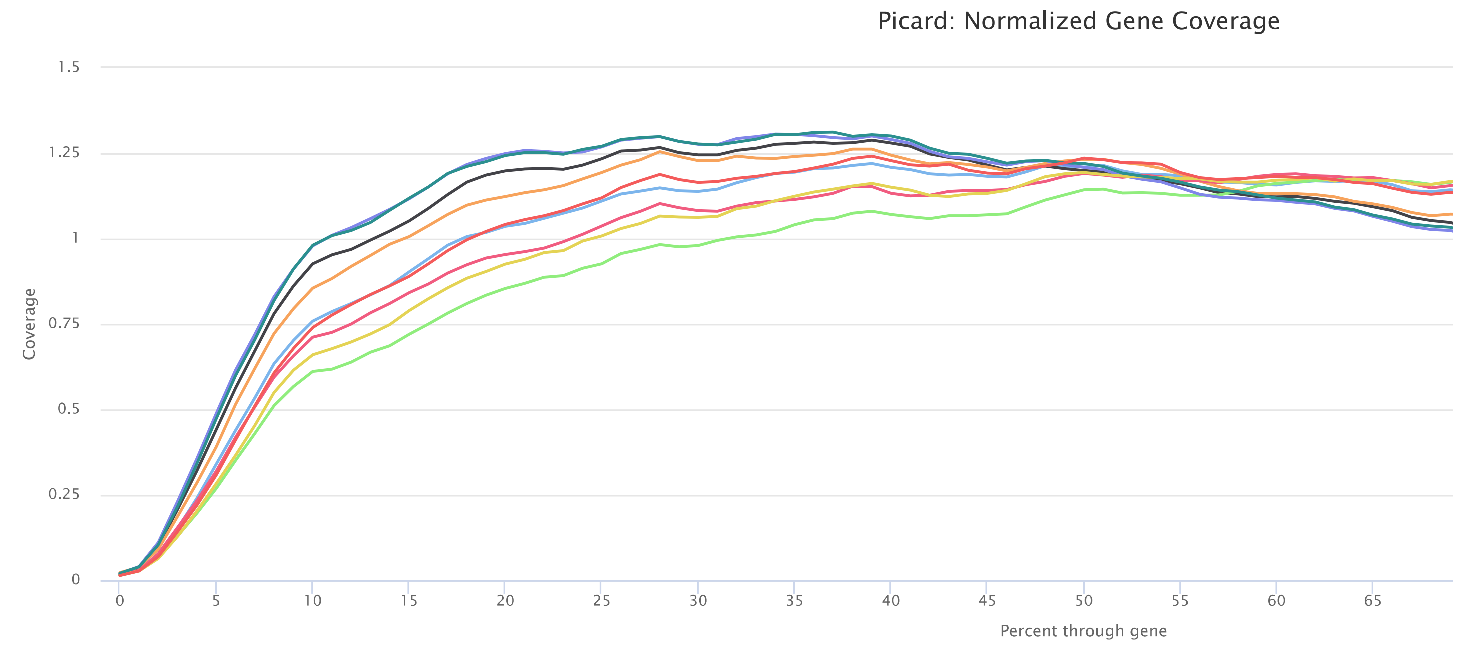

Samtools

Samtools (http://www.htslib.org) is a suite of programs for interacting with high-throughput sequencing data.

Samtools Flagstat

This module parses the output from `samtools flagstat`. All numbers in millions.

Hover over a data point for more information

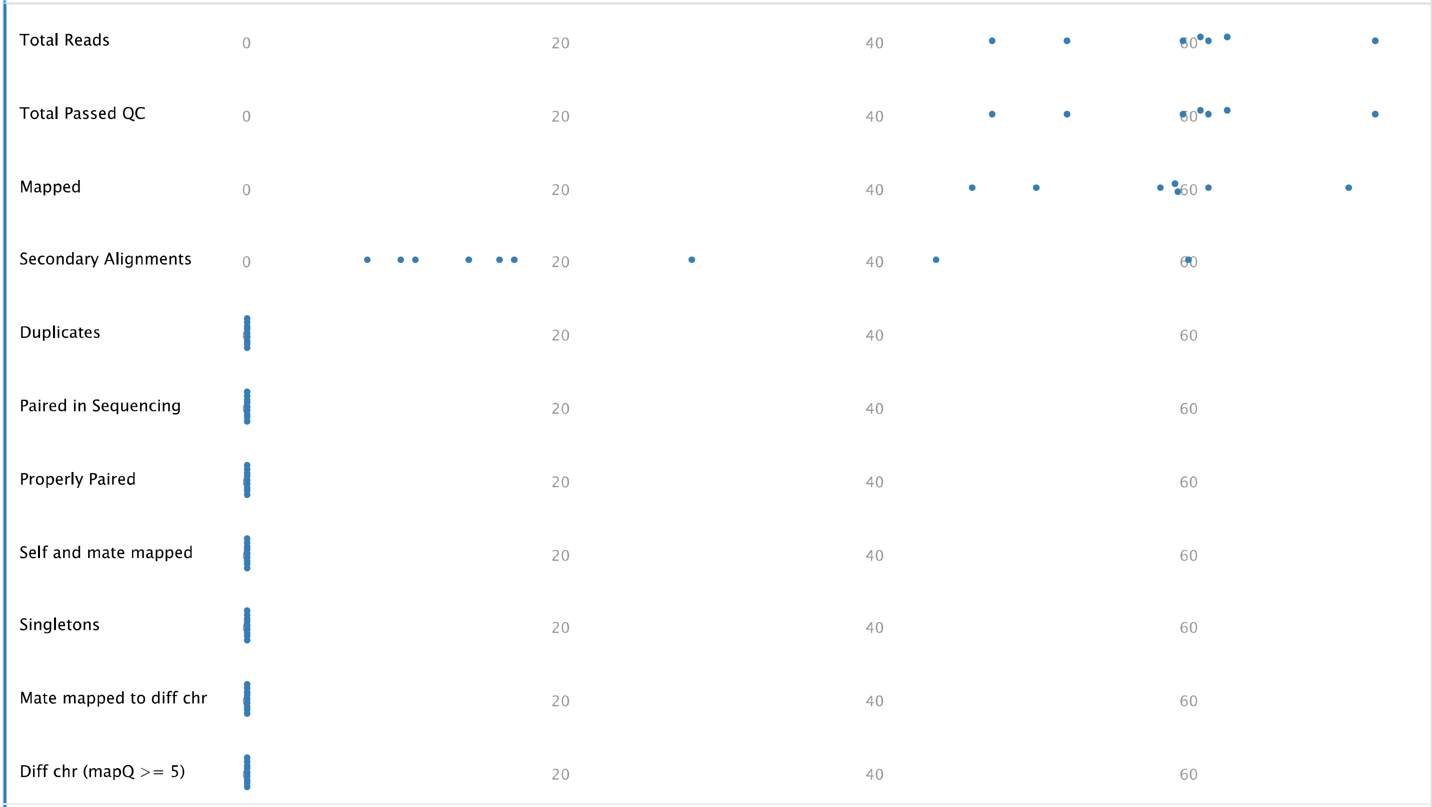

XY counts

Number of Reads | Percent of X+Y Reads

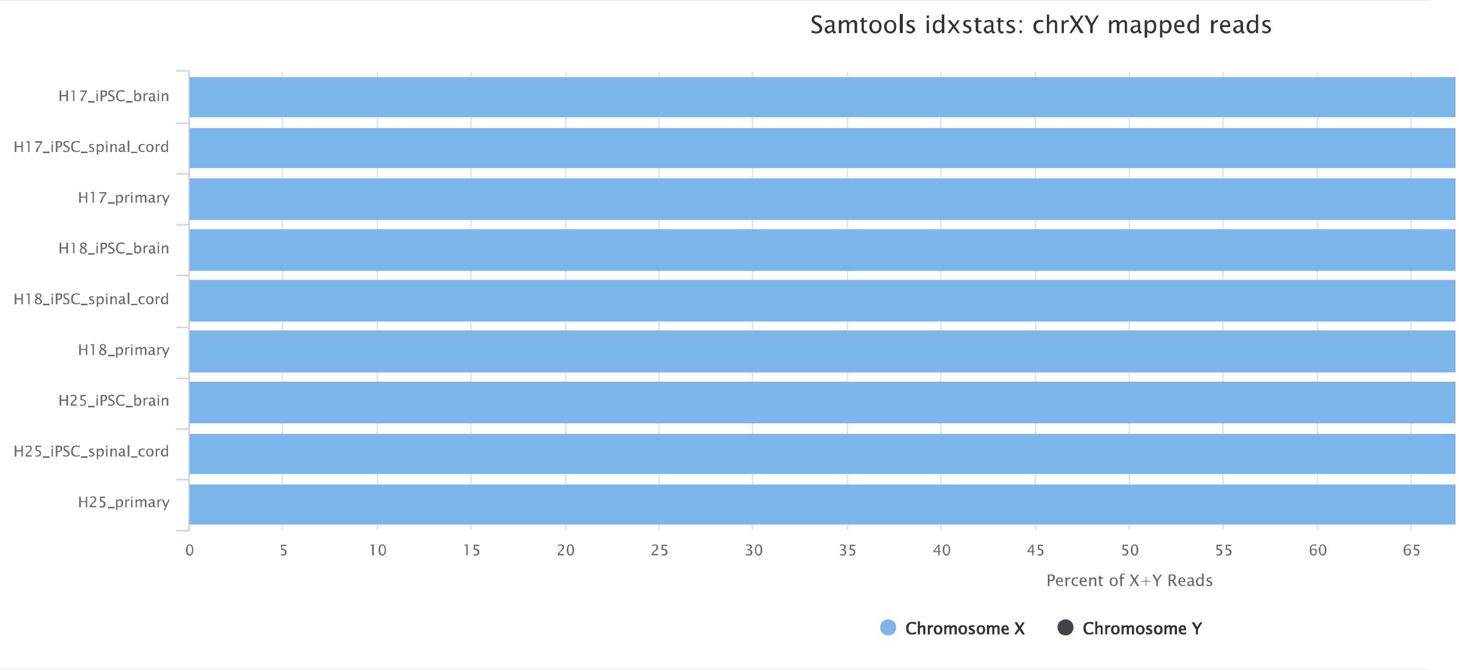

Mapped reads per contig

The `samtools idxstats` tool counts the number of mapped reads per chromosome / contig. Chromosomes with < 0.1% of the total aligned reads are omitted from this plot.

Counts | Log10 | Normalised Counts | Observed over Expected Counts | Raw Counts

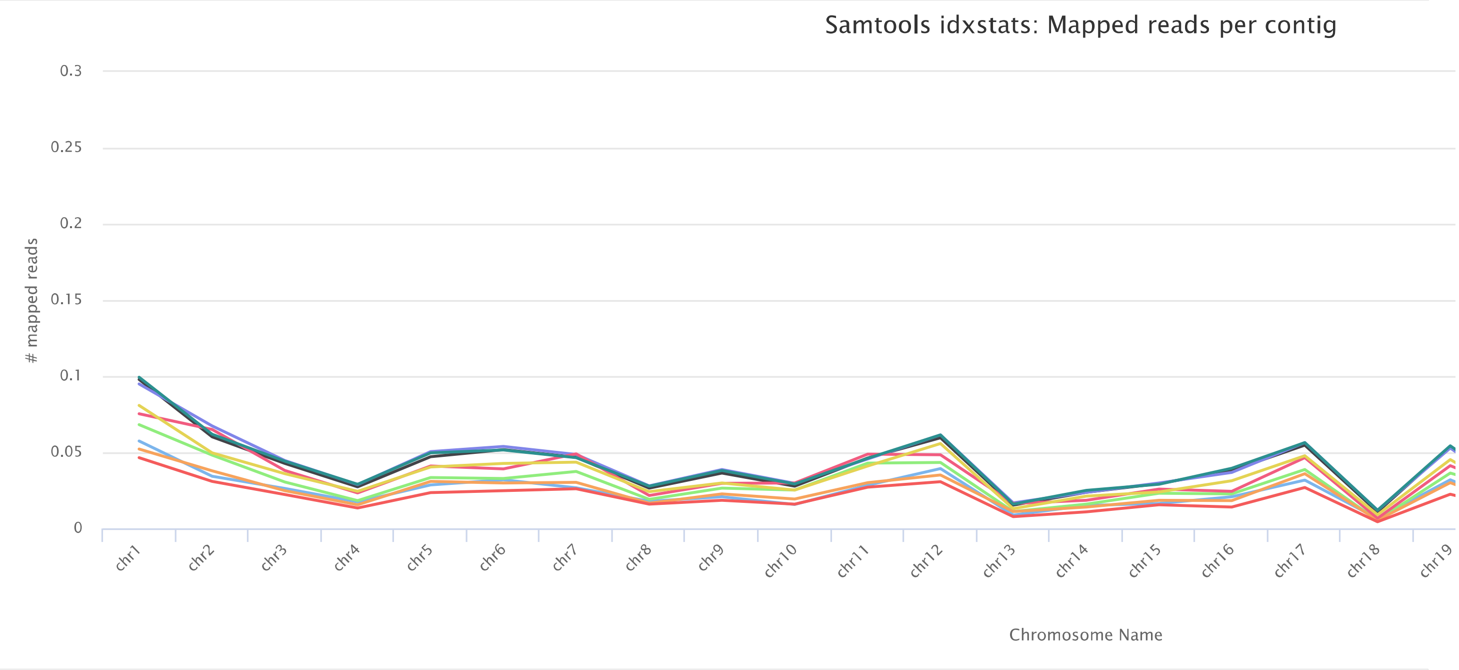

Salmon

Salmon (http://combine-lab.github.io/salmon/) is a tool for quantifying the expression of transcripts using RNA-seq data.

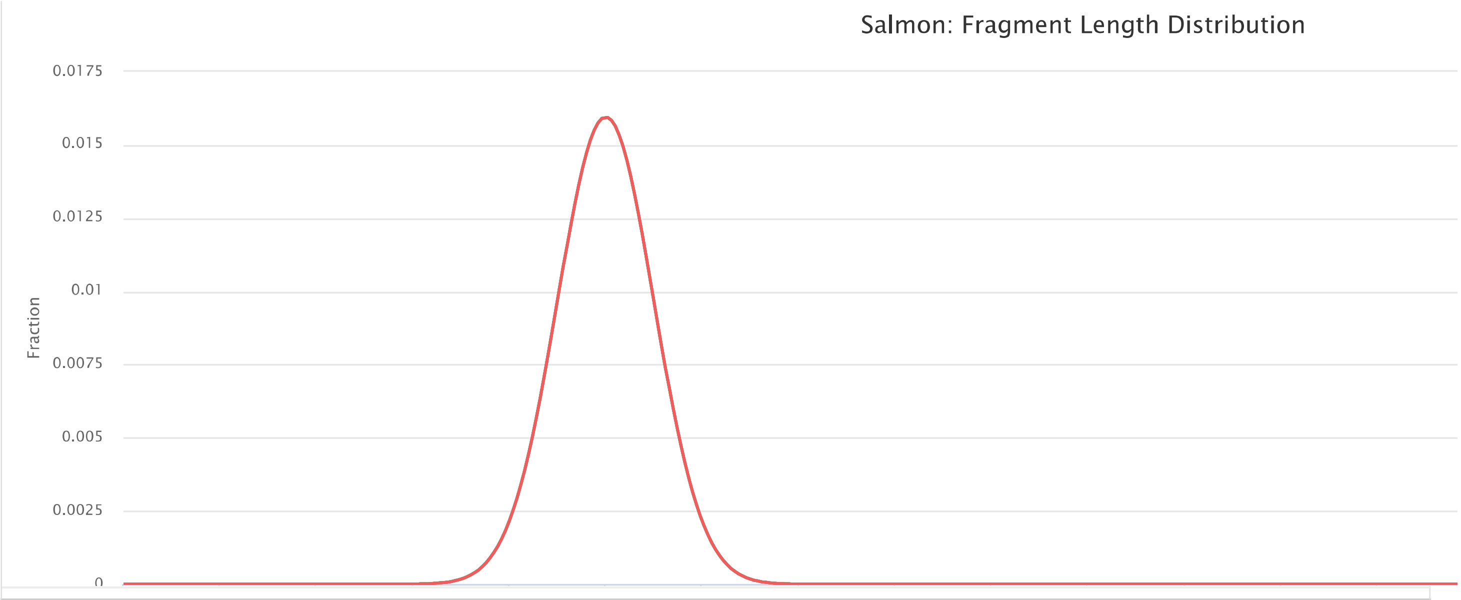

HISAT2

HISAT2 (https://ccb.jhu.edu/software/hisat2/) is a fast and sensitive alignment program for mapping NGS reads (both DNA and RNA) against a reference genome or population of reference genomes.

Number of Reads | Percentages

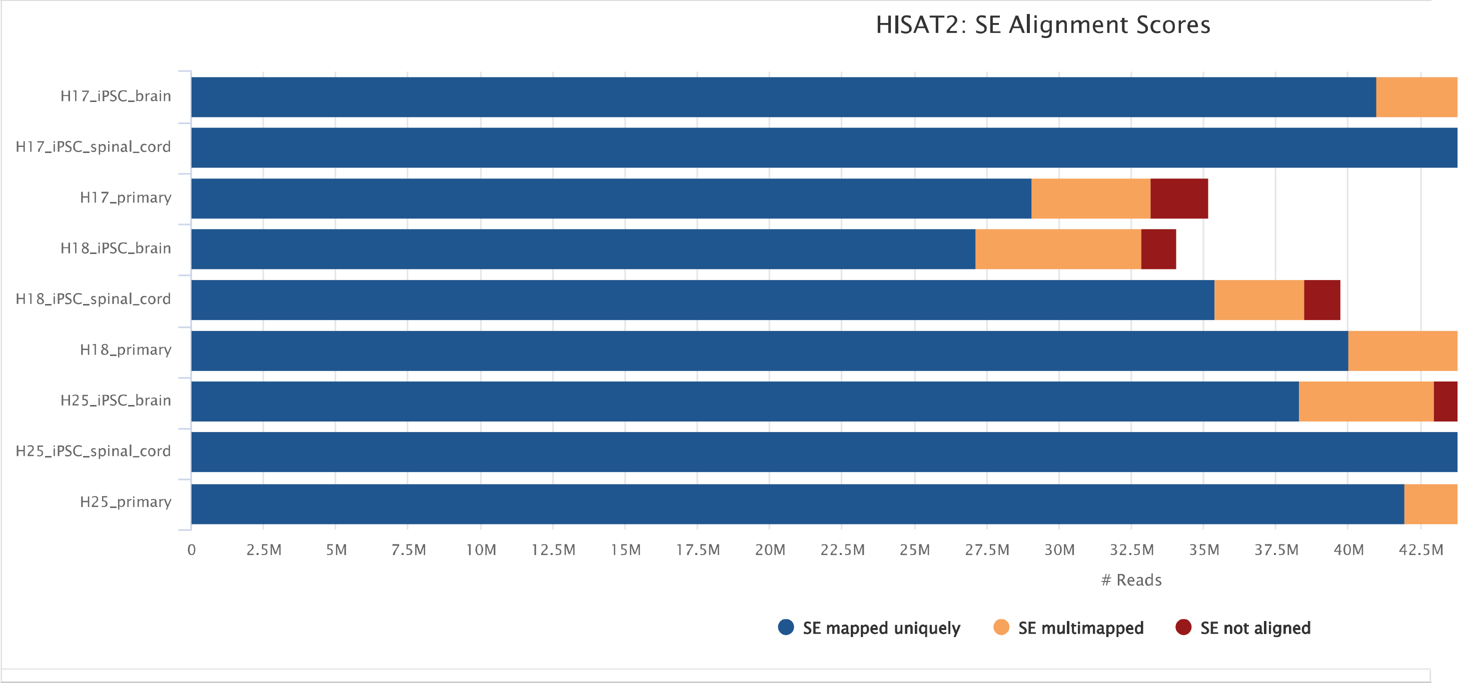

fastp

fastp (https://github.com/OpenGene/fastp) An ultra-fast all-in-one FASTQ preprocessor (QC, adapters, trimming, filtering, splitting...)

Filtered Reads

Filtering statistics of sampled reads.

Number of Reads | Percentages

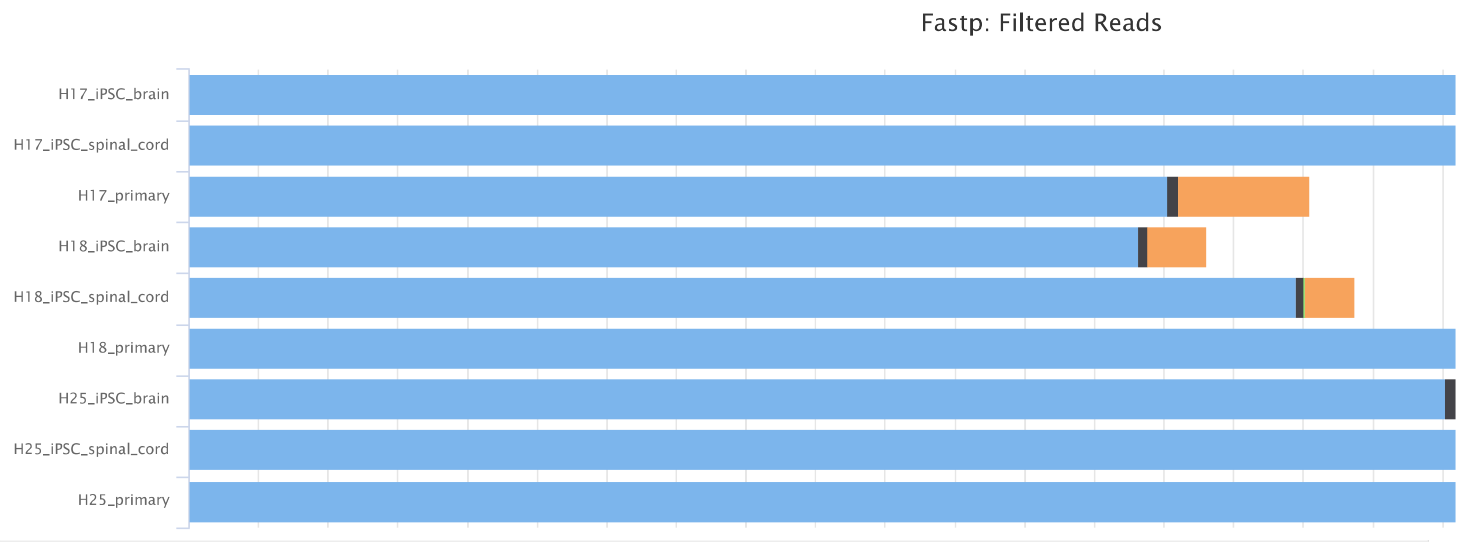

Duplication Rates

Duplication rates of sampled reads.

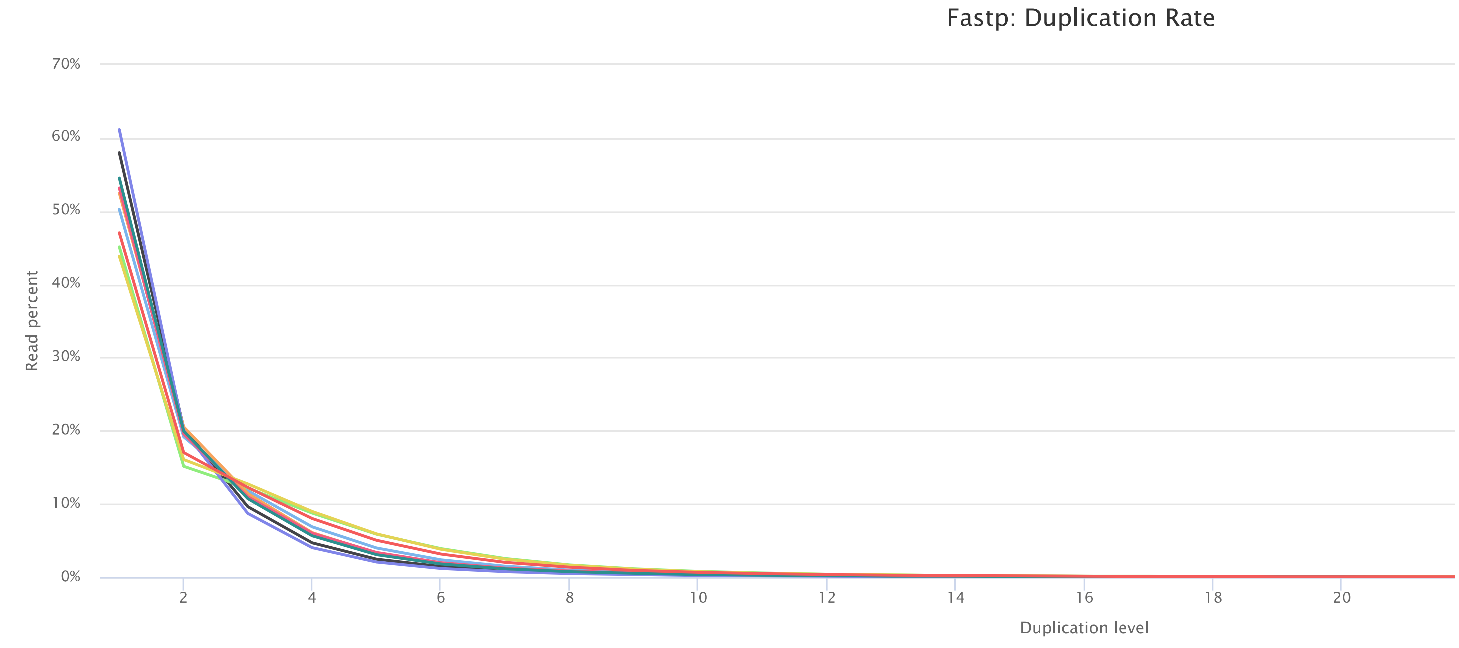

Sequence Quality

Average sequencing quality over each base of all reads.

Read 1: Before filtering | Read 1: After filtering

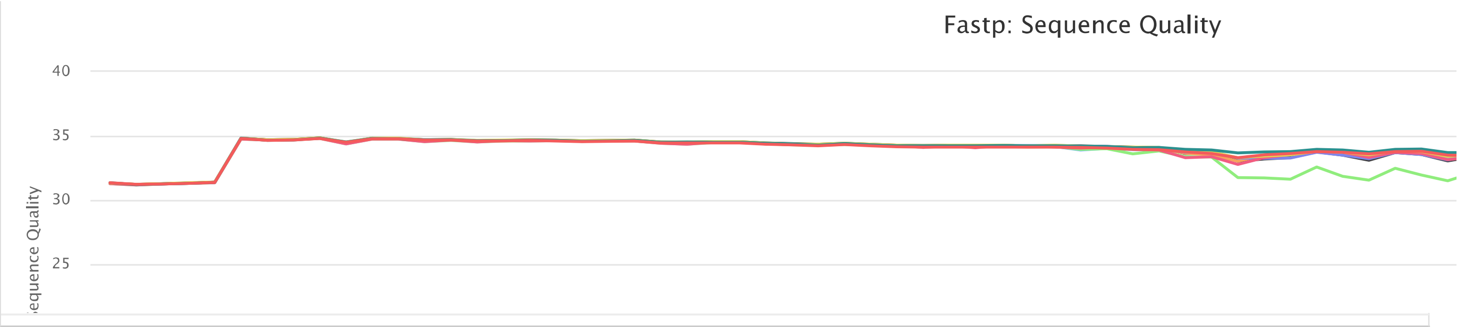

GC Content

Average GC content over each base of all reads.

Read 1: Before filtering | Read 1: After filtering

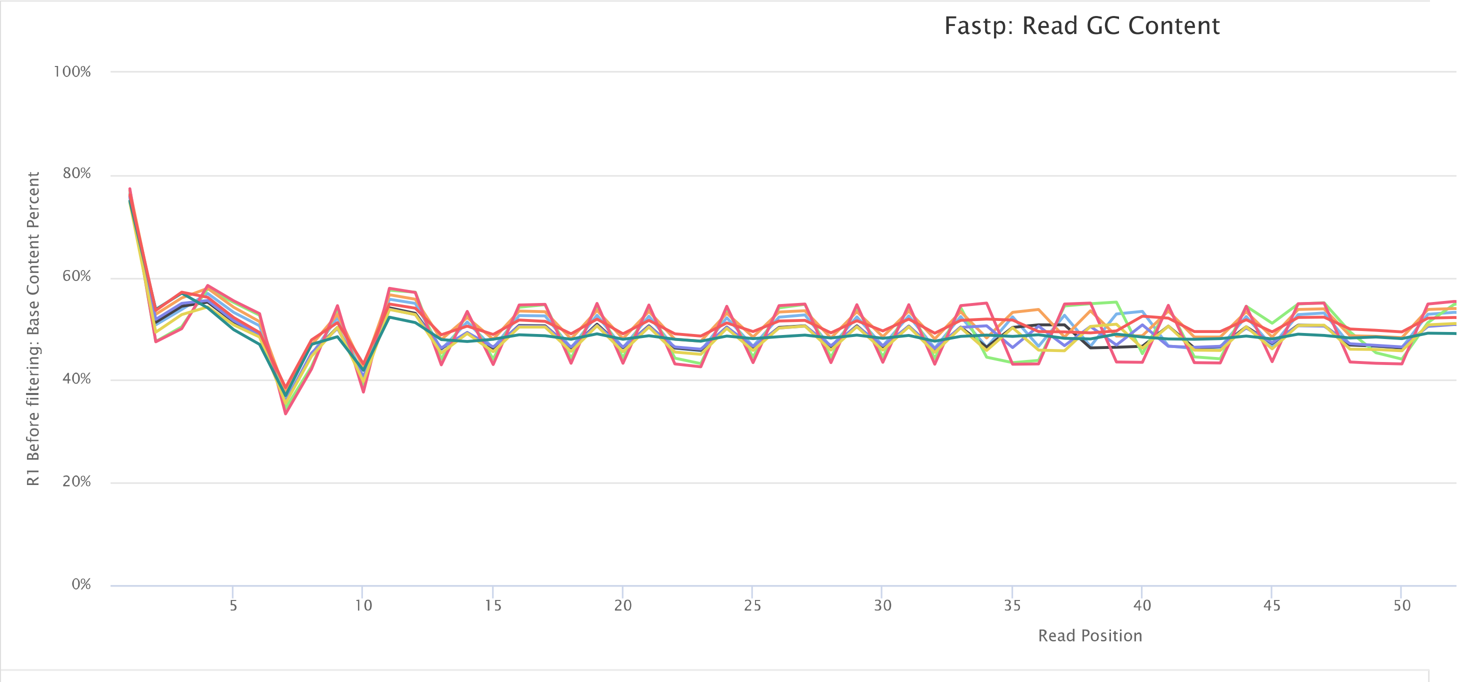

N content

Average N content over each base of all reads.

Read 1: Before filtering | Read 1: After filtering

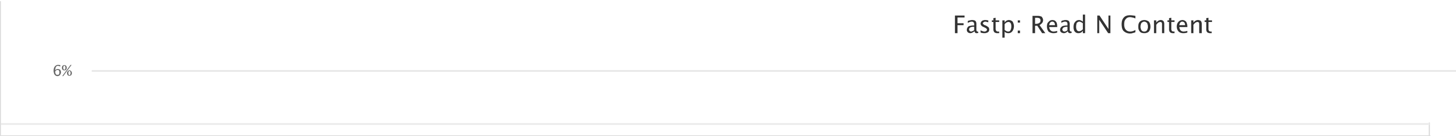

FastQC

FastQC (http://www.bioinformatics.babraham.ac.uk/projects/fastqc/) is a quality control tool for high throughput sequence data, written by Simon Andrews at the Babraham Institute in Cambridge.

Sequence Counts

Help

Sequence counts for each sample. Duplicate read counts are an estimate only.

Number of reads | Percentages

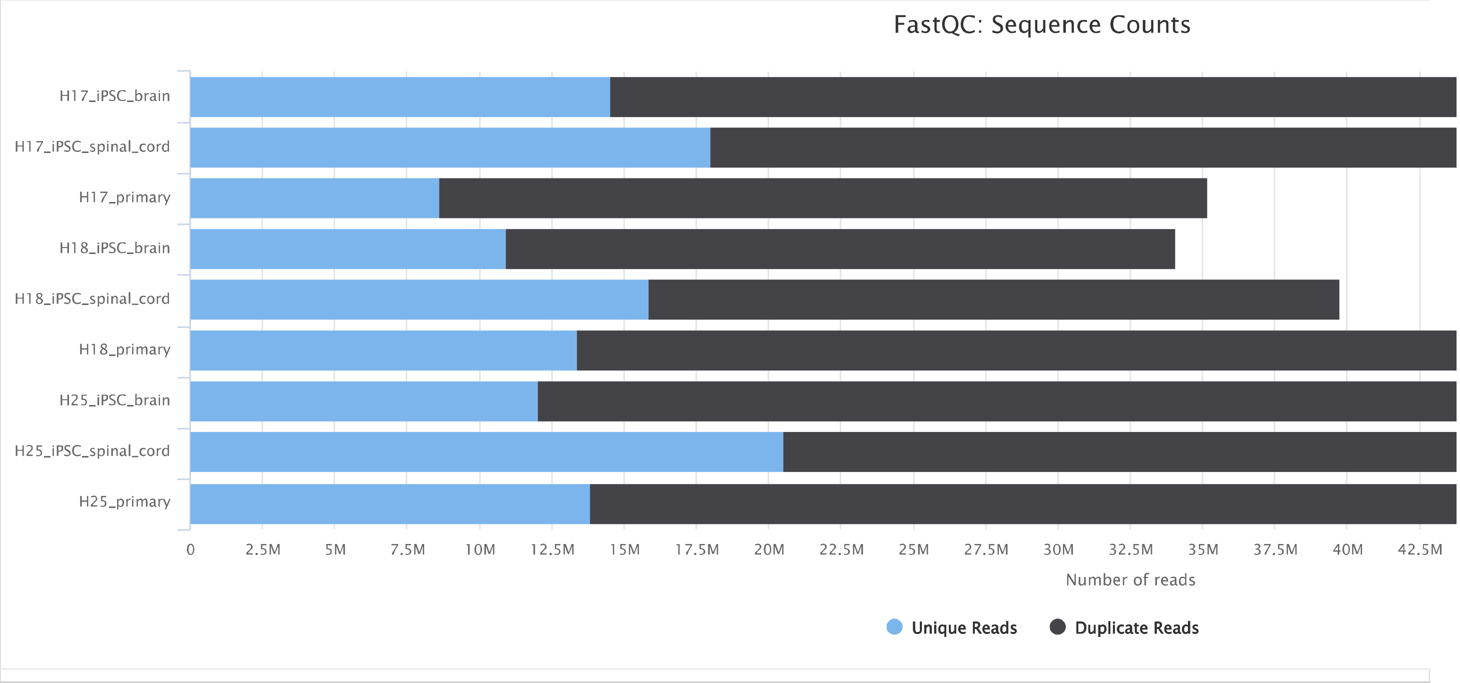

Sequence Quality Histograms

9

Help

The mean quality value across each base position in the read.

FastQC: Mean Quality Scores

Per Sequence Quality Scores 9

Help

The number of reads with average quality scores. Shows if a subset of reads has poor quality.

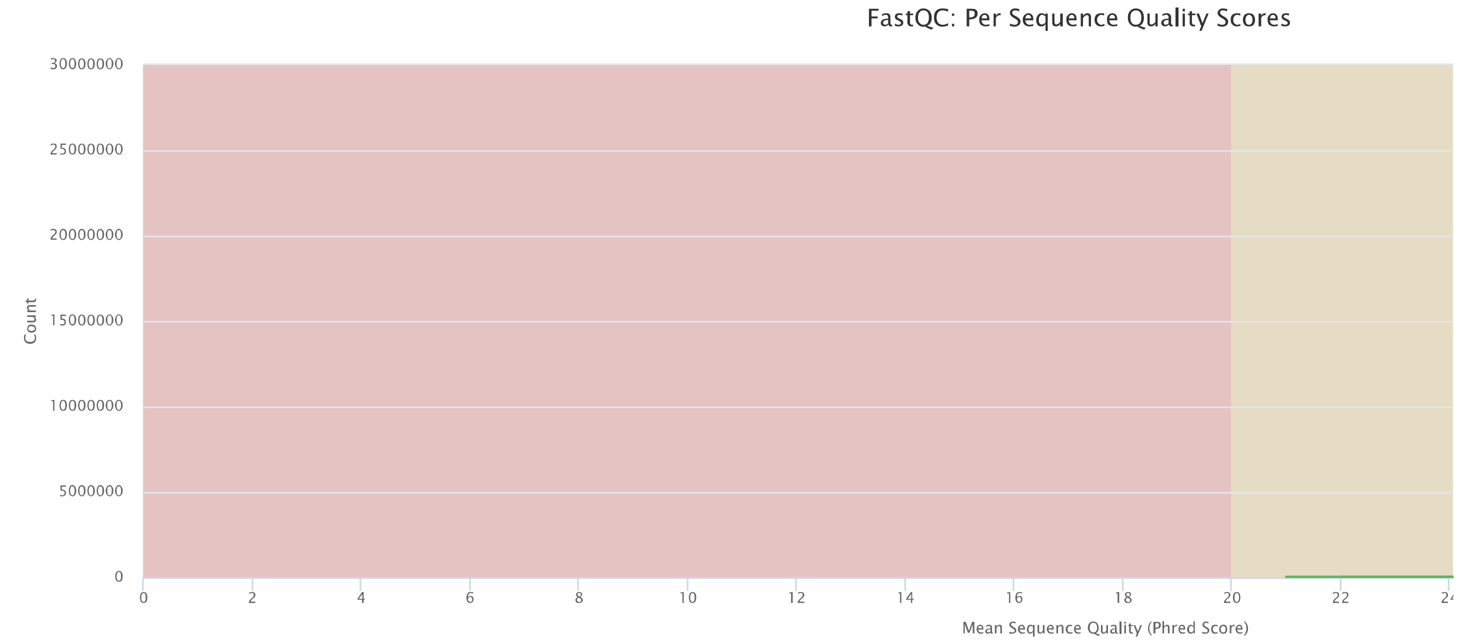

Per Base Sequence Content 0 6 3

Help

The proportion of each base position for which each of the four normal DNA bases has been called.

Click a sample row to see a line plot for that dataset.

Rollover for sample name

Position: - %T: - %C: - %A: - %G: -

Export Plot

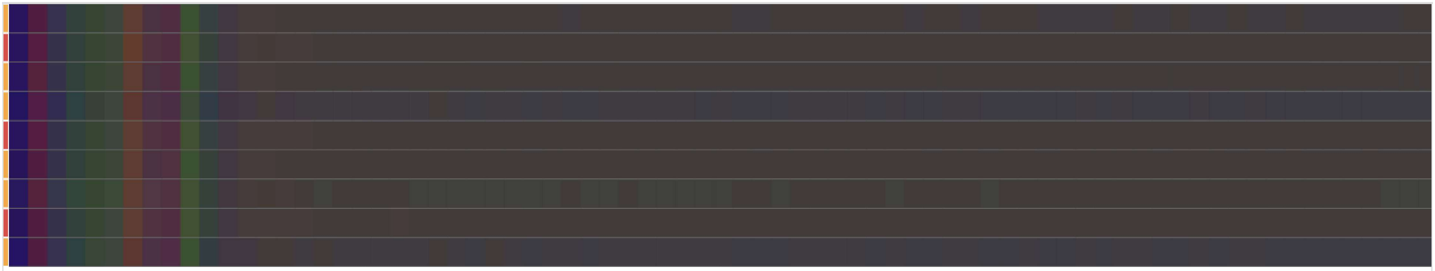

Per Sequence GC Content 9

Help

The average GC content of reads. Normal random library typically have a roughly normal distribution of GC content.

Percentages Counts

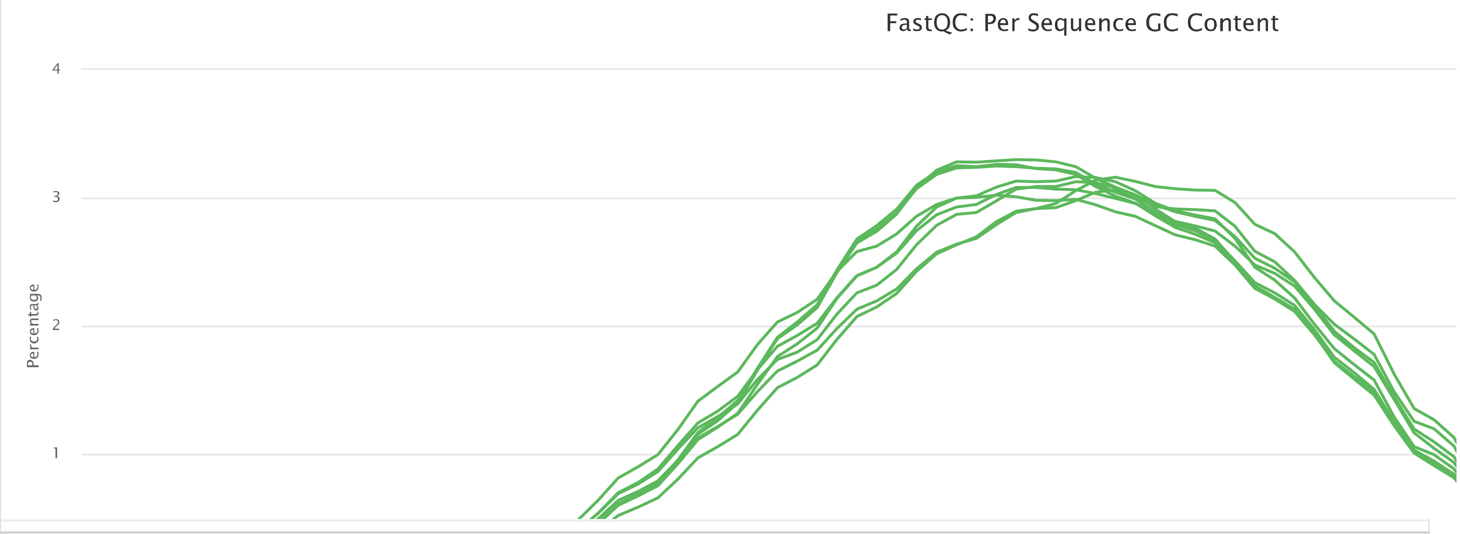

Per Base N Content

9

Help

The percentage of base calls at each position for which an `N` was called.

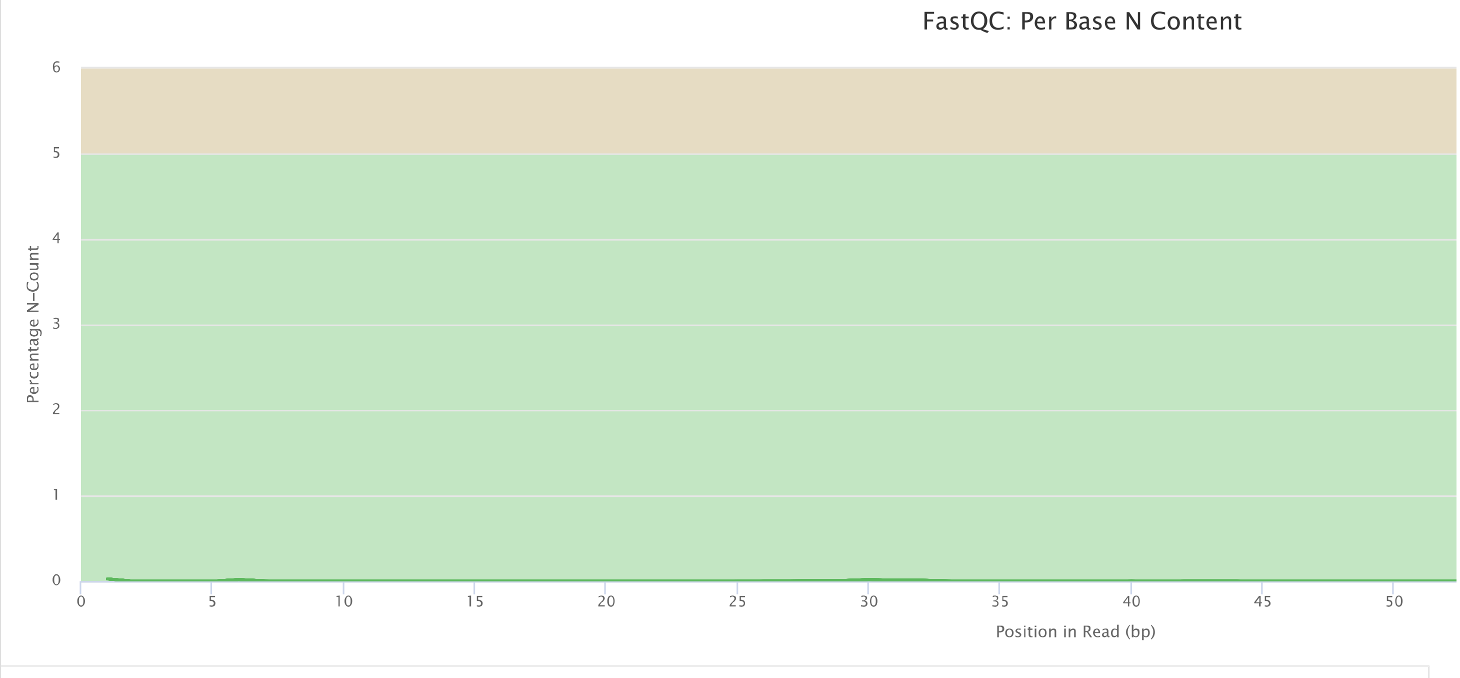

Sequence Length Distribution

0 9

The distribution of fragment sizes (read lengths) found. See the FastQC help (http://www.bioinformatics.babraham.ac.uk/projects/fastqc/Help/3%20Analysis%20Modules/7%20Sequence%20Length%20Distribution.html)

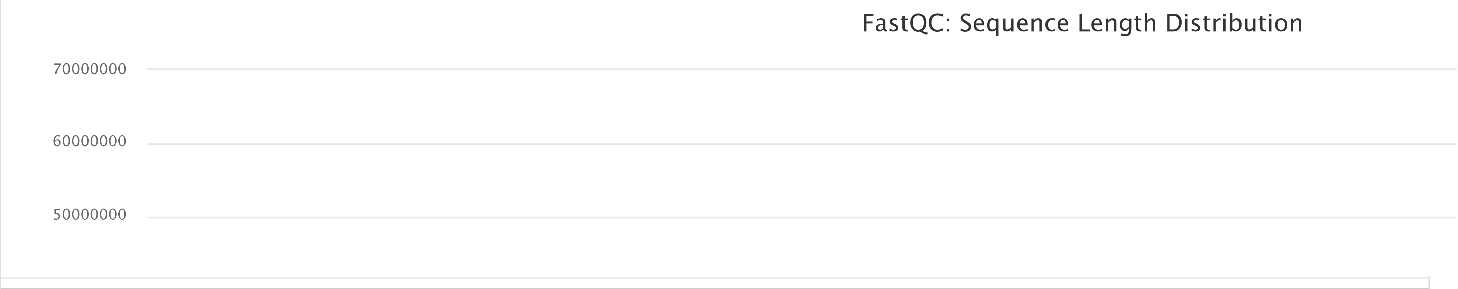

Sequence Duplication Levels 0 9

Help

The relative level of duplication found for every sequence.

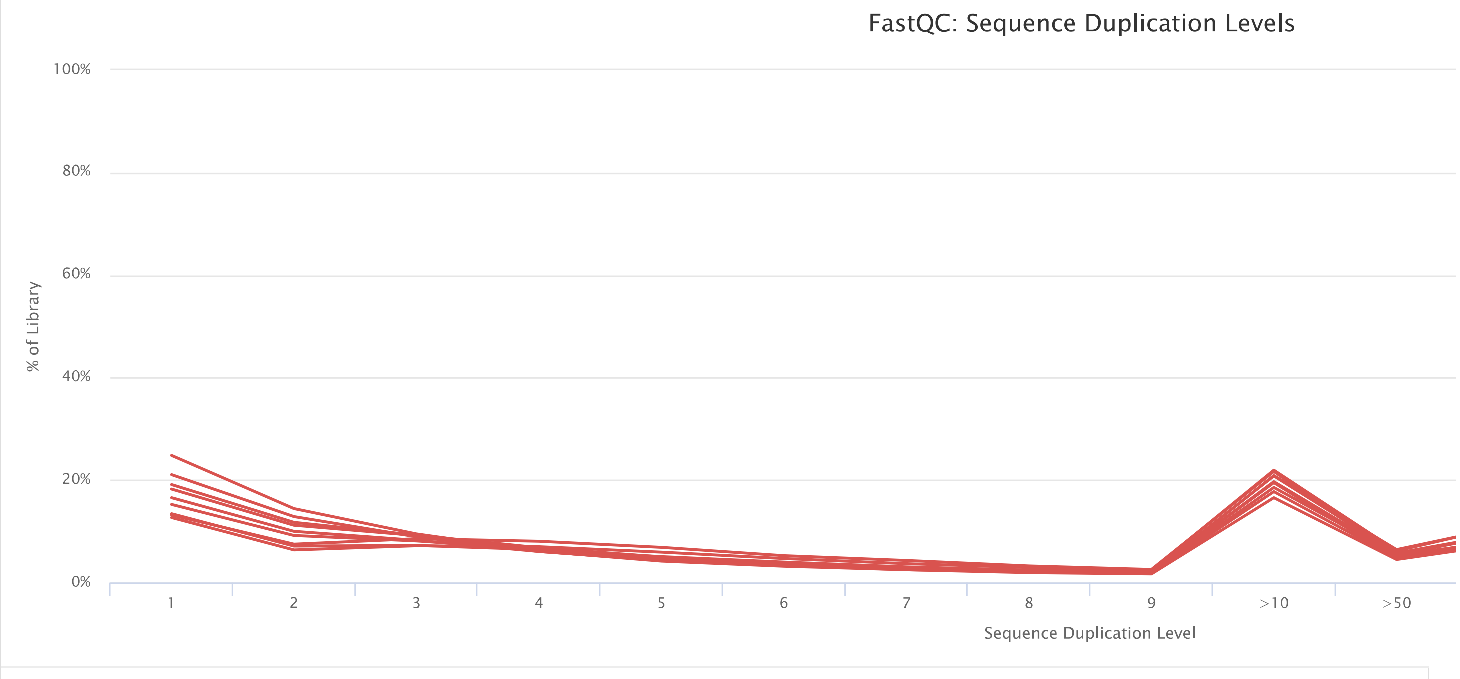

Overrepresented sequences 9

Help

The total amount of overrepresented sequences found in each library.

9 samples had less than 1% of reads made up of overrepresented sequences

Adapter Content 9

Help

The cumulative percentage count of the proportion of your library which has seen each of the adapter sequences at each position.

No samples found with any adapter contamination > 0.1%

Status Checks

Help

Status for each FastQC section showing whether results seem entirely normal (green), slightly abnormal (orange) or very unusual (red).

Sort by highlight

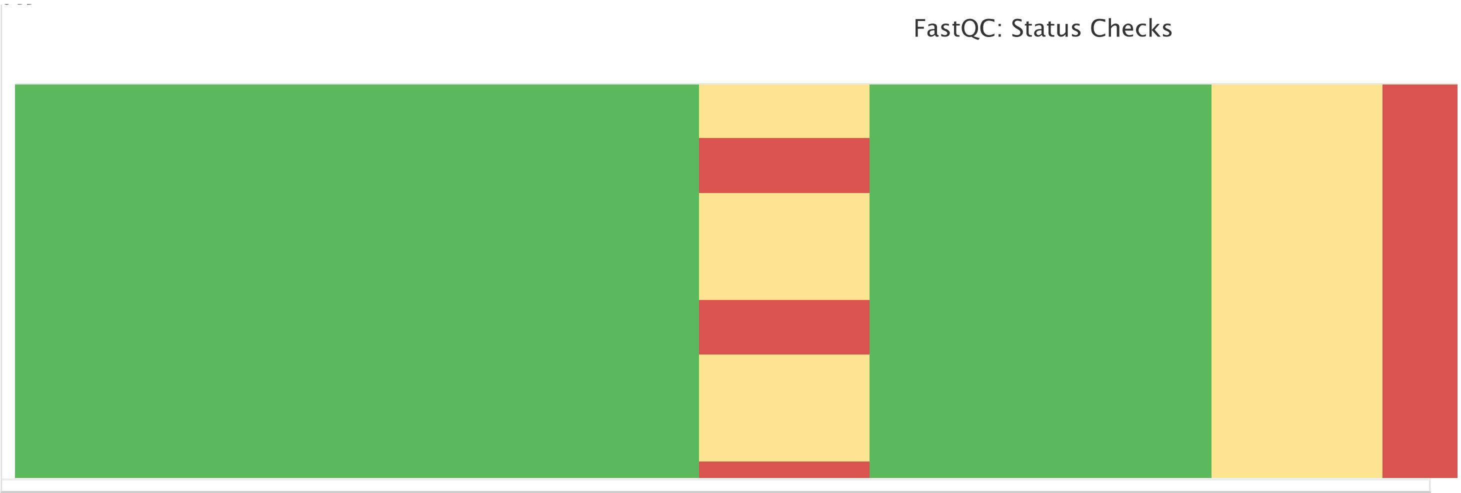

MultiQC v1.10.1 (http://multiqc.info) - Written by Phil Ewels (http://phil.ewels.co.uk), available on GitHub (https://github.com/ewels/MultiQC).
This report uses HighCharts (http://www.highcharts.com/), jQuery (https://jquery.com/), jQuery UI (https://jqueryui.com/), Bootstrap (http://getbootstrap.com/), FileSaver.js (https://github.com/eligrey/FileSaver.js) and clipboard.js (https://clipboardjs.com/).

SciLifeLab (http://www.scilifelab.se/)

RNA-seq analysis - human spinal cord SCs and iPSCs

Christopher Porter, Ottawa Bioinformatics Core Facility (OBCF)

June 30, 2022

- QC metrics
- Transcript Quantification
- Run DESeq2
  - Retrieve gene annotation data
  - Read counts and FPKM values
- Diagnostic plots
  - Principal Component Analysis (PCA)
  - Hierarchical clustering
  - Significantly DE genes
  - Volcano plots
- Compare Results
  - Fold difference in iPSC-Brain and iPSC-SC vs primary SCSC
  - Genes subsets significant in both iPSC_brain and iPSC_SC, or only one.
- Heatmap of significantly DE genes
  - Genes significantly DE in iPSC_brain vs primary_SCSC
  - Genes significantly DE in iPSC_SC vs primary_SCSC
- Comparison to Ependymal cell DE gene list from single cell data.
- Other markers of interest
  - Spinal Cord
  - Brain genes
  - Msoderm genes
  - NSC genes
  - Ependymal genes
  - Proliferation genes
  - Neurogenesis genes
  - Astrogenesis genes
  - Oligodendrogensis genes
  - Dorsal genes
  - Ventral genes
- More heatmaps with MSigDb gene sets
  - REACTOME_INTERFERON_ALPHA_BETA_SIGNALING genes
  - REACTOME_INTERFERON_GAMMA_SIGNALING genes
- File generation date and environment
- Appendix: Dispersion plots
- Appendix: MA plots
- Appendix: Count scatter plots

Analysis prepared for Dr Eve Tsai and Ahmad Galuta

This document reports on analysis of RNA-seq data from:

- Human spinal cord stem cells (6 replicates)
- Human iPSCs differentiated as spinal cord stem cells (3 replicates)
- Human iPSCs differentiated as brain cord stem cells (3 replicates)

I applied the most recent version of our RNA-seq pipeline to these data. Reads are assigned to transcripts using Salmon (https://combine-lab.github.io/salmon/ (https://combine-lab.github.io/salmon/)) and the transcript quantifications generated are loaded into R for analysis with DESeq2.

QC metrics

I ran QC on the samples using FastQC to assess the quality of the raw sequences, and Picard RNA-seq metrics to assess the mapped reads (mapped to the mouse transcriptome using hisat2). A summary report of QC metrics and mapping statistics can be retrieved by clicking this link (http://www.ogic.ca/projects/ahmad_galuta/human_spinal_cord_SC_iPSC/reports/multiqc_report.html).

Transcript Quantification

The summary of read assignment to transcripts from GENCODE release 35 is found in the table below.

Read assignment to transcripts

	Reads processed	Reads mapped	Percent mapped
H17_iPSC_brain	52,051,702	42,369,562	81.39899
H17_iPSC_spinal_cord	50,951,658	45,914,716	90.11427
H17_primary	35,157,569	29,648,130	84.32929
H18_iPSC_brain	34,091,203	28,075,471	82.35400

	Reads processed	Reads mapped	Percent mapped
H18_iPSC_spinal_cord	39,733,215	35,911,384	90.38127
H18_primary	45,505,648	41,138,552	90.40318
H25_iPSC_brain	45,095,052	38,200,701	84.71151
H25_iPSC_spinal_cord	61,142,321	55,261,751	90.38216
H25_primary	55,438,283	43,417,877	78.31750
H35	51,302,883	42,713,311	83.25714
H38	47,573,072	40,771,932	85.70380
H39	40,820,262	35,871,542	87.87680

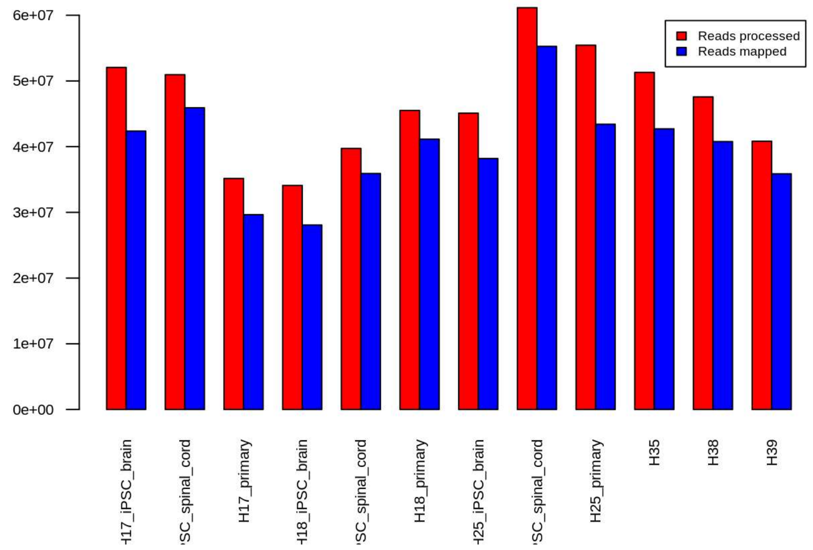

Rates of read assignment to transcripts are good (all greater than 78.32%).

Run DESeq2

The next analysis steps comprise:

- Import transcript abundances from salmon using the `tximport` library.
- Filter to retain only genes with 5 or more counts in two or more samples.

A total of 60,237 rows (genes) were imported by `tximport` ; after filtering, 24,006 were retained for further analysis. The following steps perform these actions:

- Annotate samples (see/check Sample data table below)
- Run DESeq2
    - estimate size factors (scaling for library sequencing depth)
    - estimate dispersions
    - estimate gene-wise dispersions
    - calculate mean-dispersion relationship
    - calculate final dispersion estimates
    - fit model and test
- Experimental design is `~patient + cell_type` .
- Calculate rlog-transformed normalized counts for PCA and hierarchical clustering.

Sample annotations in DESeq2

sampleName	cell_type	patient
H17_iPSC_brain	iPSC_brain	H17
H17_iPSC_spinal_cord	iPSC_SC	H17
H17_primary	primary_SCSC	H17
H18_iPSC_brain	iPSC_brain	H18
H18_iPSC_spinal_cord	iPSC_SC	H18
H18_primary	primary_SCSC	H18
H25_iPSC_brain	iPSC_brain	H25
H25_iPSC_spinal_cord	iPSC_SC	H25
H25_primary	primary_SCSC	H25

sampleName	cell_type	patient
H35	primary_SCSC	H35
H38	primary_SCSC	H38
H39	primary_SCSC	H39

Retrieve gene annotation data

Annotations were found in the Biomart data for all put 48 of the 24,006 genes in the full results set.

Read counts and FPKM values

I exported the raw read counts and FPKM values as tab-delimited text files, which can be downloaded using these links.

- File of raw read counts per gene (http://ogic.ca/projects/ahmad_galuta/human_spinal_cord_SC_iPSC/results/human_SC_iPSC_read_count_matrix.txt)
- File of normalized read counts per gene (http://ogic.ca/projects/ahmad_galuta/human_spinal_cord_SC_iPSC/results/human_SC_iPSC_normalized_read_count_matrix.txt)
- File of FPKM values per gene (http://ogic.ca/projects/ahmad_galuta/human_spinal_cord_SC_iPSC/results/human_SC_iPSC_fpkm_matrix.txt)

Diagnostic plots

Running PCA and hierarchical clustering allow us to visualize the similarity of the different samples/replicates.

Principal Component Analysis (PCA)

Applying principal component analysis (PCA) to the matrix of gene expression values (read counts) identifies the major components of gene expression variation. Plotting the principal component values for each sample gives an overview of the relationship between the samples. The implementation in DESeq by default uses the 500 most variable genes in the dataset to generate the PCA.

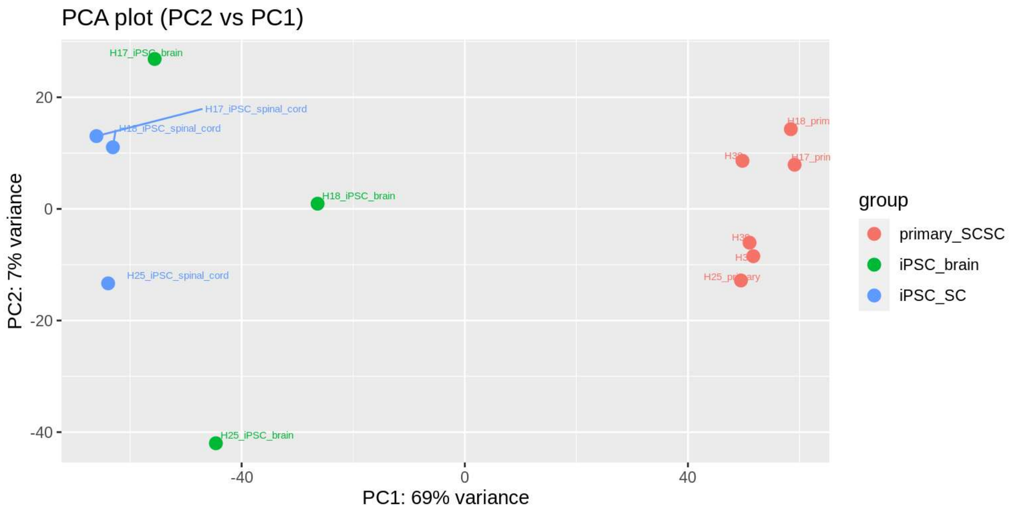

Plotting the first two principal components separates the primary SC stem cells from the iPSC samples along PC1 (the greatest axis of variation). PC2 does not separate the three sets of cells from each other, but it may be reflecting some element of the biological variation between patients. Within each cell type the H17 and H18 samples have positive PC2 values, whilst H25 has negative values. It's reassuring that all six of the primary SC samples cluster together, with no obvious batch effects resulting from having been run on separate sequencing runs.

Next we'll take a look at PC3 vs PC1.

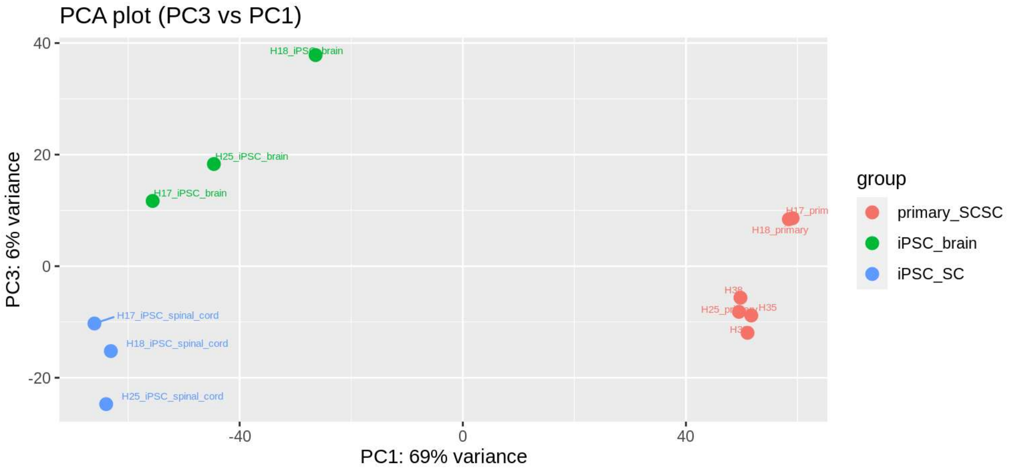

When we plot PC3 against PC1, the brain iPSCs are separated from the spinal cord iPSCs along the PC3 axis, and primaries are separated from iPSCs along PC1.

```
## NULL
```

Hierarchical clustering

Hierarchical clustering is calculated using Euclidian distance between rlog-transformed normalized count values for all transcripts,

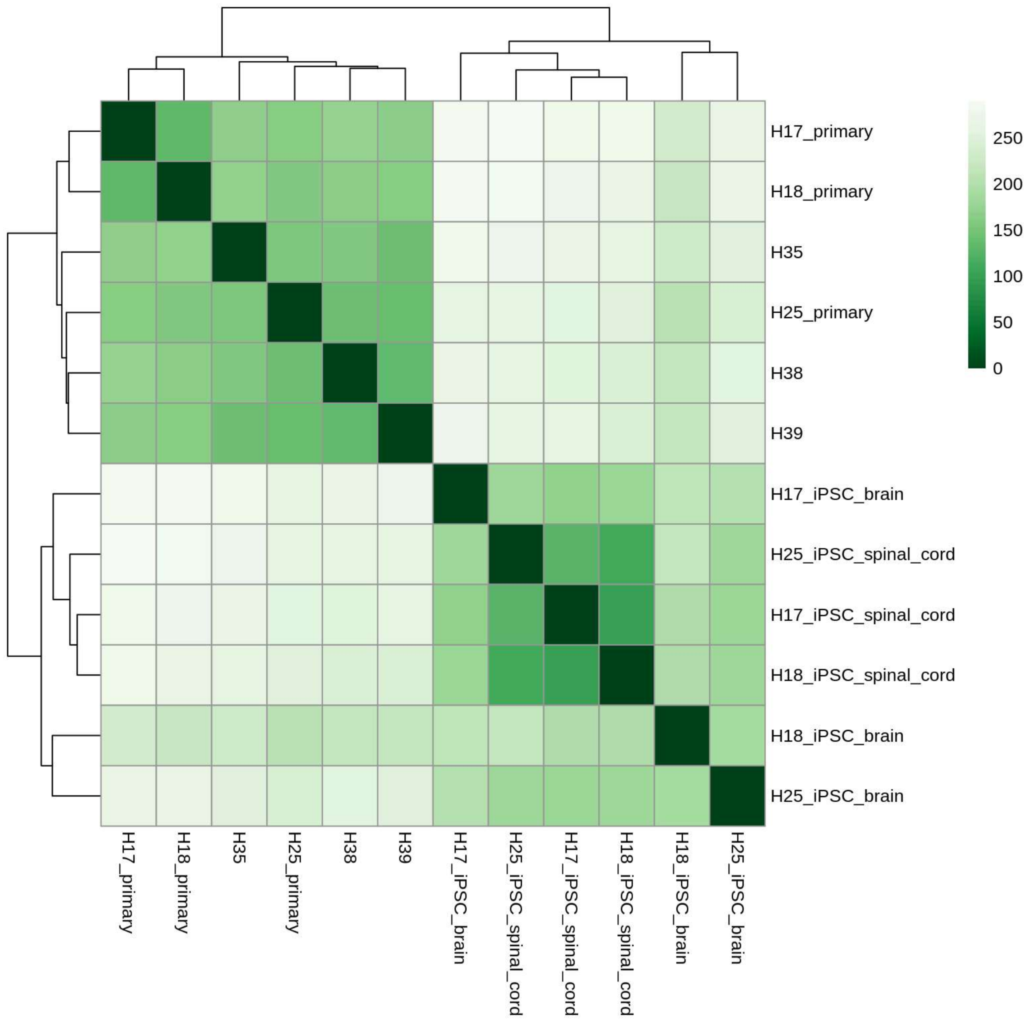

The hierarchical clustering analysis shows a pattern similar to that seen in the PCA. Primary SCs cluster separately from iPSCs, and within the iPSCs, the three spinal cord samples cluster closely together. As we saw in the first PCA plot, the brain iPSC samples appear more variable than the spinal cord iPSCs.

Significantly DE genes

I calculated fold changes between each pair of conditions. DESeq calculates fold change and a p-value for whether the fold change for that gene is significantly different from zero. It also performs multiple testing correction using the Benjamini-Hochberg method to calculate a q-value or FDR (at a q-value cut-off of 0.05 one would expect that 5% of the genes identified as differentially expressed would be false positives).

The number of genes that are identified as significantly different in expression at different FDR values is shown in the table below. There is no filtering for fold change or absolute expression value.

Differentially-expressed genes at multiple FDR values

	iPSC_brain_vs_primary_SCSC	iPSC_SC_vs_primary_SCSC	iPSC_brain_vs_iPSC_SC
padj<0.10	6,950	10,155	2,841
padj<0.05	5,555	8,737	2,015
padj<0.01	3,672	6,362	1,076
padj<0.002	2,633	4,918	645

Files of expression changes for all 24,006 genes, and for genes significantly different in expression (FDR < 5%) can be downloaded using the links in these tables.

Differential expression results

All genes	
iPSC_brain vs primary_SCSC all (24,006 genes). (http://www.ogic.ca/projects/ahmad_galuta/human_spinal_cord_SC_iPSC/results/iPSC_brain_vs_primary_SCSC_all_220630.txt)	(http://www.ogic.ca/projects/ahm
iPSC_SC vs primary_SCSC all (24,006 genes). (http://www.ogic.ca/projects/ahmad_galuta/human_spinal_cord_SC_iPSC/results/iPSC_SC_vs_primary_SCSC_all_220630.txt)	(http://www.ogic.ca/projects/al

All genes	
iPSC_brain vs iPSC_SC all (24,006 genes). (http://www.ogic.ca/projects/ahmad_galuta/human_spinal_cord_SC_iPSC/results/iPSC_brain_vs_iPSC_SC_all_220630.txt)	(http://www.ogic.ca/project

Volcano plots

Fold change results displayed as a volcano plot. The log2 fold change is plotted on the *x*-axis, and -log10(p-value) is plotted on the *y*-axis so that the most significant differences have higher *y*-axis values. When calculating fold changes, DESeq2 performs fold change shrinkage, which shrinks fold change estimates of a less confident magnitude towards zero. This can be seen in the fold changes close to zero around the line x=0.

iPSC_brain_vs_primary_SCSC

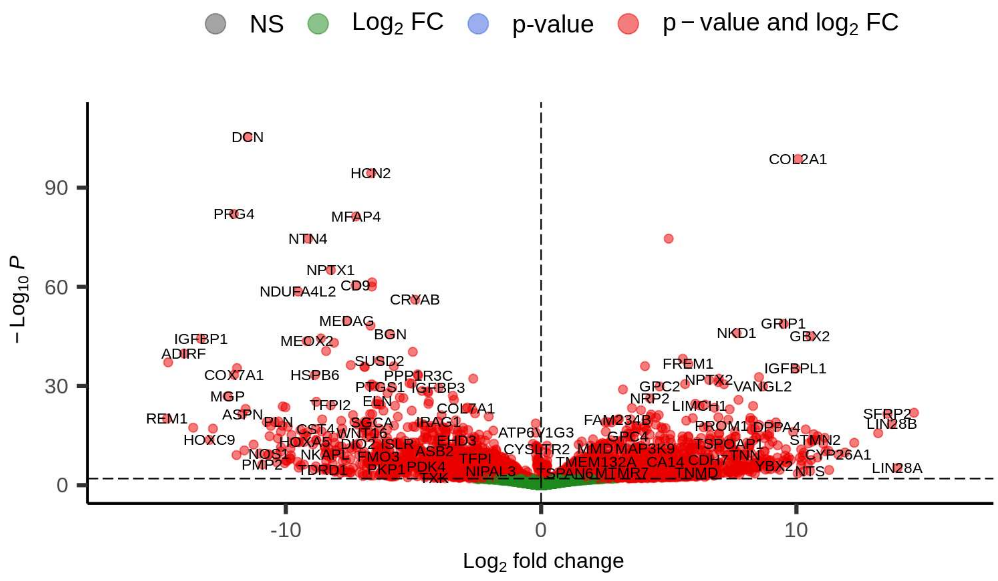

total = 24006 variables

iPSC_SC_vs_primary_SCSC

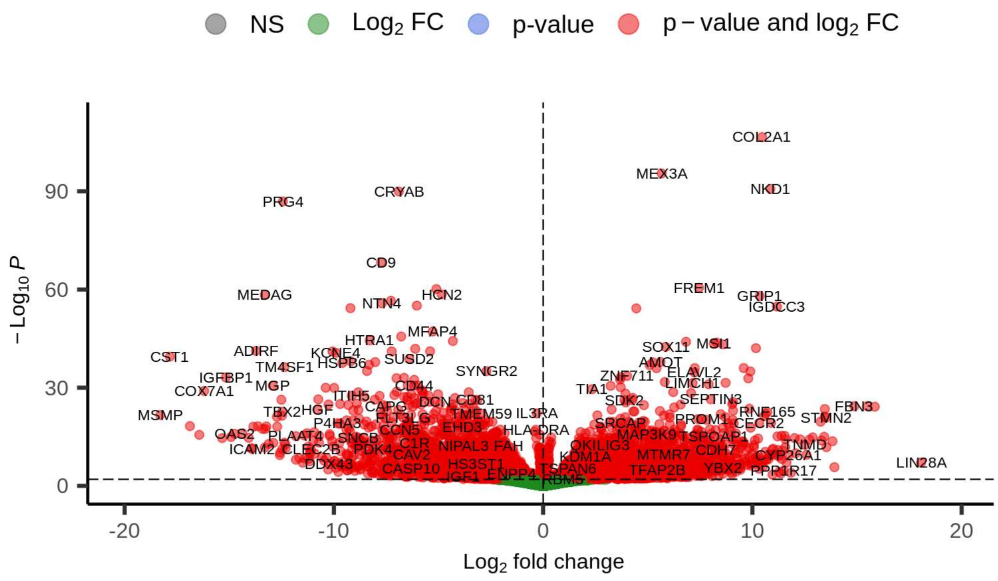

total = 24006 variables

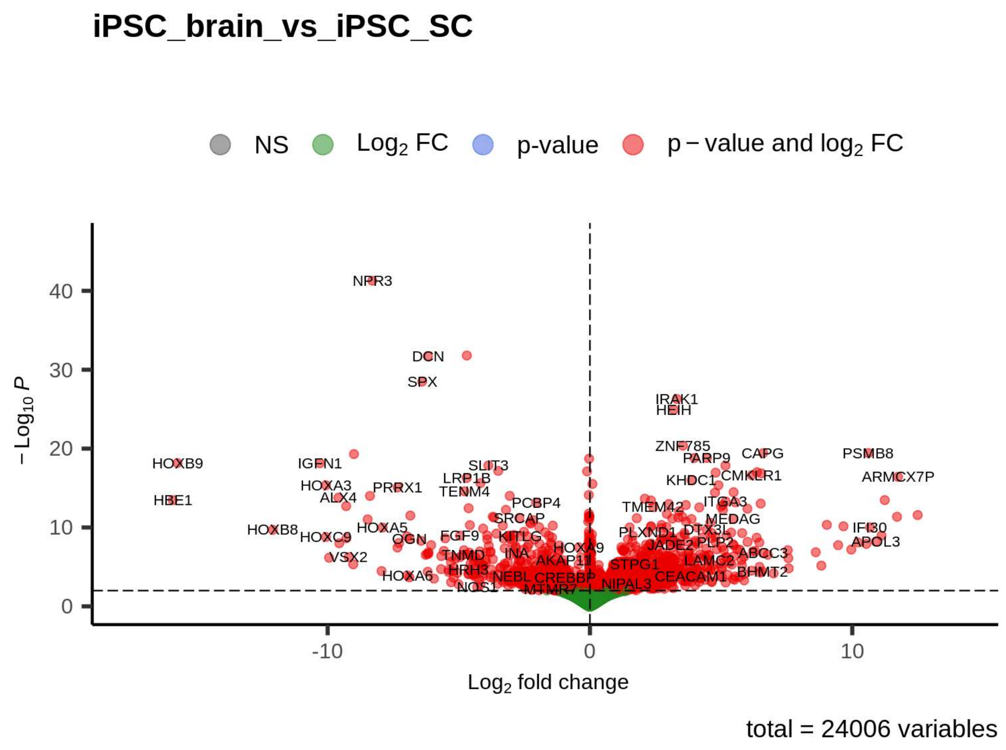

Compare Results

The section below compares the fold change values in the two sets of iPSCs when compared to primary SCs. The sections below (1) List the number of genes significant in each treatment, and in both treatments (intersection); (2) Plot the fold change under each treatment for all genes and for only protein-coding genes; (3) Summarizes the direction of the fold change in each treatment for all genes and for only protein-coding genes.

Fold difference in iPSC-Brain and iPSC-SC vs primary SCSC

The comparison below shows that many genes show similar significant fold changes in both iPSC cell types vs primary SCs. There are 4,586 genes that are significantly changed in the same direction in both comparisons, but also 969 significantly different only in brain iPSCs, and 4,151 significant only in spinal cord iPSCs.

Summary of gene numbers.

- In iPSC_brain_vs_primary_SCSC a total of 24,006 genes were analysed. Of these, 5,555 were significantly differenitally expressed at a padj cutoff of 0.05
- In iPSC_SC_vs_primary_SCSC a total of 24,006 genes were analysed. Of these, 8,737 were significantly differenitally expressed at a padj cutoff of 0.05
- Of these significant genes, 4,600 were significantly DE in both iPSC_brain and iPSC_SC

Fold change scatter plots

These scatter plots compare the fold change values calculated for iPSC_brain and iPSC_SC for all genes (left) and protein-coding genes only (right). Significantly DE genes are coloured to indicate in which samples the expression change is significant.

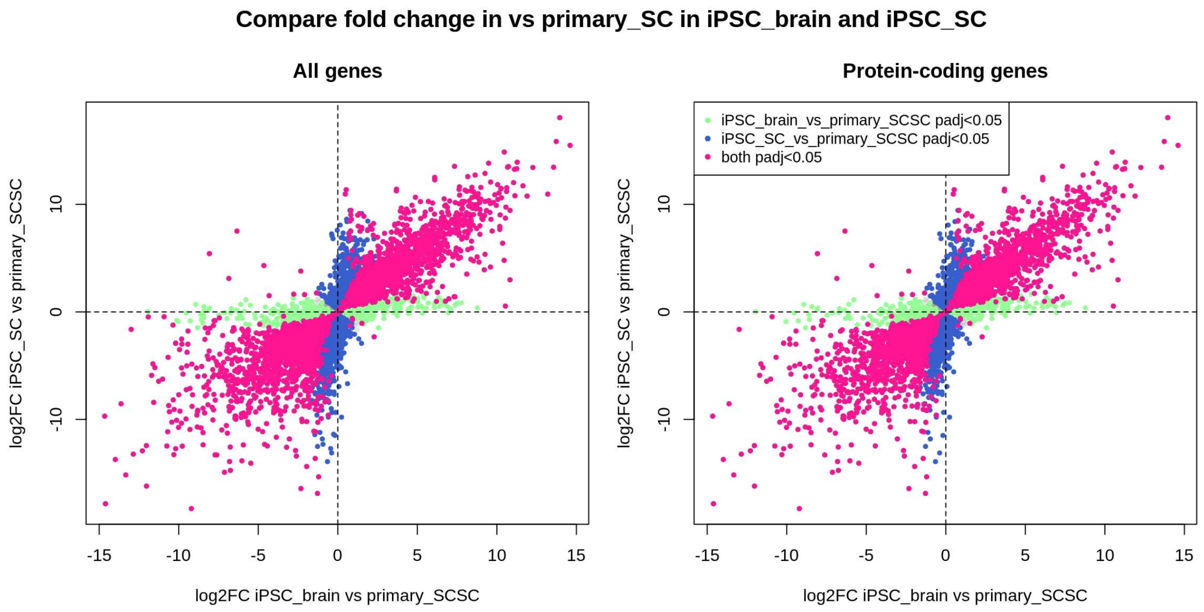

Direction of change

Do the genes significant in both iPSC_brain and iPSC_SC change in the same direction in both comparisons, or are they significantly different in opposite directions?

FC direction of genes significant in both iPSC_brain and iPSC_SC at padj< 0.05

	**All genes**		**Protein-coding genes**	
	**Down in iPSC_SC vs primary_SCSC**	**Up in iPSC_SC vs primary_SCSC**	**Down in iPSC_SC vs primary_SCSC**	**Up in iPSC_SC vs primary_SCSC**
Down in iPSC_brain vs primary_SCSC	2090	**9**	1832	**9**
Up in iPSC_brain vs primary_SCSC	**5**	2496	**5**	2220

Correlation of fold changes for all genes is 0.8049, and for genes significant in either condition is 0.8138.

Genes subsets significant in both iPSC_brain and iPSC_SC, or only one.

Genes with concordant significant FC in vs primary_SC in iPSC_brain and iPSC_SC

This list contains 4,586 genes. The figure below plots vs primary_SC fold change in iPSC_SC vs iPSC_brain.

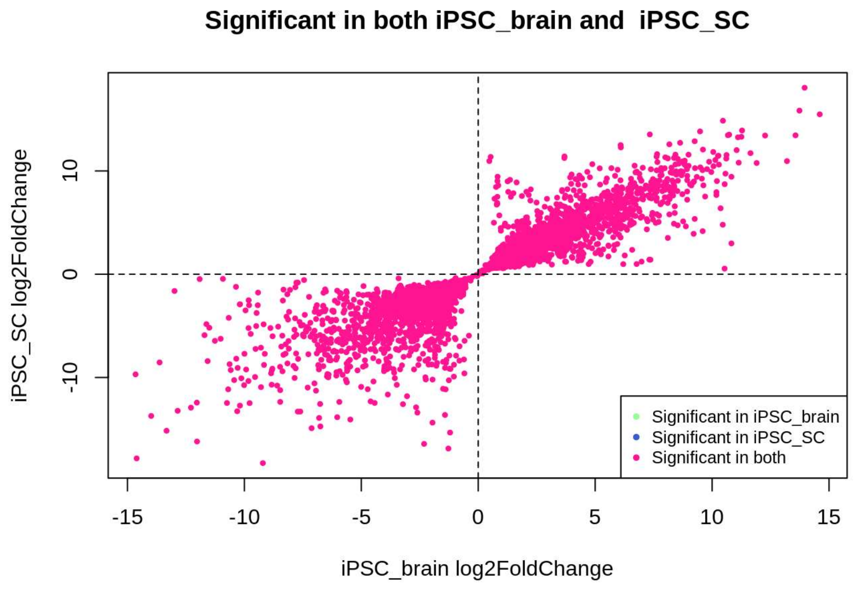

The file compare_FC_iPSC_brain_vs_iPSC_SC_vs_primary_sig_iPSC_brain_and_iPSC_SC.txt (http://www.ogic.ca/projects/ahmad_galuta/human_spinal_cord_SC_iPSC/results/compare_FC_iPSC_brain_vs_iPSC_SC_vs_primary_sig_iPSC_brain_and_iPSC_ lists these genes with fold change data in both iPSC_brain and iPSC_SC samples.

Genes significantly changed in iPSC_brain only

This list contains 969 genes. The figure below plots vs primary_SC fold change in iPSC_SC vs iPSC_brain.

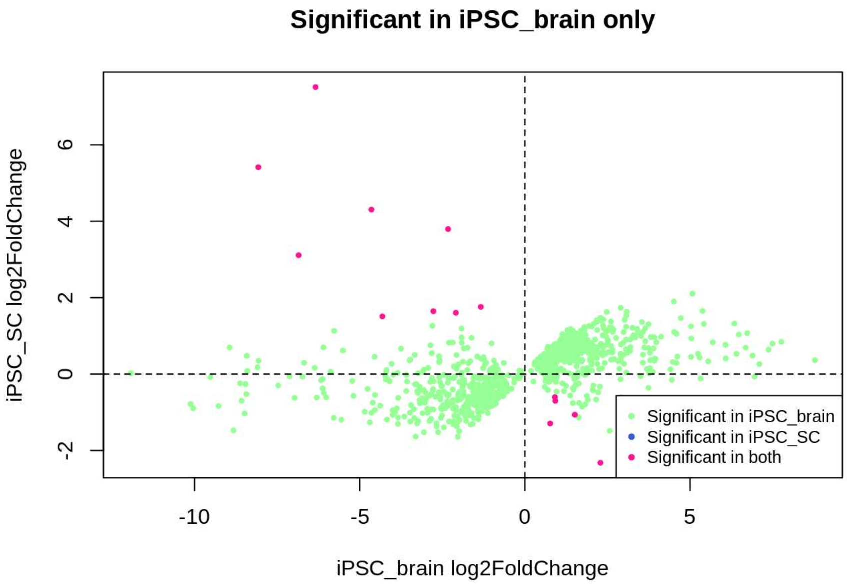

The file compare_FC_iPSC_brain_vs_iPSC_SC_vs_primary_sig_iPSC_brain_only.txt (http://www.ogic.ca/projects/ahmad_galuta/human_spinal_cord_SC_iPSC/results/compare_FC_iPSC_brain_vs_iPSC_SC_vs_primary_sig_iPSC_brain_only.txt) lists these genes with fold change data in both iPSC_brain and iPSC_SC samples.

Genes significantly changed in iPSC_SC only

This list contains 4,151 genes. The figure below plots vs primary_SC fold change in iPSC_SC vs iPSC_brain.

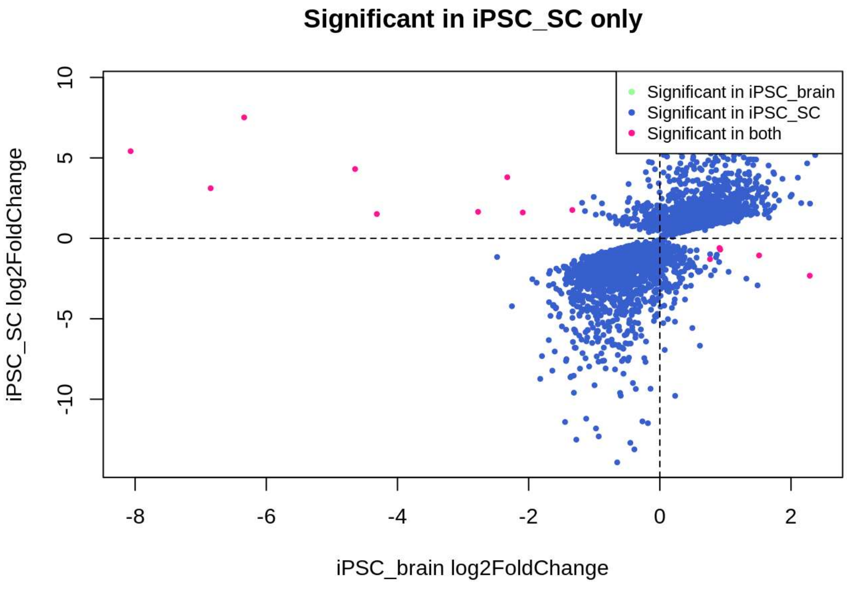

The file compare_FC_iPSC_brain_vs_iPSC_SC_vs_primary_sig_iPSC_SC_only.txt (http://www.ogic.ca/projects/ahmad_galuta/human_spinal_cord_SC_iPSC/results/compare_FC_iPSC_brain_vs_iPSC_SC_vs_primary_sig_iPSC_SC_only.txt) lists these genes with fold change data in both iPSC_brain and iPSC_SC samples.

Venn diagram of genes with significant fold change in vs primary_SC

The intersection between genes significantly up and down in iPSC_brain and iPSC_SC vs primary_SC samples is illustrated below:

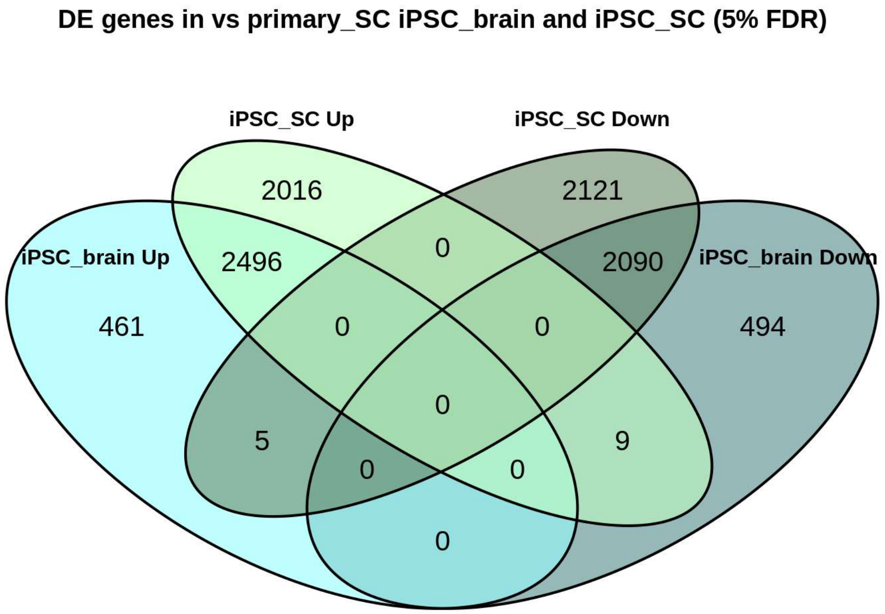

Heatmap of significantly DE genes

The figures below show heatmaps for genes that are significantly DE in either brain or spinal cord iPSCs vs primaries; the heatmap shading represents a Z-score for normalized read counts for each gene across all twelve replicates. The colour bars to the left of the main heatmap show the log2 fold change in the cell type being focused on, and the mean expression indicates whether the gene is expressed at a low or a high level.

Genes significantly DE in iPSC_brain vs primary_SCSC

For this heatmap, the majority of the genes (4,586 of 5,555) are significantly DE in both iPSC types vs primary SCs.

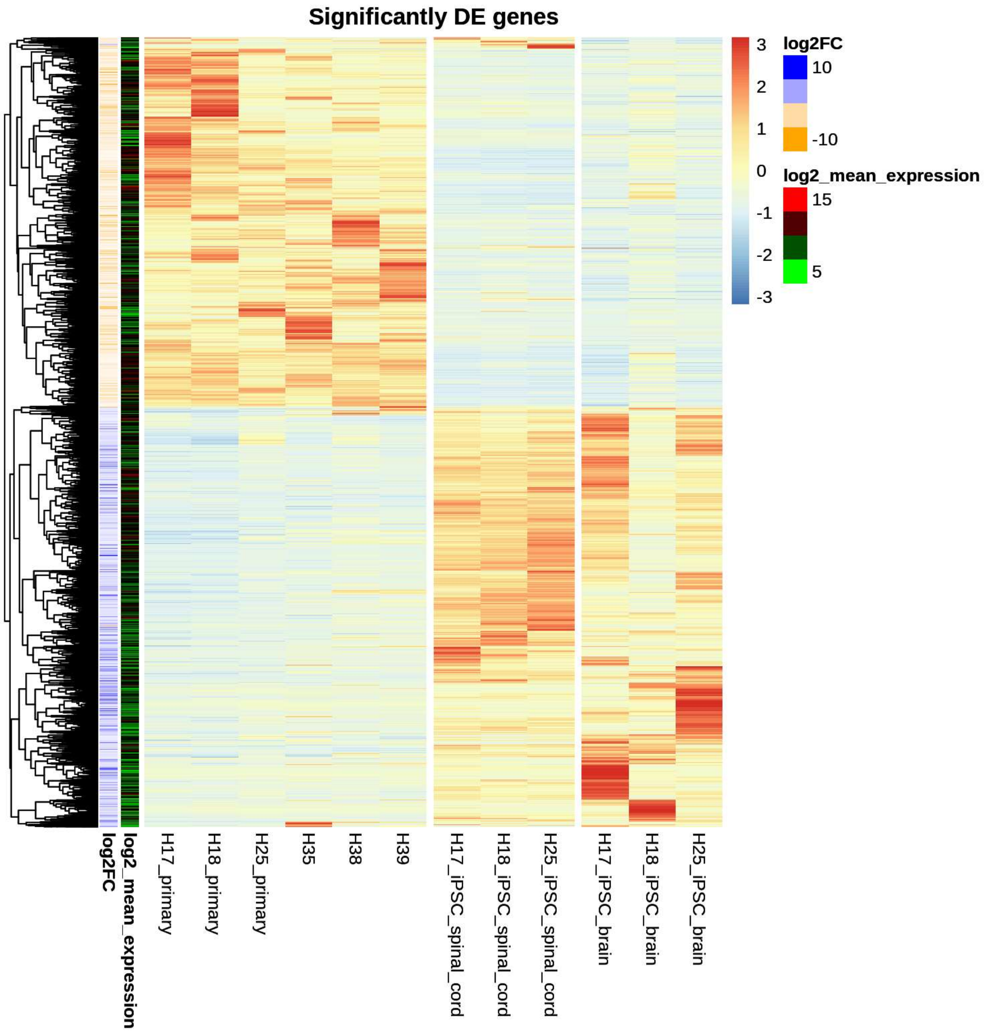

Genes significantly DE in iPSC_SC vs primary_SCSC

For the spinal cord iPSC heatmap, the total number of genes is 8,737, of which the same 4,586 are significantly DE in both brain and spinal cord iPSCs.

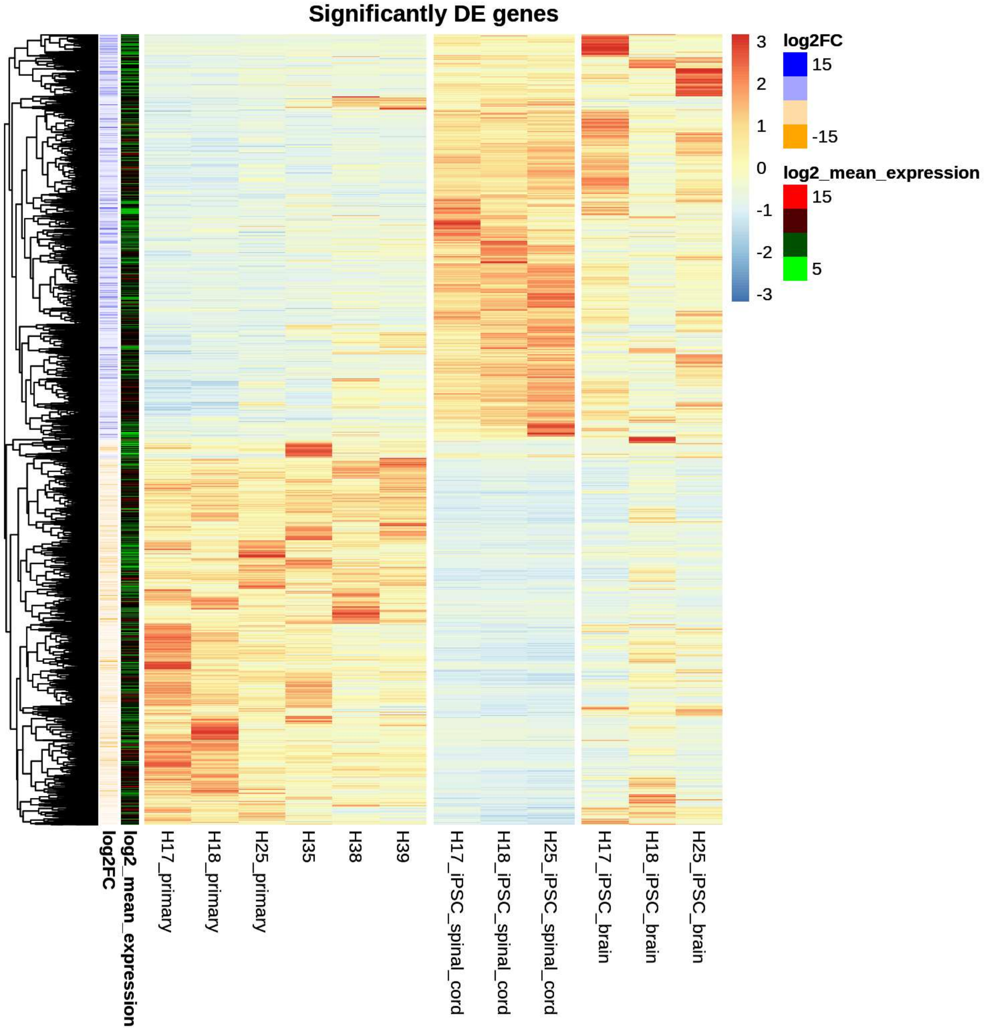

Comparison to Ependymal cell DE gene list from single cell data.

I took the markers for the Ependymal cell cluster from the single cell gene expression dataset provided by Ariel Levine's lab, and used these to plot the heatmap below. I restricted the analysis to genes with an adjusted p-value < 0.05 (5% FDR), and which are found in fewer than 5% of cells outside the ependymal cluster - these should be the genes most specific to ependymal cells. This filters the set of 1,925 genes down to 516, of which 435 are found in the results set.

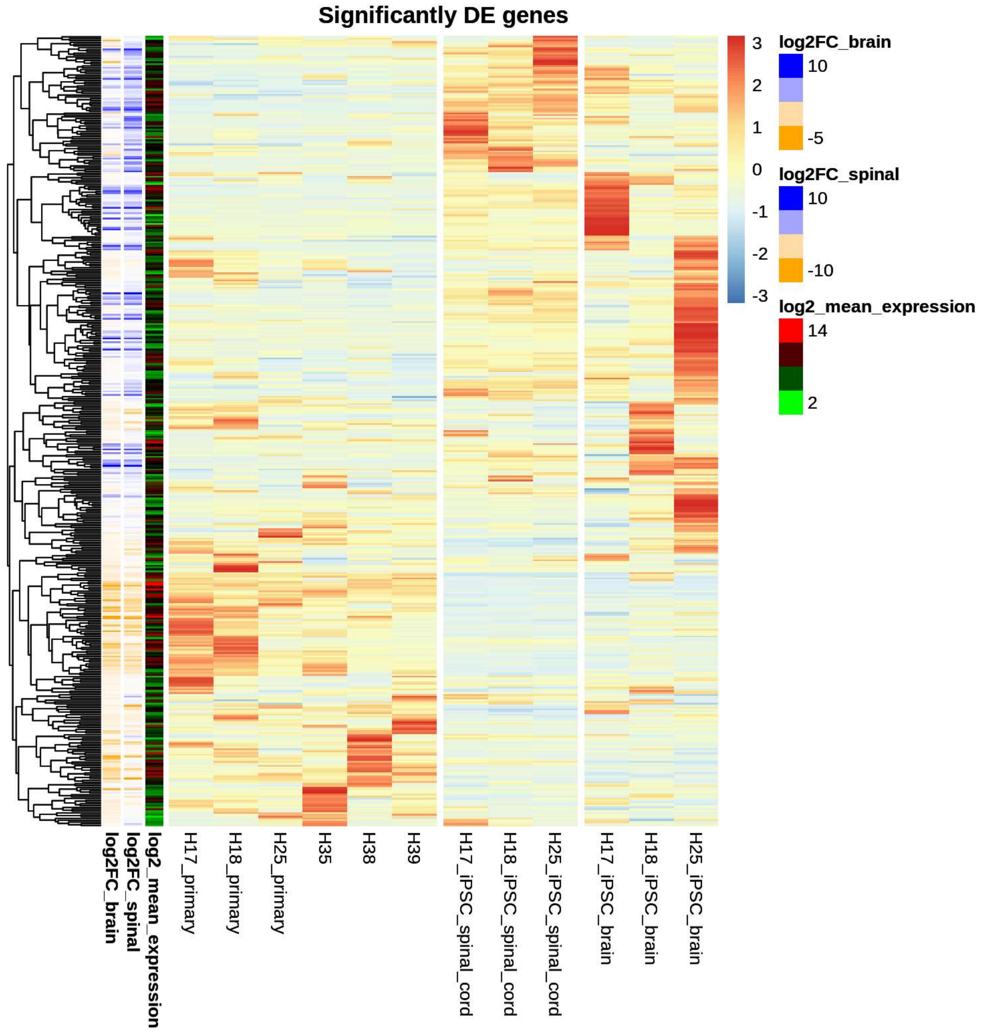

The clustering in this heatmap doesn't show an obvious shift towards an ependymal phenotype in any of the three cell types, with subsets of the genes apparently higher in both primary and iPSC cells.

Other markers of interest

Ahmad provided specific gene lists to be investigated. For each set I've plotted a heatmap, followed by

Spinal Cord

```
## [1] "Found data for 33 of 40 genes in the list"
```

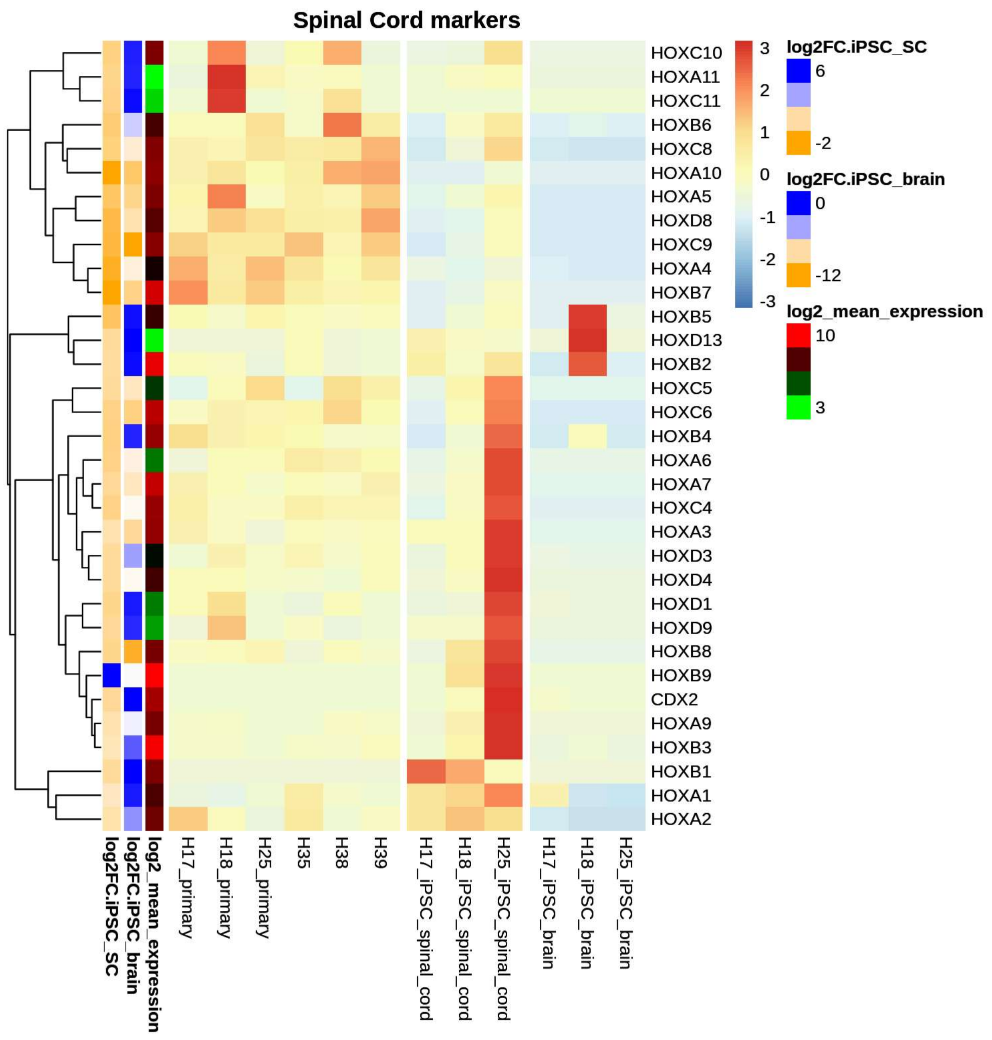
Spinal Cord markers
HOXC10
HOXA11
HOXC11
HOXB6
HOXC8
HOXA10
HOXA5
HOXD8
HOXC9
HOXA4
HOXB7
HOXB5
HOXD13
HOXB2
HOXC5
HOXC6
HOXB4
HOXA6
HOXA7
HOXC4
HOXA3
HOXD3
HOXD4
HOXD1
HOXD9
HOXB8
HOXB9
CDX2
HOXA9
HOXB3
HOXB1
HOXA1
HOXA2
3
2
1
0
-1
-2
-3
log2FC.iPSC_SC
6
-2
log2FC.iPSC_brain
0
-12
log2_mean_expression
10
3
log2FC.iPSC_SC
log2FC.iPSC_brain
log2_mean_expression
H17_primary
H18_primary
H25_primary
H35
H38
H39
H17_iPSC_spinal_cord
H18_iPSC_spinal_cord
H25_iPSC_spinal_cord
H17_iPSC_brain
H18_iPSC_brain
H25_iPSC_brain

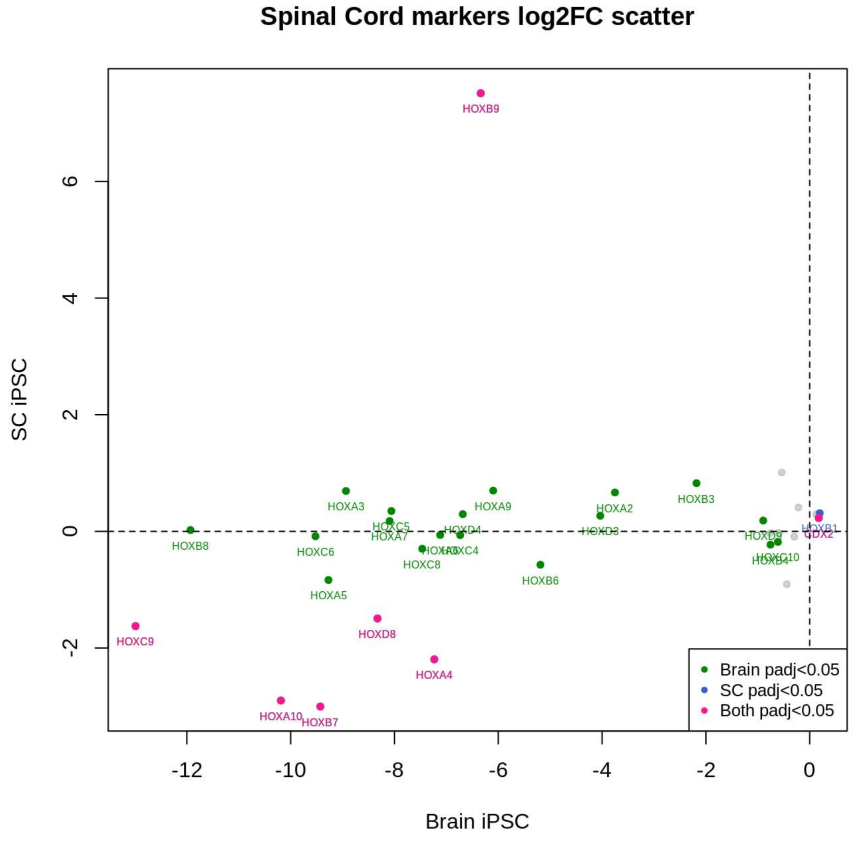
Spinal Cord markers log2FC scatter
SC iPSC
Brain iPSC
6
4
2
0
-2
-12
-10
-8
-6
-4
-2
0
HOXB9
HOXA3
HOXA9
HOXA2
HOXB3
HOXC5
HOXA7
HOXD4
HOXD3
HOXB1
CDX2
HOXB8
HOXC6
HOXC4
HOXC8
HOXB6
HOXD9
HOXC10
HOXA5
HOXD8
HOXC9
HOXA4
HOXA10
HOXB7
Brain padj<0.05
SC padj<0.05
Both padj<0.05

Most of the genes are significantly lower in brain iPSCs vs spinal cord iPSCs, and not significantly changed in spinal cord iPSCs. Two genes are significantly higher in brain iPSCs (HOXB1 and CDX2), although the magnitude of the fold change has been shrunk almost to zero; CDX2 is also significantly up in spinal cord iPSCs.

Brain genes

```
## [1] "Found data for 15 of 16 genes in the list"
```

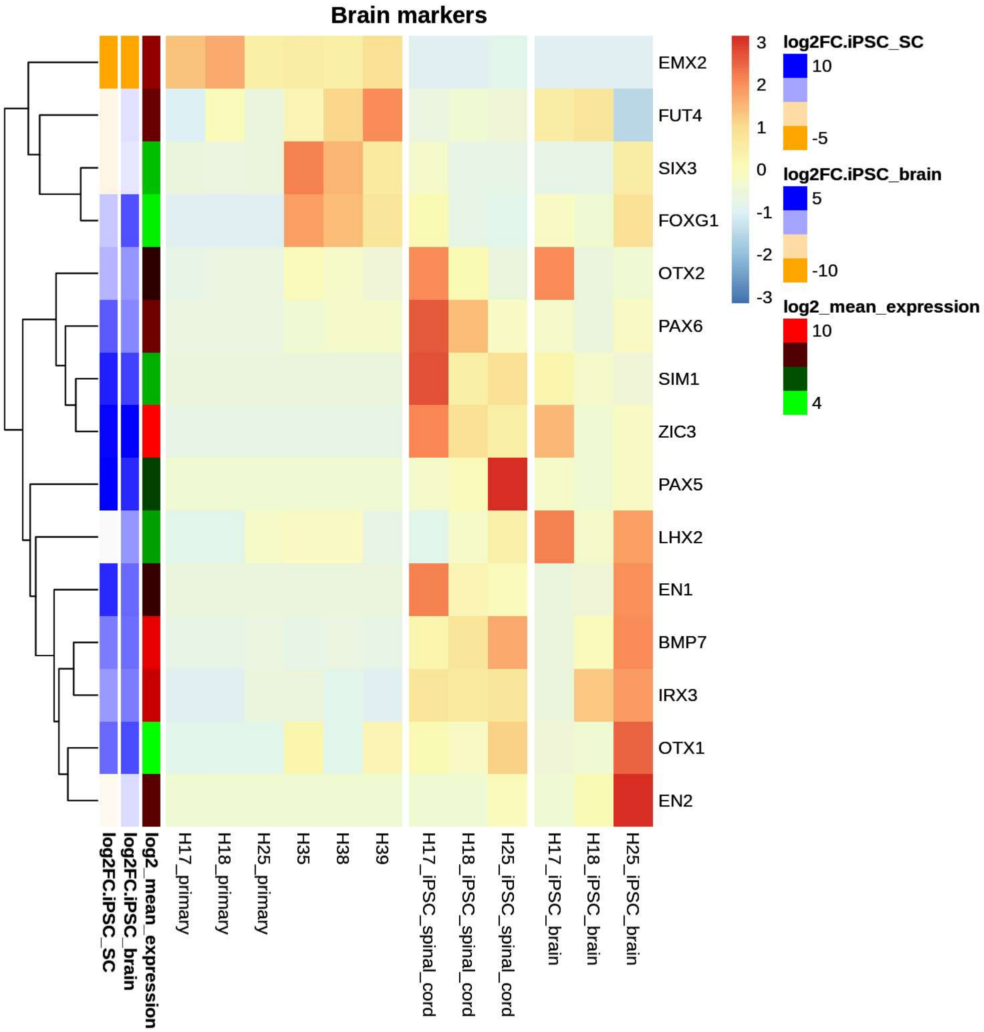

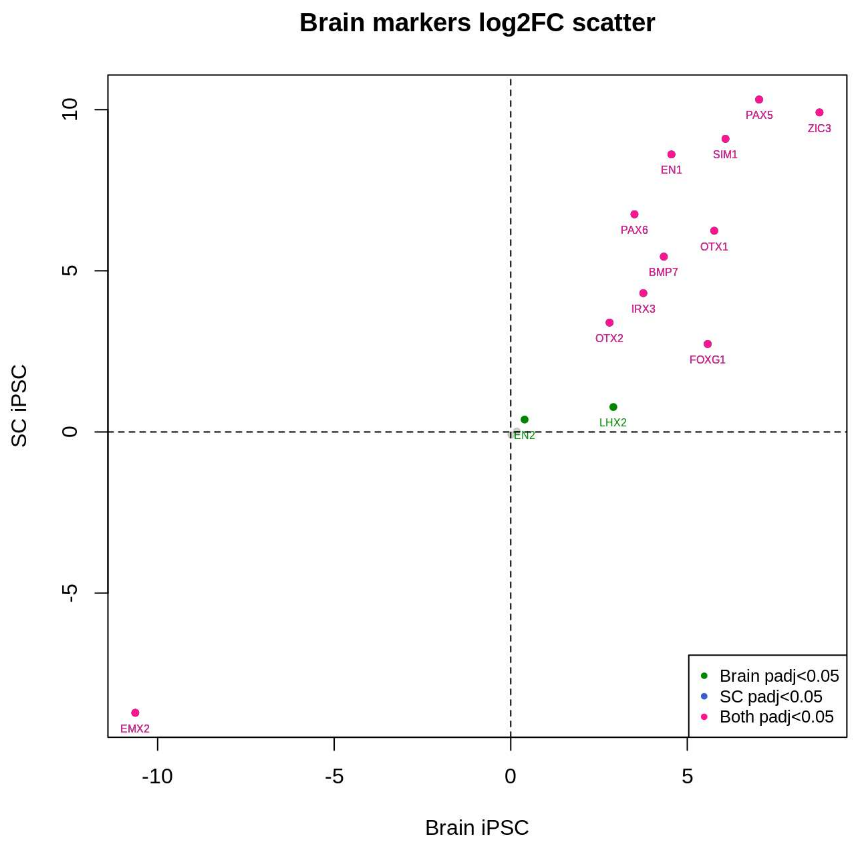

Most of the brain markers are signficantly higher in both brain and spinal cord iPSCs; the exception is EMX2 which is significantly down in both iPSC datasets. Fold changes in both directions are very large for some genes.

Msoderm genes

```
## [1] "Found data for 3 of 3 genes in the list"
```

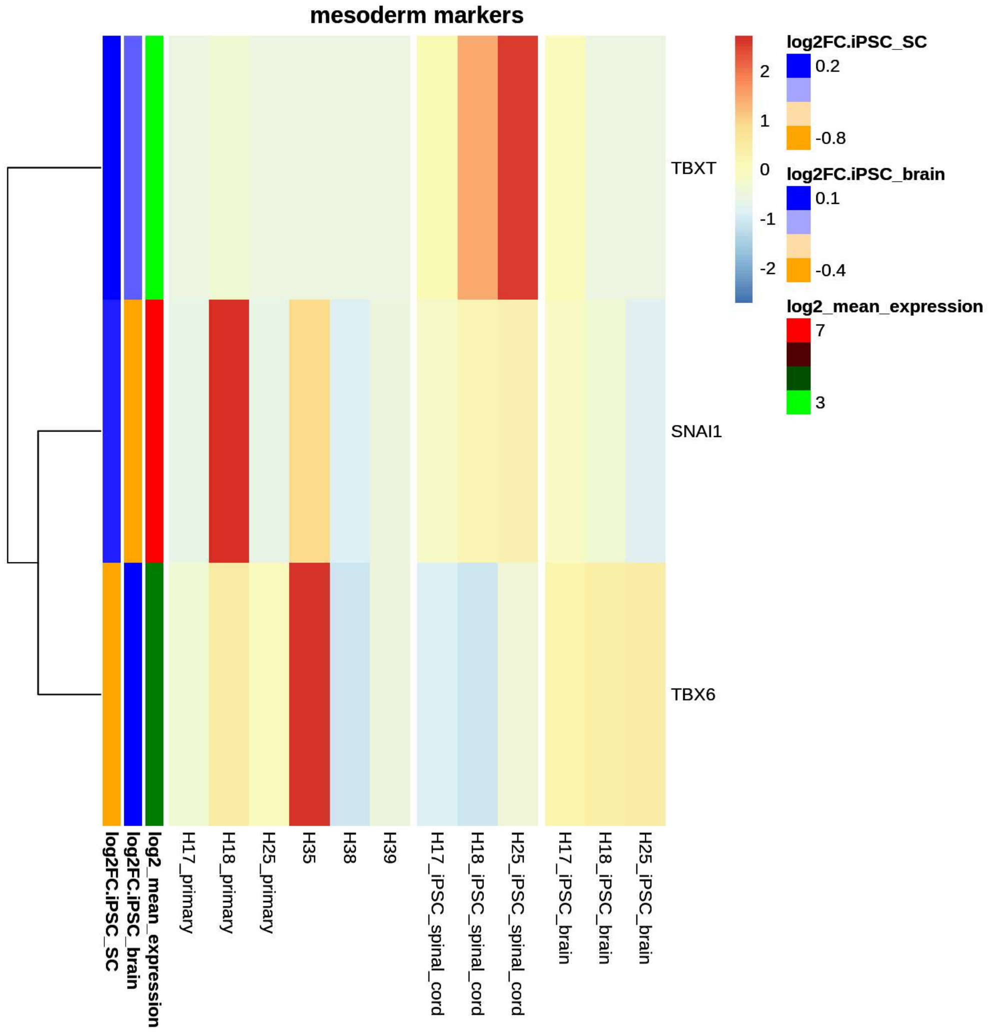
mesoderm markers
TBXT
SNAI1
TBX6
log2FC.iPSC_SC
log2FC.iPSC_brain
log2_mean_expression
H17_primary
H18_primary
H25_primary
H35
H38
H39
H17_iPSC_spinal_cord
H18_iPSC_spinal_cord
H25_iPSC_spinal_cord
H17_iPSC_brain
H18_iPSC_brain
H25_iPSC_brain
2
1
0
-1
-2
log2FC.iPSC_SC
0.2
-0.8
log2FC.iPSC_brain
0.1
-0.4
log2_mean_expression
7
3

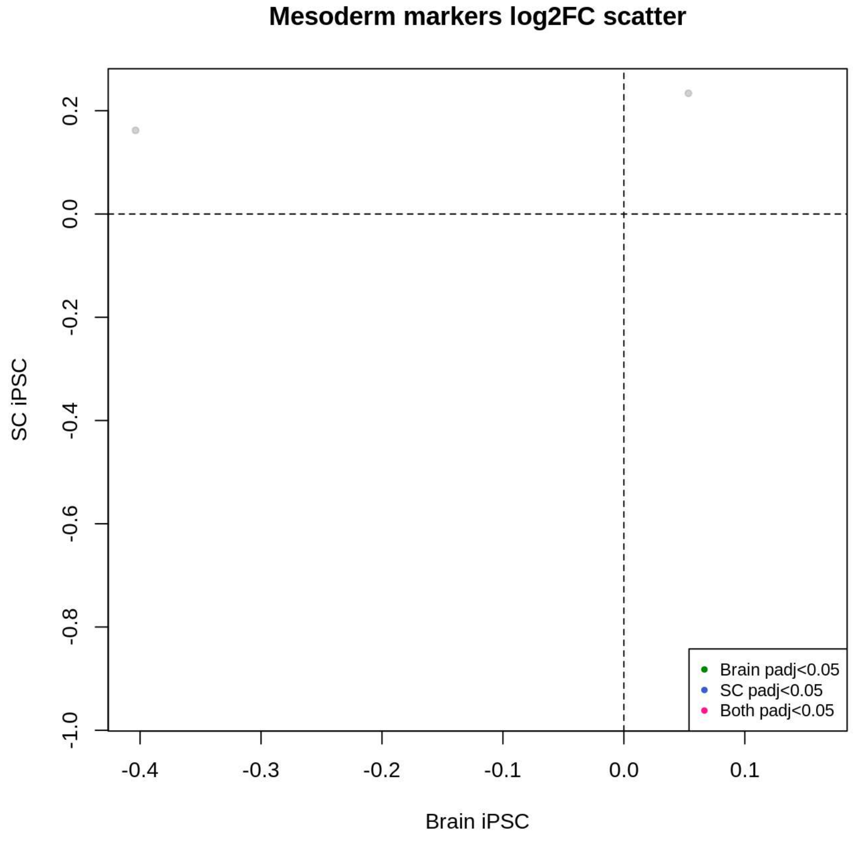
Mesoderm markers log2FC scatter
SC iPSC
Brain iPSC
0.2
0.0
-0.2
-0.4
-0.6
-0.8
-1.0
-0.4
-0.3
-0.2
-0.1
0.0
0.1
Brain padj<0.05
SC padj<0.05
Both padj<0.05

None of these three genes are significantly DE in either set of iPSCs vs primaries.

NSC genes

```
## [1] "Found data for 9 of 9 genes in the list"
```

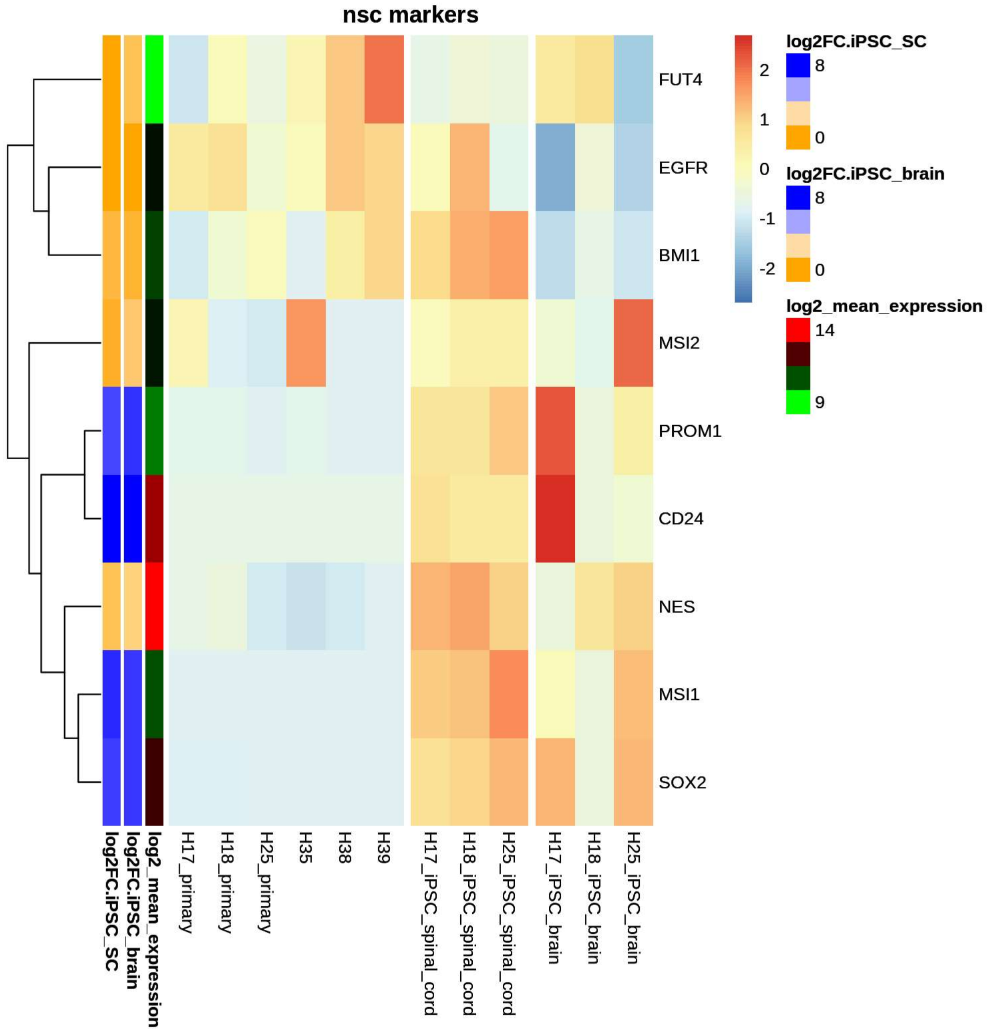

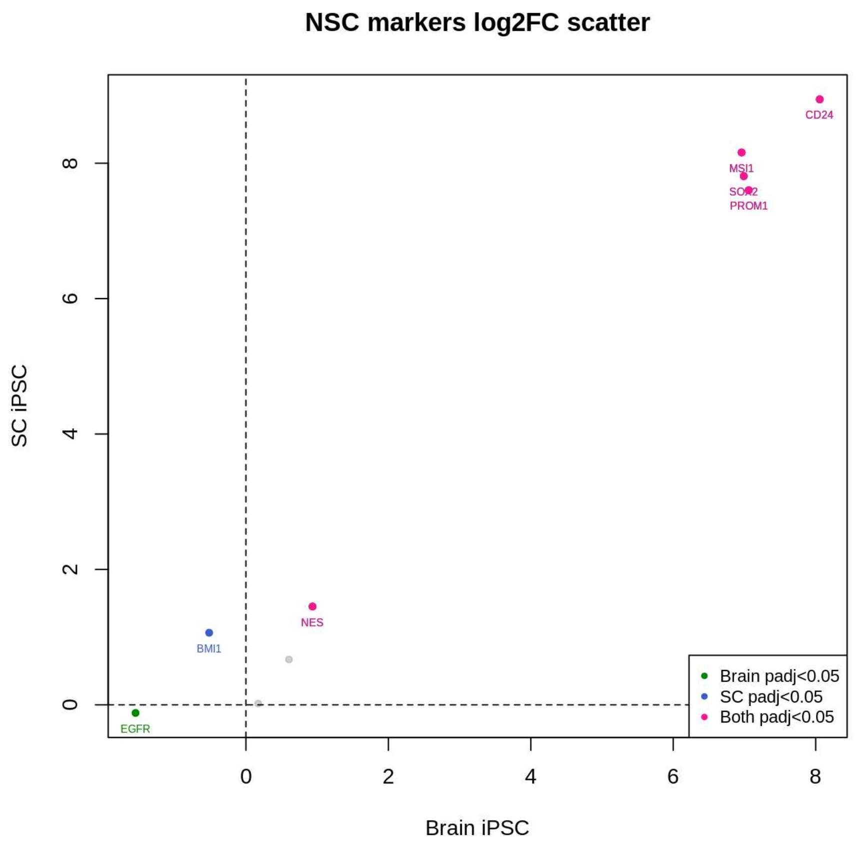

Five of the nine genes are significantly up in both brain and spinal cord iPSCs vs primaries; one gene (EGFR) is significantly down in brain, but unchanged in spinal cord iPSCs; one gene is up in spinal cord iPSCs but unchanged in brain.

Ependymal genes

```
## [1] "Found data for 42 of 44 genes in the list"
```

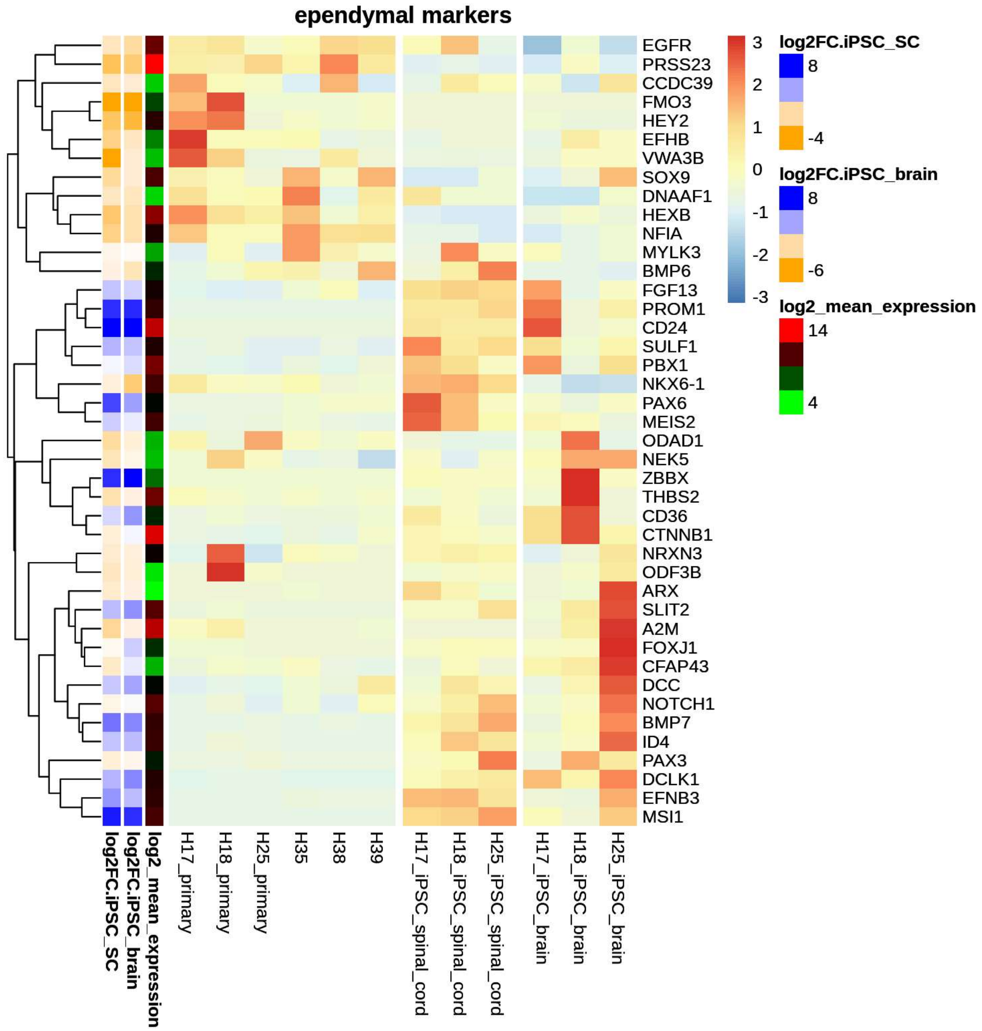
ependymal markers
EGFR
PRSS23
CCDC39
FMO3
HEY2
EFHB
VWA3B
SOX9
DNAAF1
HEXB
NFIA
MYLK3
BMP6
FGF13
PROM1
CD24
SULF1
PBX1
NKX6-1
PAX6
MEIS2
ODAD1
NEK5
ZBBX
THBS2
CD36
CTNNB1
NRXN3
ODF3B
ARX
SLIT2
A2M
FOXJ1
CFAP43
DCC
NOTCH1
BMP7
ID4
PAX3
DCLK1
EFNB3
MSI1
3
2
1
0
-1
-2
-3
log2FC.iPSC_SC
8
-4
log2FC.iPSC_brain
8
-6
log2_mean_expression
14
4
log2FC.iPSC_SC
log2FC.iPSC_brain
log2_mean_expression
H17_primary
H18_primary
H25_primary
H35
H38
H39
H17_iPSC_spinal_cord
H18_iPSC_spinal_cord
H25_iPSC_spinal_cord
H17_iPSC_brain
H18_iPSC_brain
H25_iPSC_brain

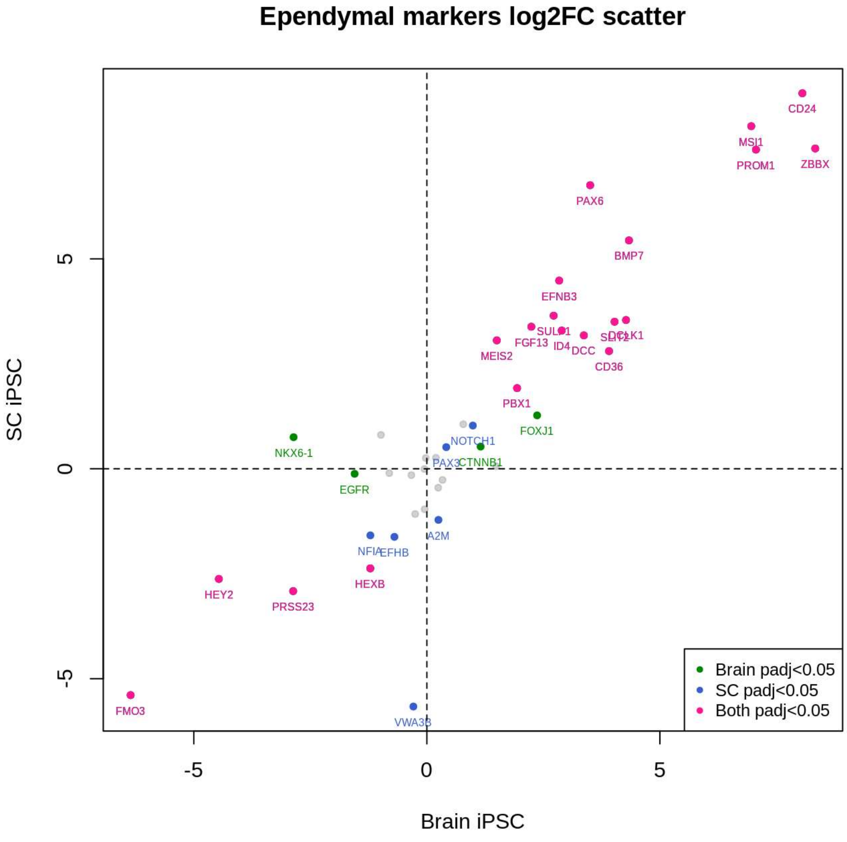
Ependymal markers log2FC scatter
SC iPSC
Brain iPSC
-5
0
5
CD24
MSI1
PROM1
ZBBX
PAX6
BMP7
EFNB3
FGF13
SULF1
ID4
DCC
SLIT2
DCLK1
CD36
MEIS2
PBX1
FOXJ1
NKX6-1
NOTCH1
PAX3
CTNNB1
EGFR
A2M
NFIA
EFHB
HEXB
HEY2
PRSS23
FMO3
VWA3B
Brain padj<0.05
SC padj<0.05
Both padj<0.05

Of this set of 42 Ependymal genes (of which 44 are in the list from the Levine lab) there are 16 significantly up and 4 significantly down in both brain and spinal cord iPSCs. A further 10 genes are signficantly different in either brain or spinal cord iPSCs; six of them are down vs primary SCs, and four are up.

Proliferation genes

```
## [1] "Found data for 22 of 24 genes in the list"
```

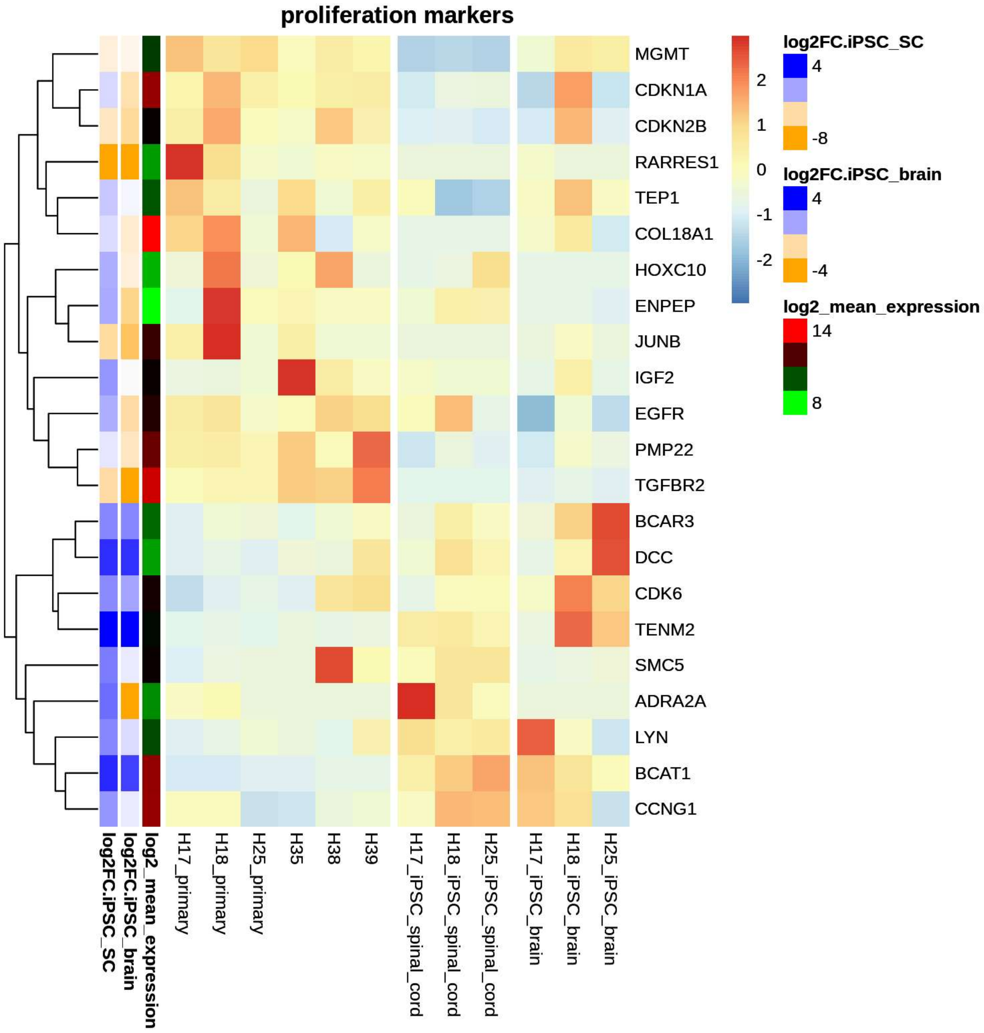

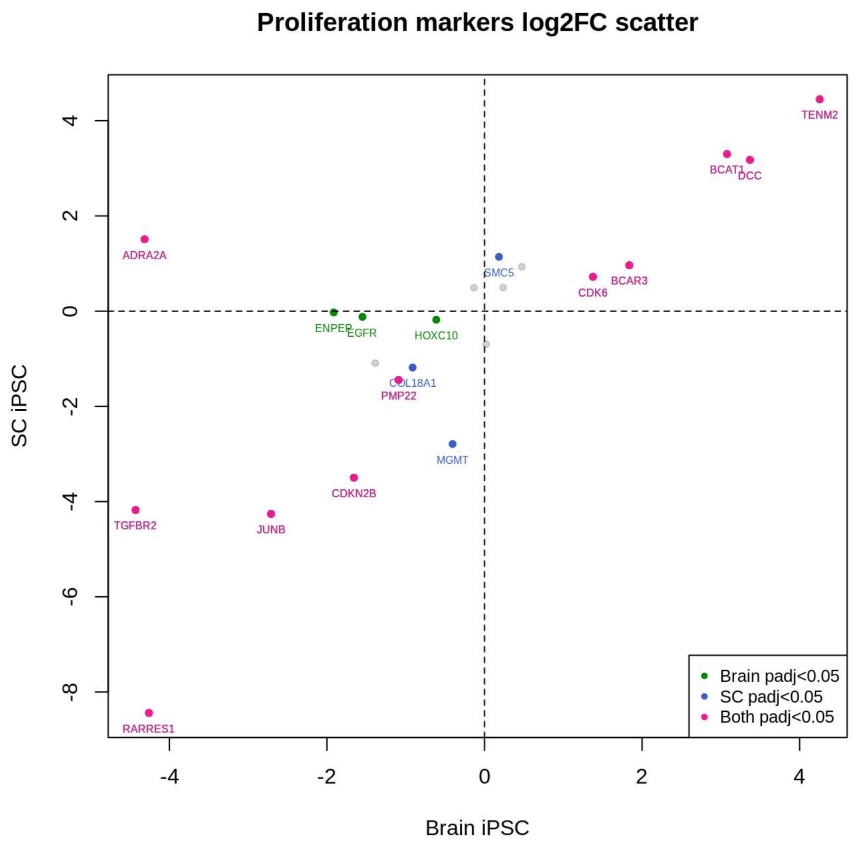

Again, there's no clear pattern for the proliferation genes with some significantly up in both iPSC sets (up to 16-fold), and some significantly down (128-fold for RARRES1 in spinal cord iPSCs, although checking the heatmap this appears to be the result of very high expression in a single primary SC replicate).

Neurogenesis genes

```
## [1] "Found data for 22 of 22 genes in the list"
```

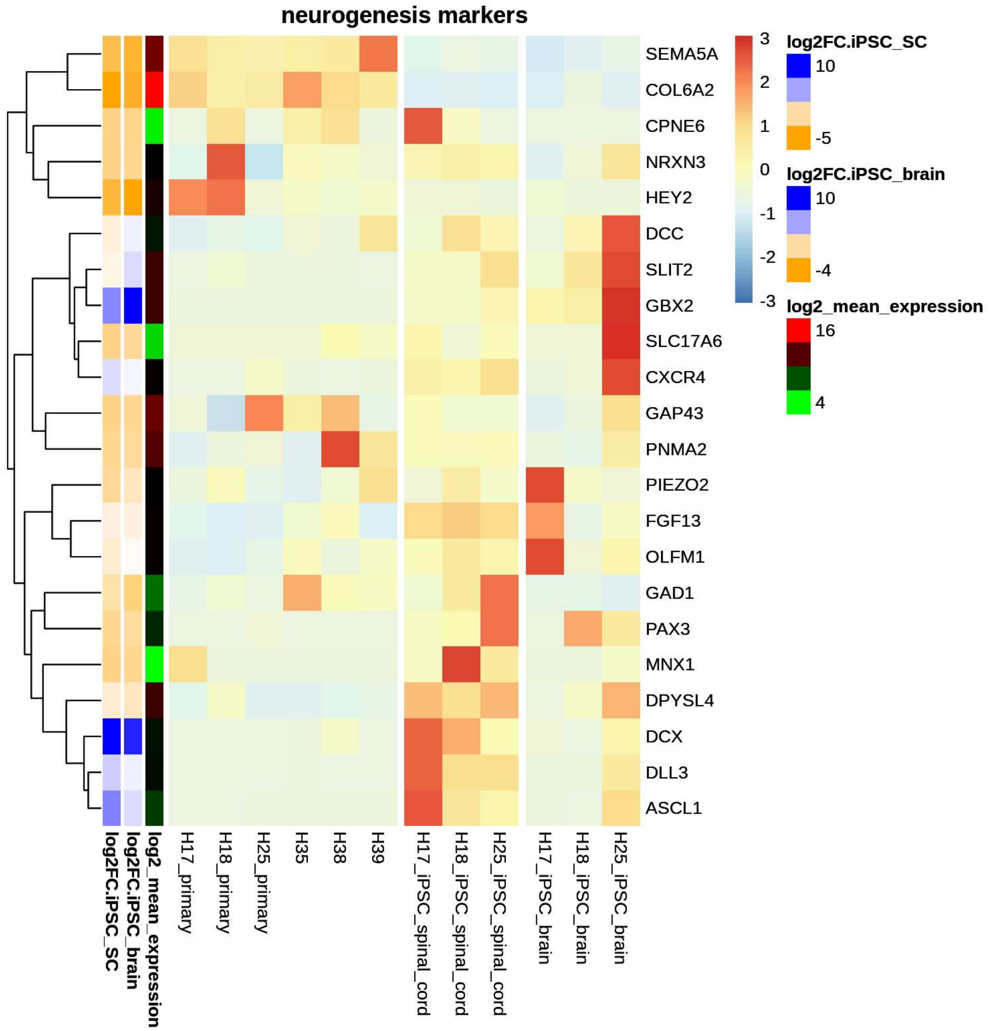

neurogenesis markers
SEMA5A
COL6A2
CPNE6
NRXN3
HEY2
DCC
SLIT2
GBX2
SLC17A6
CXCR4
GAP43
PNMA2
PIEZO2
FGF13
OLFM1
GAD1
PAX3
MNX1
DPYSL4
DCX
DLL3
ASCL1
log2FC.iPSC_SC
log2FC.iPSC_brain
log2_mean_expression
H17_primary
H18_primary
H25_primary
H35
H38
H39
H17_iPSC_spinal_cord
H18_iPSC_spinal_cord
H25_iPSC_spinal_cord
H17_iPSC_brain
H18_iPSC_brain
H25_iPSC_brain
3
2
1
0
-1
-2
-3
log2FC.iPSC_SC
10
-5
log2FC.iPSC_brain
10
-4
log2_mean_expression
16
4

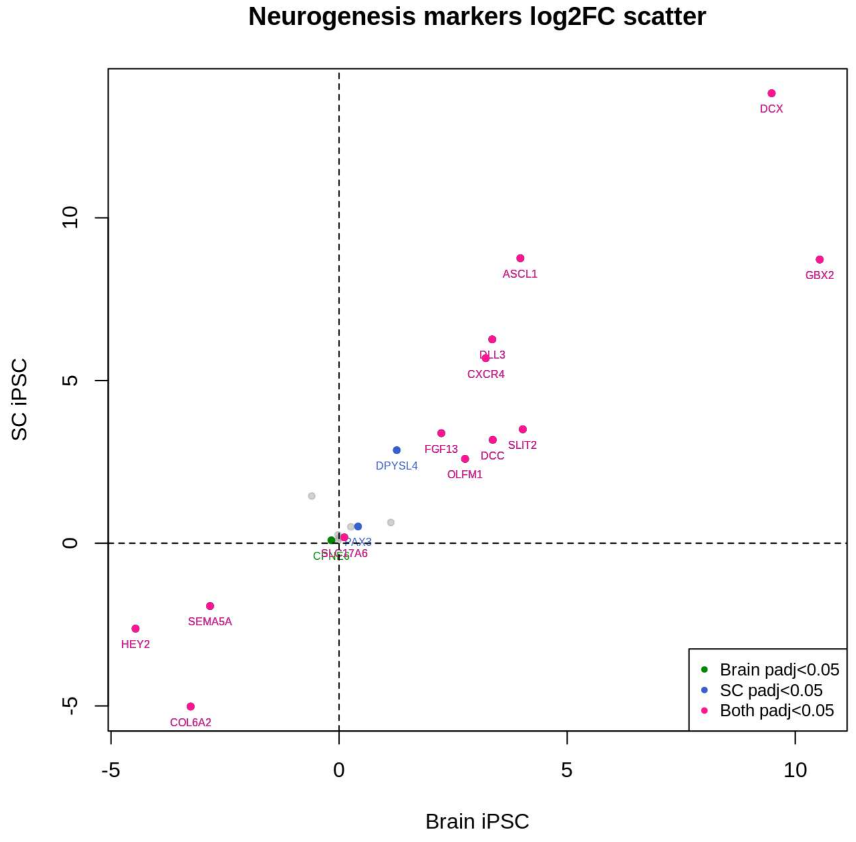

Neurogenesis markers log2FC scatter
SC iPSC
Brain iPSC
DCX
ASCL1
GBX2
DLL3
CXCR4
FGF13
DCC
SLIT2
DPYSL4
OLFM1
PAX3
CPNE6
SLC17A6
SEMA5A
HEY2
COL6A2
Brain padj<0.05
SC padj<0.05
Both padj<0.05
-5
0
5
10

As for the ependymal markers, some are higher in both iPSC groups, some are lower.

Astrogenesis genes

```
## [1] "Found data for 9 of 9 genes in the list"
```

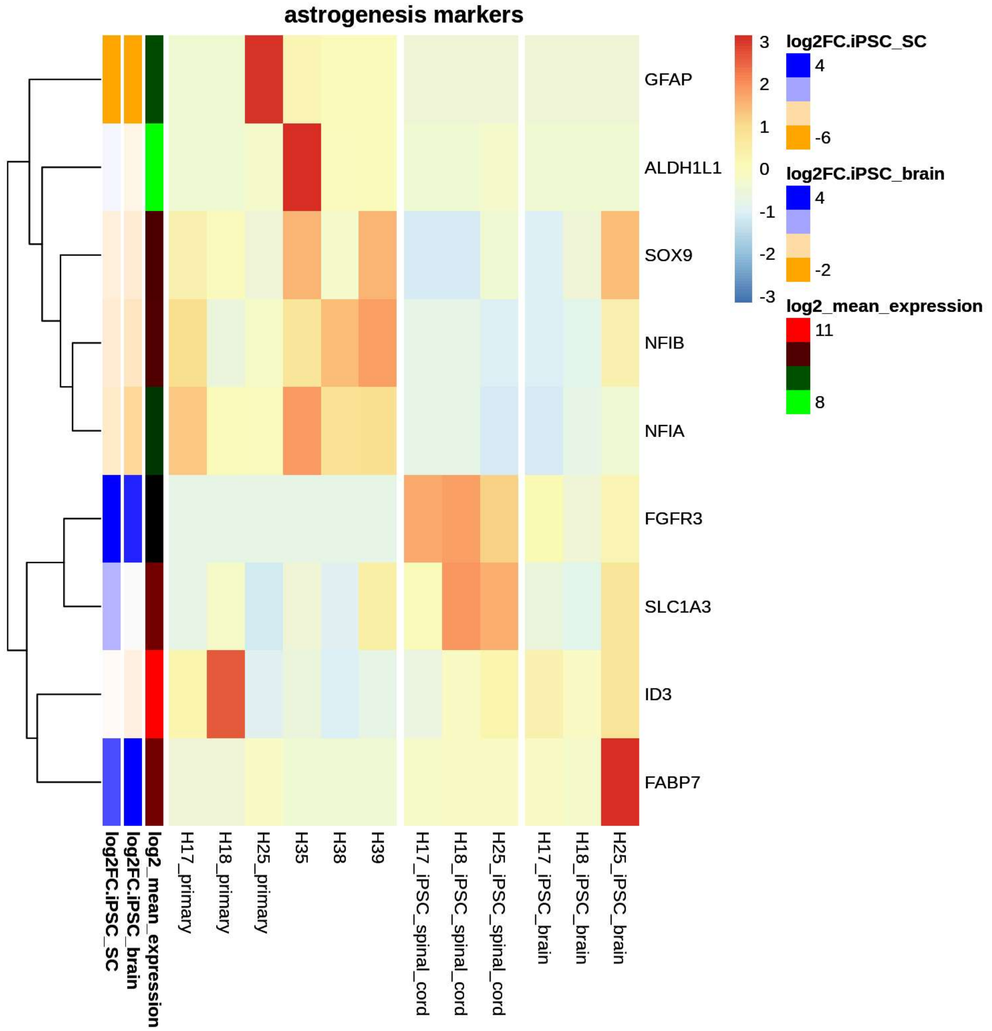

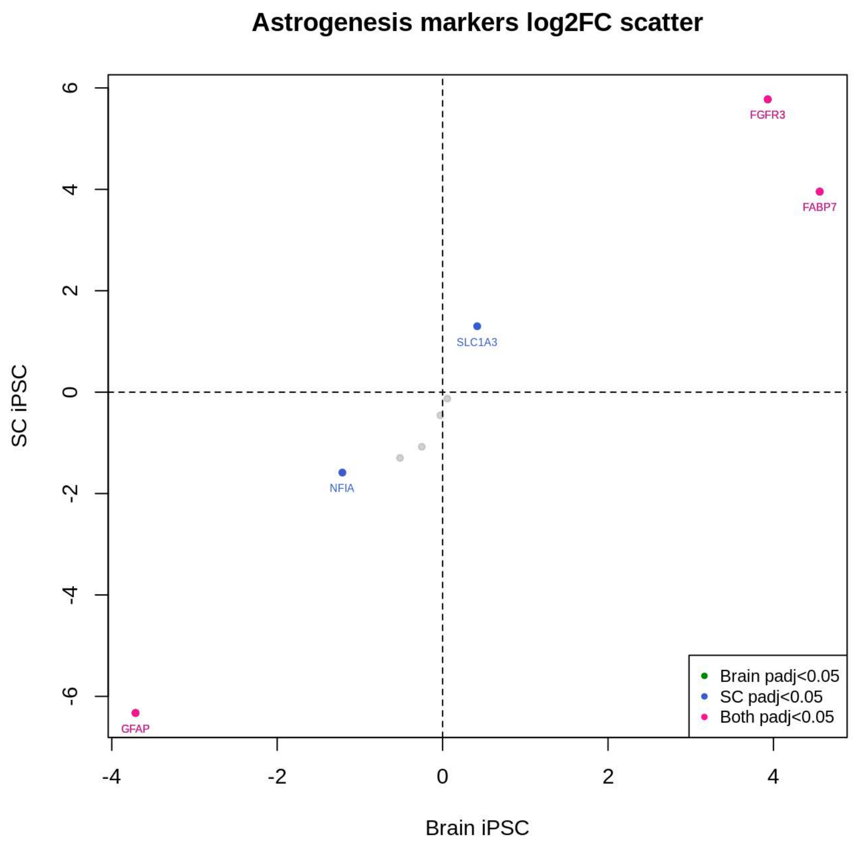

Oligodendrogensis genes

```
## [1] "Found data for 9 of 10 genes in the list"
```

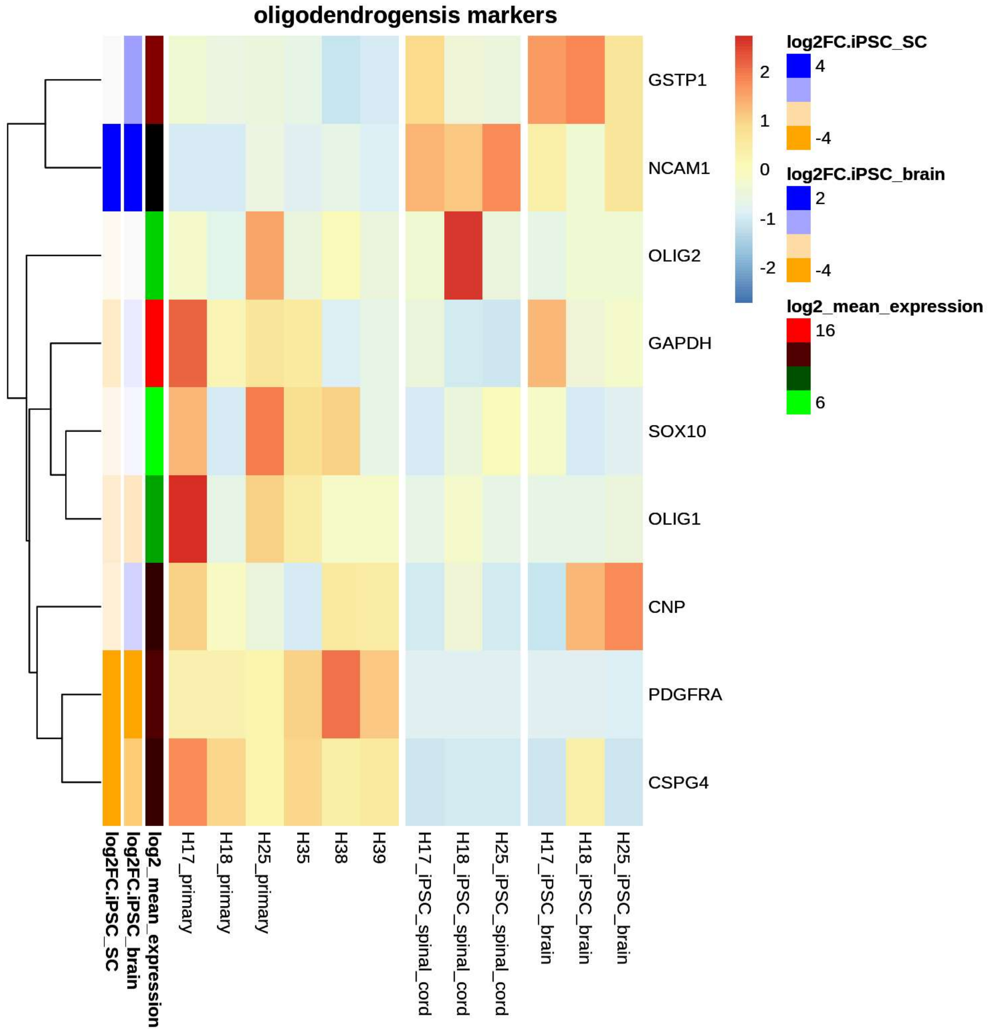

oligodendrogensis markers
GSTP1
NCAM1
OLIG2
GAPDH
SOX10
OLIG1
CNP
PDGFRA
CSPG4
log2FC.iPSC_SC
log2FC.iPSC_brain
log2_mean_expression
H17_primary
H18_primary
H25_primary
H35
H38
H39
H17_iPSC_spinal_cord
H18_iPSC_spinal_cord
H25_iPSC_spinal_cord
H17_iPSC_brain
H18_iPSC_brain
H25_iPSC_brain
2
1
0
-1
-2
log2FC.iPSC_SC
4
-4
log2FC.iPSC_brain
2
-4
log2_mean_expression
16
6

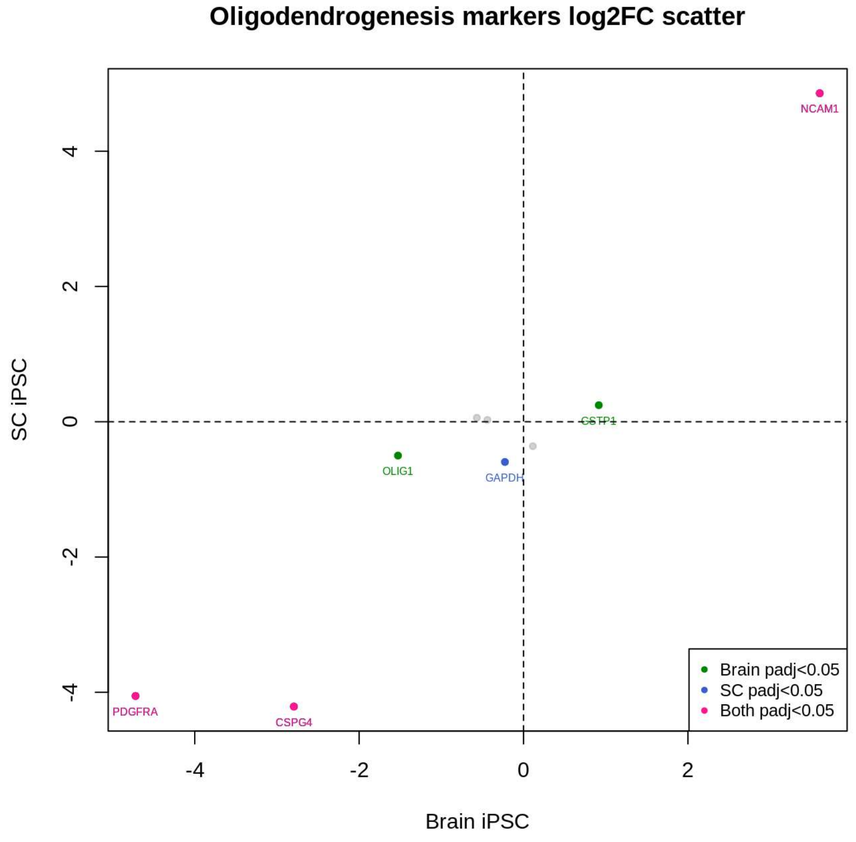

Oligodendrogenesis markers log2FC scatter
SC iPSC
Brain iPSC
NCAM1
GSTP1
OLIG1
GAPDH
PDGFRA
CSPG4
Brain padj<0.05
SC padj<0.05
Both padj<0.05
4
2
0
-2
-4
-4
-2
0
2

Dorsal genes

```
## [1] "Found data for 10 of 11 genes in the list"
```

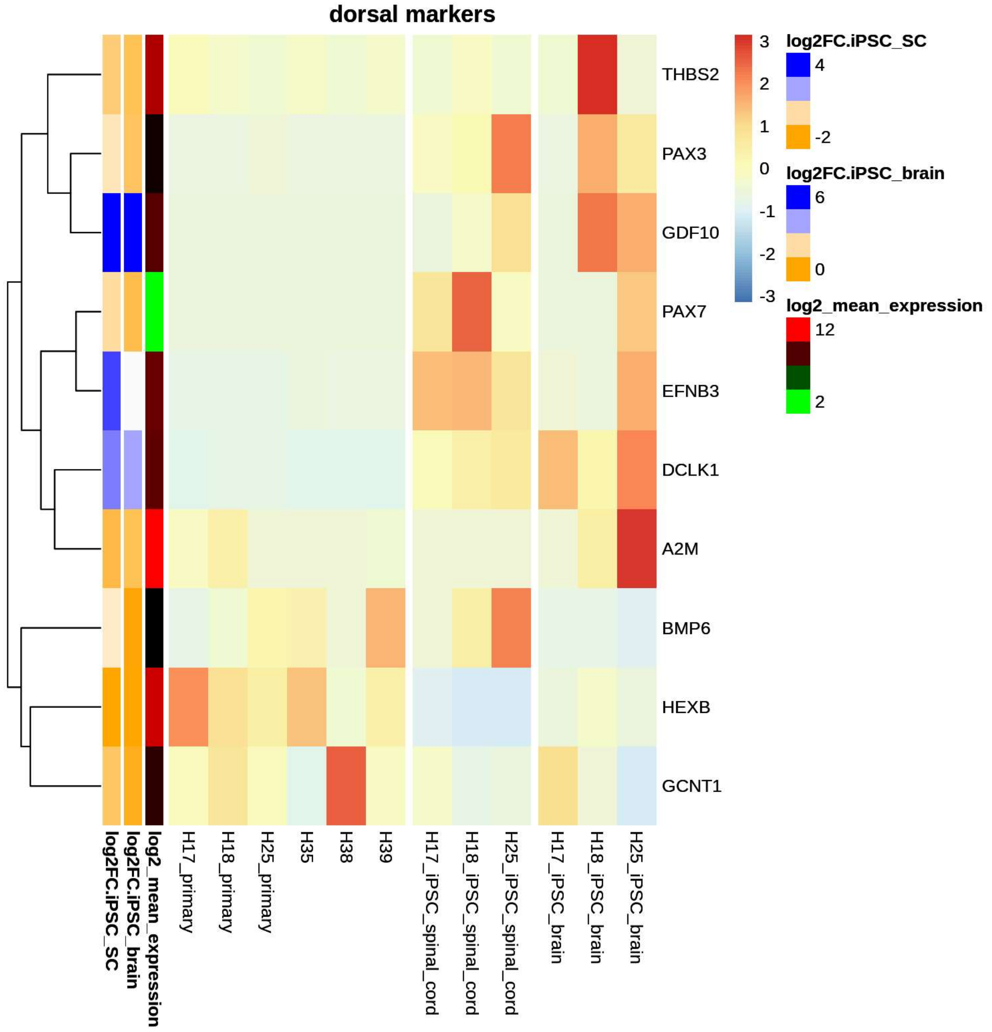

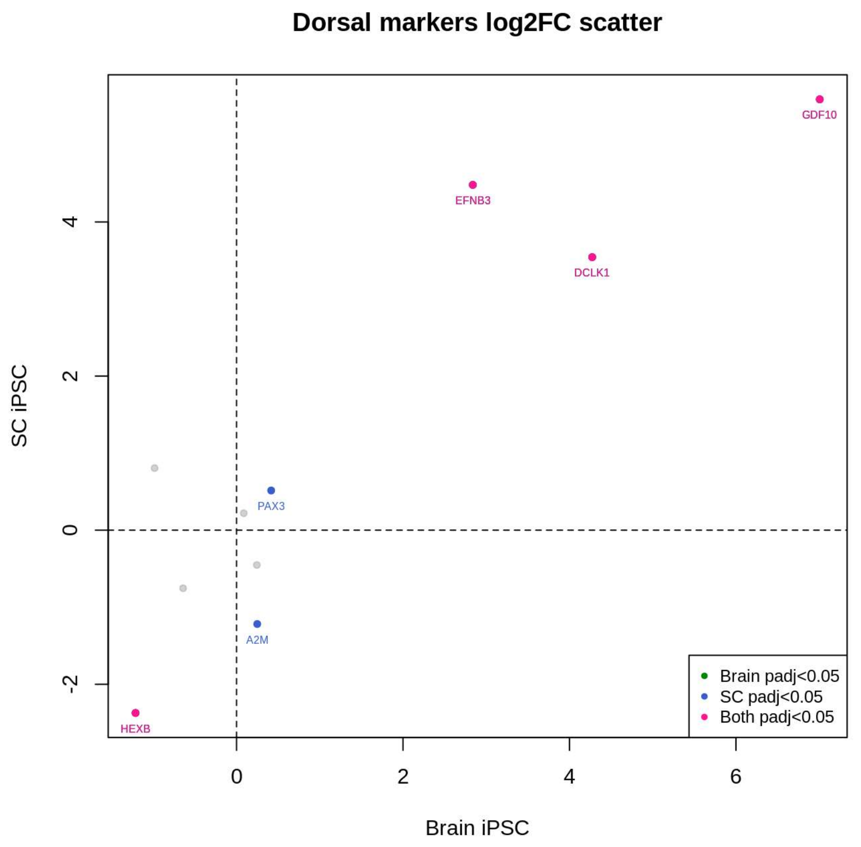

Ventral genes

```
## [1] "Found data for 11 of 12 genes in the list"
```

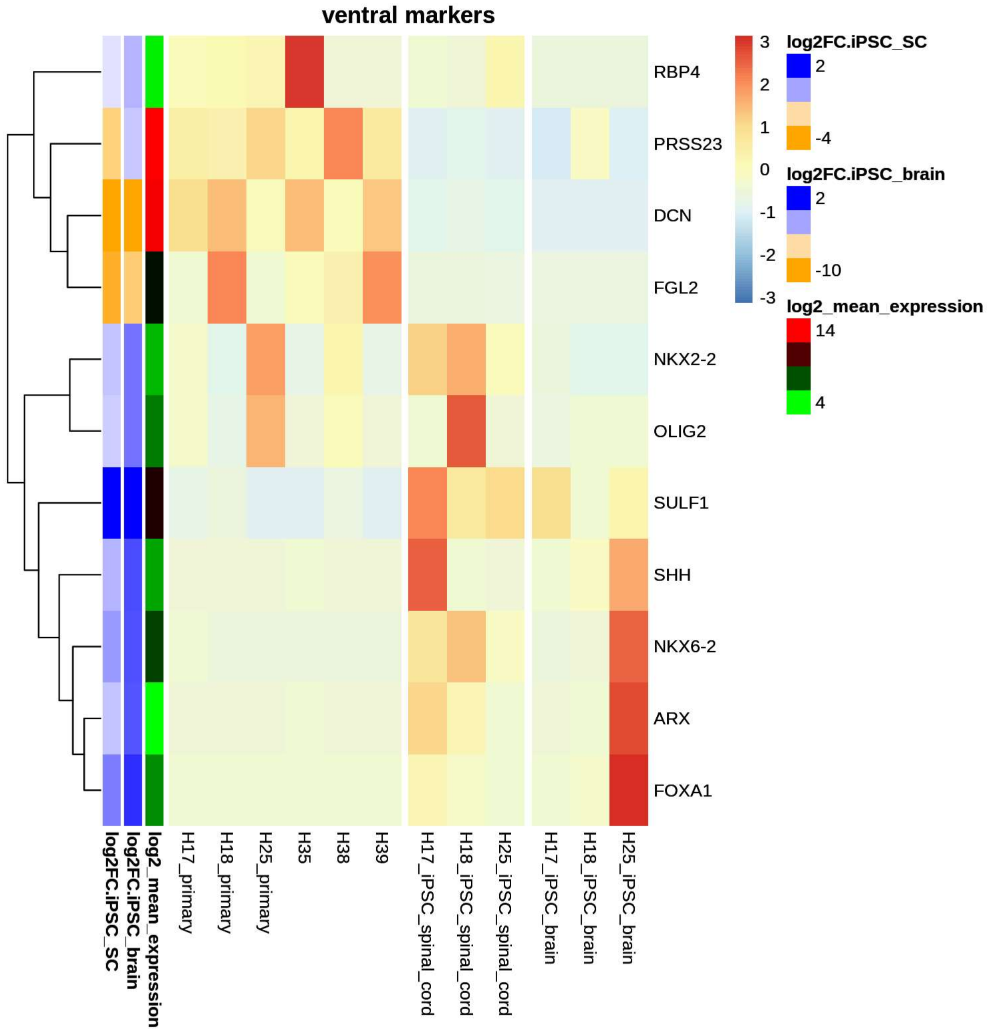

ventral markers
RBP4
PRSS23
DCN
FGL2
NKX2-2
OLIG2
SULF1
SHH
NKX6-2
ARX
FOXA1
log2FC.iPSC_SC
log2FC.iPSC_brain
log2_mean_expression
H17_primary
H18_primary
H25_primary
H35
H38
H39
H17_iPSC_spinal_cord
H18_iPSC_spinal_cord
H25_iPSC_spinal_cord
H17_iPSC_brain
H18_iPSC_brain
H25_iPSC_brain
3
2
1
0
-1
-2
-3
log2FC.iPSC_SC
2
-4
log2FC.iPSC_brain
2
-10
log2_mean_expression
14
4

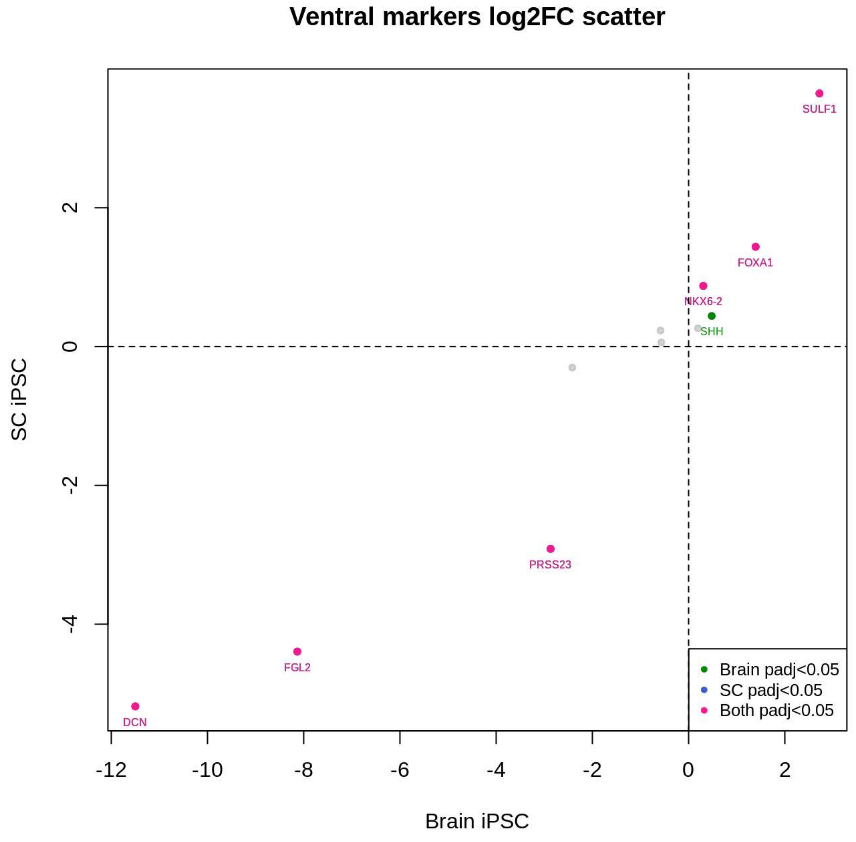

Ventral markers log2FC scatter
SC iPSC
Brain iPSC
SULF1
FOXA1
NKX6-2
SHH
PRSS23
FGL2
DCN
Brain padj<0.05
SC padj<0.05
Both padj<0.05

More heatmaps with MSigDb gene sets

Enrichment analysis showed various enriched classes, so we can plot heatmaps for the genes in these gene sets.

Reactome Interferon Alpha/Beta signalling was enriched, so let's try that:

```
##       [,1]
##  [1,] "302"
##  [2,] "303"
##  [3,] "1280"
##  [4,] "1816"
##  [5,] "2420"
##  [6,] "2492"
##  [7,] "2521"
##  [8,] "2845"
##  [9,] "2923"
## [10,] "2977"
## [11,] "11200"
## [12,] "17033"
## [13,] "17903"
## [14,] "17904"
## [15,] "18533"
## [16,] "18771"
## [17,] "18801"
## [18,] "19074"
## [19,] "19108"
## [20,] "19296"
## [21,] "20549"
## [22,] "21530"
## [23,] "21753"
## [24,] "21758"
## [25,] "26844"
## [26,] "26845"
## [27,] "26846"
## [28,] "26900"
## [29,] "26901"
## [30,] "26902"
## [31,] "26903"
## [32,] "26932"
## [33,] "26933"
## [34,] "26934"
## [35,] "26962"
## [36,] "26963"
## [37,] "26964"
## [38,] "26989"
## [39,] "26990"
## [40,] "26991"
## [41,] "27506"
## [42,] "27507"
## [43,] "27508"
## [44,] "27509"
## [45,] "27510"
## [46,] "27511"
## [47,] "27512"
## [48,] "27806"
## [49,] "27807"
## [50,] "27808"
## [51,] "27809"
## [52,] "31386"
## [53,] "31387"
## [54,] "31388"
## [55,] "31389"
## [56,] "31390"
## [57,] "31391"
## [58,] "31392"
## [59,] "31393"
## [60,] "31394"
## [61,] "31395"
## [62,] "31396"
## [63,] "32699"
## [64,] "32710"
## [65,] "40837"
## [66,] "40838"
## [67,] "40839"
## [68,] "40974"
## [69,] "40975"
## [70,] "42255"
## [71,] "42256"
## [72,] "42257"
##       [,2]
##  [1,] "BLANCO_MELO_BETA_INTERFERON_TREATED_BRONCHIAL_EPITHELIAL_CELLS_UP"
##  [2,] "BLANCO_MELO_BETA_INTERFERON_TREATED_BRONCHIAL_EPITHELIAL_CELLS_DN"
##  [3,] "NATSUME_RESPONSE_TO_INTERFERON_BETA_DN"
##  [4,] "CHIANG_LIVER_CANCER_SUBCLASS_INTERFERON_DN"
```

```
##  [5,] "WP_TYPE_II_INTERFERON_SIGNALING_IFNG"
##  [6,] "WP_INTERFERON_TYPE_I_SIGNALING_PATHWAYS"
##  [7,] "WP_TYPE_III_INTERFERON_SIGNALING"
##  [8,] "WP_OVERVIEW_OF_INTERFERONSMEDIATED_SIGNALING_PATHWAY"
##  [9,] "WP_TYPE_I_INTERFERON_INDUCTION_AND_SIGNALING_DURING_SARSCOV2_INFECTION"
## [10,] "WP_HOSTPATHOGEN_INTERACTION_OF_HUMAN_CORONA_VIRUSES_INTERFERON_INDUCTION"
## [11,] "HP_ABNORMAL_SERUM_INTERFERON_LEVEL"
## [12,] "RICHERT_PBMC_HIV_LIPO_5_AGE_37_48YO_STIMULATED_VS_UNSTIMULATED_14W_INTERFERON_SUBSET_UP"
## [13,] "HALLMARK_INTERFERON_ALPHA_RESPONSE"
## [14,] "HALLMARK_INTERFERON_GAMMA_RESPONSE"
## [15,] "EINAV_INTERFERON_SIGNATURE_IN_CANCER"
## [16,] "NATSUME_RESPONSE_TO_INTERFERON_BETA_UP"
## [17,] "BROWNE_INTERFERON_RESPONSIVE_GENES"
## [18,] "ZHANG_INTERFERON_RESPONSE"
## [19,] "CHIANG_LIVER_CANCER_SUBCLASS_INTERFERON_UP"
## [20,] "BOSCO_INTERFERON_INDUCED_ANTIVIRAL_MODULE"
## [21,] "REACTOME_DDX58_IFIH1_MEDIATED_INDUCTION_OF_INTERFERON_ALPHA_BETA"
## [22,] "REACTOME_INTERFERON_GAMMA_SIGNALING"
## [23,] "REACTOME_INTERFERON_ALPHA_BETA_SIGNALING"
## [24,] "REACTOME_INTERFERON_SIGNALING"
## [25,] "GOBP_REGULATION_OF_TYPE_I_INTERFERON_PRODUCTION"
## [26,] "GOBP_NEGATIVE_REGULATION_OF_TYPE_I_INTERFERON_PRODUCTION"
## [27,] "GOBP_POSITIVE_REGULATION_OF_TYPE_I_INTERFERON_PRODUCTION"
## [28,] "GOBP_TYPE_I_INTERFERON_PRODUCTION"
## [29,] "GOBP_INTERFERON-ALPHA_PRODUCTION"
## [30,] "GOBP_INTERFERON-BETA_PRODUCTION"
## [31,] "GOBP_INTERFERON-GAMMA_PRODUCTION"
## [32,] "GOBP_REGULATION_OF_INTERFERON-ALPHA_PRODUCTION"
## [33,] "GOBP_REGULATION_OF_INTERFERON-BETA_PRODUCTION"
## [34,] "GOBP_REGULATION_OF_INTERFERON-GAMMA_PRODUCTION"
## [35,] "GOBP_NEGATIVE_REGULATION_OF_INTERFERON-ALPHA_PRODUCTION"
## [36,] "GOBP_NEGATIVE_REGULATION_OF_INTERFERON-BETA_PRODUCTION"
## [37,] "GOBP_NEGATIVE_REGULATION_OF_INTERFERON-GAMMA_PRODUCTION"
## [38,] "GOBP_POSITIVE_REGULATION_OF_INTERFERON-ALPHA_PRODUCTION"
## [39,] "GOBP_POSITIVE_REGULATION_OF_INTERFERON-BETA_PRODUCTION"
## [40,] "GOBP_POSITIVE_REGULATION_OF_INTERFERON-GAMMA_PRODUCTION"
## [41,] "GOBP_RESPONSE_TO_TYPE_I_INTERFERON"
## [42,] "GOBP_RESPONSE_TO_INTERFERON-GAMMA"
## [43,] "GOBP_RESPONSE_TO_TYPE_III_INTERFERON"
## [44,] "GOBP_TYPE_III_INTERFERON_PRODUCTION"
## [45,] "GOBP_REGULATION_OF_TYPE_III_INTERFERON_PRODUCTION"
## [46,] "GOBP_NEGATIVE_REGULATION_OF_TYPE_III_INTERFERON_PRODUCTION"
## [47,] "GOBP_POSITIVE_REGULATION_OF_TYPE_III_INTERFERON_PRODUCTION"
## [48,] "GOBP_RESPONSE_TO_INTERFERON-ALPHA"
## [49,] "GOBP_RESPONSE_TO_INTERFERON-BETA"
## [50,] "GOBP_CELLULAR_RESPONSE_TO_INTERFERON-ALPHA"
## [51,] "GOBP_CELLULAR_RESPONSE_TO_INTERFERON-BETA"
## [52,] "GOBP_REGULATION_OF_RESPONSE_TO_INTERFERON-GAMMA"
## [53,] "GOBP_NEGATIVE_REGULATION_OF_RESPONSE_TO_INTERFERON-GAMMA"
## [54,] "GOBP_POSITIVE_REGULATION_OF_RESPONSE_TO_INTERFERON-GAMMA"
## [55,] "GOBP_INTERFERON-GAMMA-MEDIATED_SIGNALING_PATHWAY"
## [56,] "GOBP_REGULATION_OF_INTERFERON-GAMMA-MEDIATED_SIGNALING_PATHWAY"
## [57,] "GOBP_POSITIVE_REGULATION_OF_INTERFERON-GAMMA-MEDIATED_SIGNALING_PATHWAY"
## [58,] "GOBP_NEGATIVE_REGULATION_OF_INTERFERON-GAMMA-MEDIATED_SIGNALING_PATHWAY"
## [59,] "GOBP_TYPE_I_INTERFERON_SIGNALING_PATHWAY"
## [60,] "GOBP_REGULATION_OF_TYPE_I_INTERFERON-MEDIATED_SIGNALING_PATHWAY"
## [61,] "GOBP_NEGATIVE_REGULATION_OF_TYPE_I_INTERFERON-MEDIATED_SIGNALING_PATHWAY"
## [62,] "GOBP_POSITIVE_REGULATION_OF_TYPE_I_INTERFERON-MEDIATED_SIGNALING_PATHWAY"
## [63,] "GOBP_CELLULAR_RESPONSE_TO_INTERFERON-GAMMA"
## [64,] "GOBP_CELLULAR_RESPONSE_TO_TYPE_I_INTERFERON"
## [65,] "GOMF_INTERFERON_RECEPTOR_ACTIVITY"
## [66,] "GOMF_TYPE_I_INTERFERON_RECEPTOR_ACTIVITY"
## [67,] "GOMF_INTERFERON-GAMMA_RECEPTOR_ACTIVITY"
## [68,] "GOMF_TYPE_I_INTERFERON_RECEPTOR_BINDING"
## [69,] "GOMF_INTERFERON-GAMMA_RECEPTOR_BINDING"
## [70,] "GOMF_INTERFERON_BINDING"
## [71,] "GOMF_TYPE_I_INTERFERON_BINDING"
## [72,] "GOMF_INTERFERON-GAMMA_BINDING"
```

Want to use REACTOME_INTERFERON_ALPHA_BETA_SIGNALING, REACTOME_INTERFERON_GAMMA_SIGNALING, REACTOME_INTERFERON_SIGNALING

REACTOME_INTERFERON_ALPHA_BETA_SIGNALING genes

REACTOME_INTERFERON_ALPHA_BETA_SIGNALING is gene set 21753.

```
## [1] "Found data for 59 of 73 genes in the list"
```

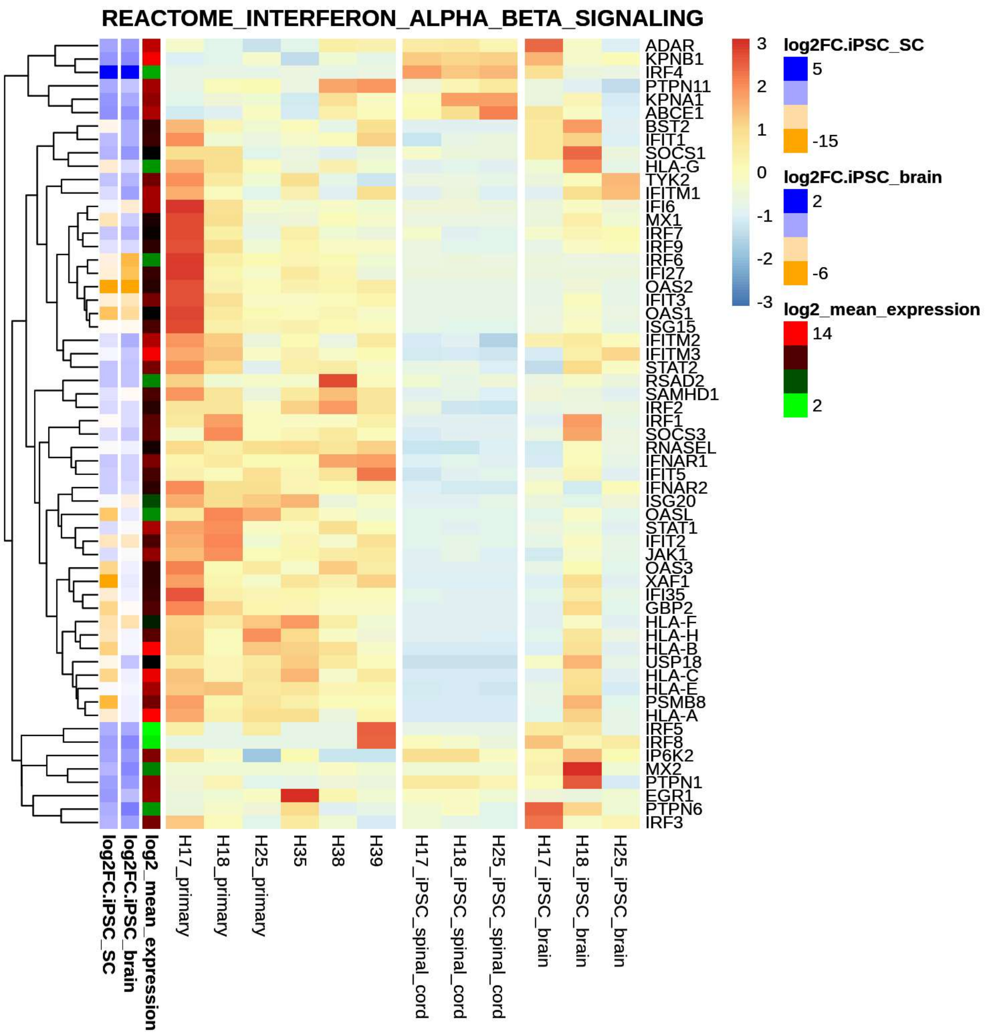
REACTOME_INTERFERON_ALPHA_BETA_SIGNALING
ADAR
KPNB1
IRF4
PTPN11
KPNA1
ABCE1
BST2
IFIT1
SOCS1
HLA-G
TYK2
IFITM1
IFI6
MX1
IRF7
IRF9
IRF6
IFI27
OAS2
IFIT3
OAS1
ISG15
IFITM2
IFITM3
STAT2
RSAD2
SAMHD1
IRF2
IRF1
SOCS3
RNASEL
IFNAR1
IFIT5
IFNAR2
ISG20
OASL
STAT1
IFIT2
JAK1
OAS3
XAF1
IFI35
GBP2
HLA-F
HLA-H
HLA-B
USP18
HLA-C
HLA-E
PSMB8
HLA-A
IRF5
IRF8
IP6K2
MX2
PTPN1
EGR1
PTPN6
IRF3
log2FC.iPSC_SC
log2FC.iPSC_brain
log2_mean_expression
H17_primary
H18_primary
H25_primary
H35
H38
H39
H17_iPSC_spinal_cord
H18_iPSC_spinal_cord
H25_iPSC_spinal_cord
H17_iPSC_brain
H18_iPSC_brain
H25_iPSC_brain
3
2
1
0
-1
-2
-3
log2FC.iPSC_SC
5
-15
log2FC.iPSC_brain
2
-6
log2_mean_expression
14
2

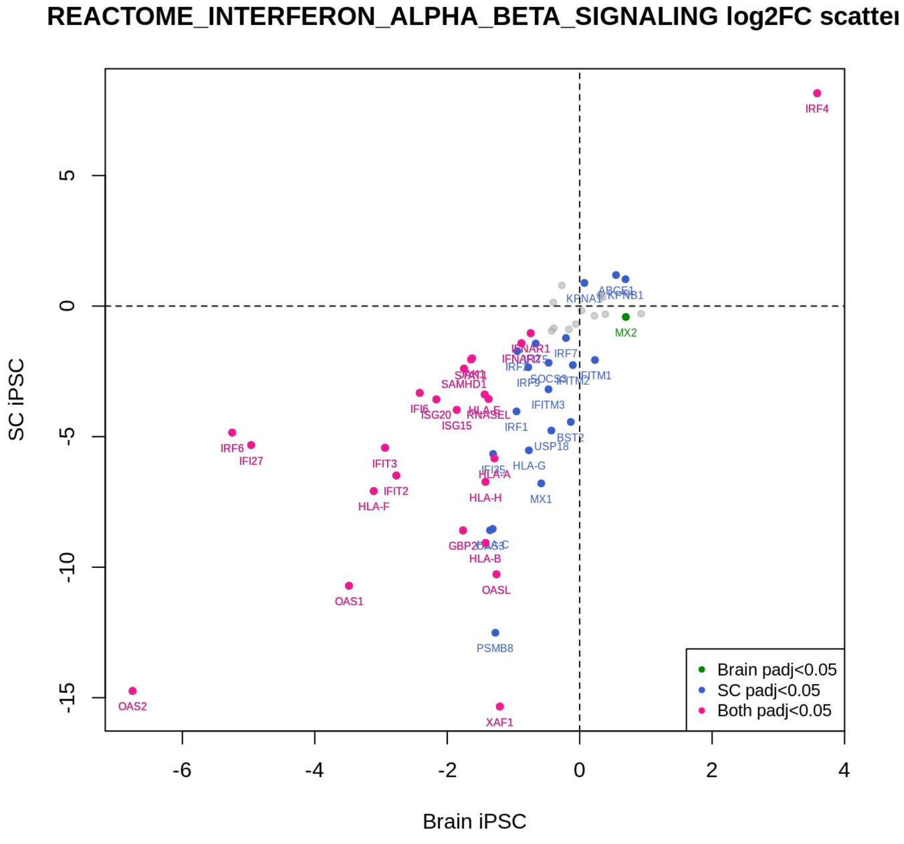
REACTOME_INTERFERON_ALPHA_BETA_SIGNALING log2FC scatter
SC iPSC
Brain iPSC
5
0
-5
-10
-15
-6
-4
-2
0
2
4
IRF4
KPNA1
ABCE1
KPNB1
MX2
IRF7
IFITM1
IFITM2
IFITM3
SOCS3
IRF9
IRF1
BST2
USP18
HLA-G
MX1
IFNAR1
IFNAR2
STAT1
SAMHD1
IFI6
ISG20
ISG15
RNASEL
IRF6
IFI27
IFIT3
IFIT2
HLA-F
HLA-A
HLA-H
GBP2
HLA-B
OASL
OAS1
PSMB8
OAS2
XAF1
Brain padj<0.05
SC padj<0.05
Both padj<0.05

REACTOME_INTERFERON_GAMMA_SIGNALING genes

REACTOME_INTERFERON_GAMMA_SIGNALING is gene set 21530.

```
## [1] "Found data for 83 of 93 genes in the list"
```

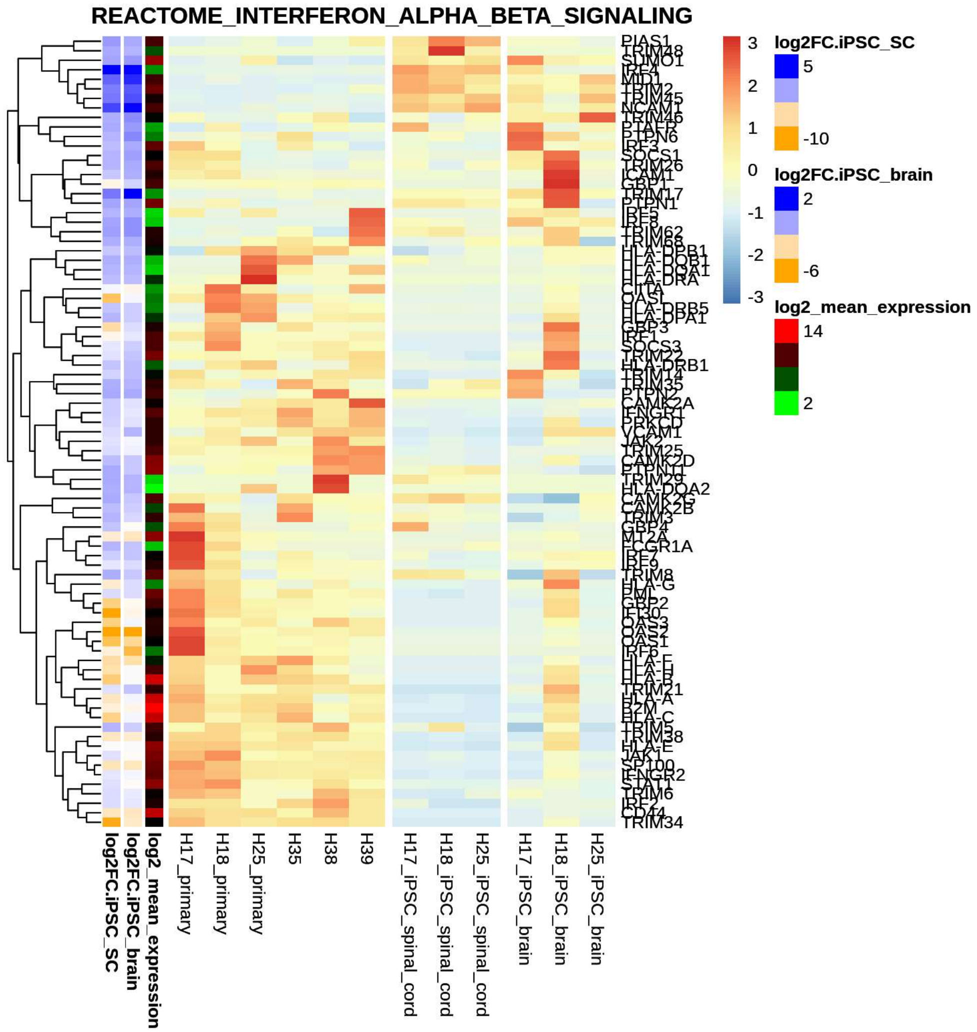

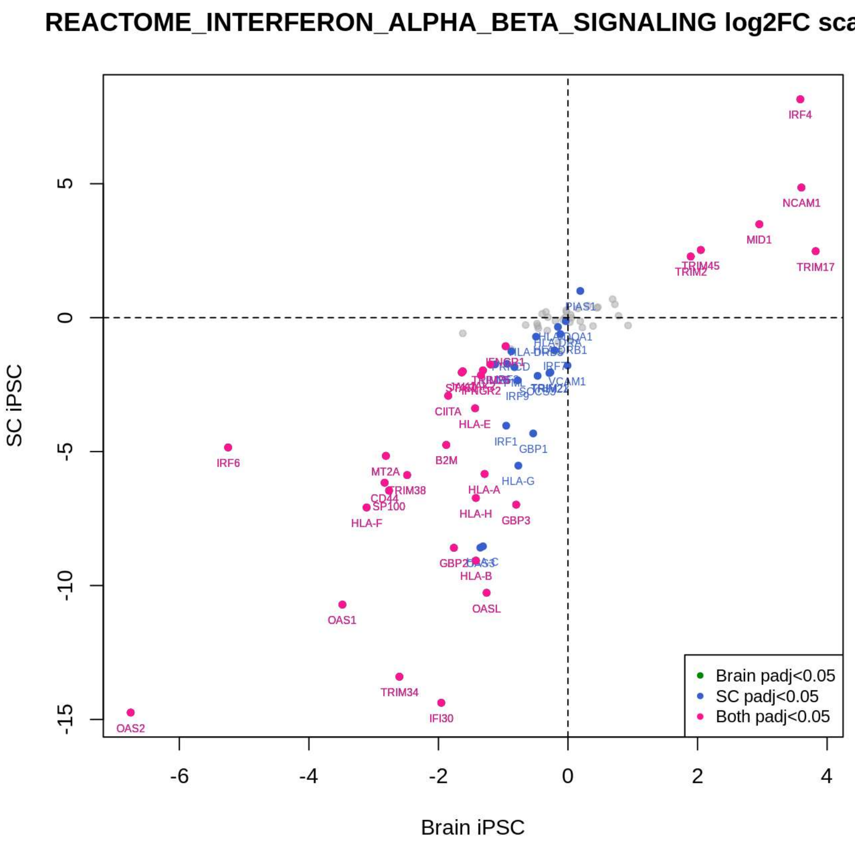

File generation date and environment

File generation timestamp: Thu Jun 30 13:19:29 2022

Name	Value	Section
Version	R version 4.1.2 (2021-11-01)	R
GSEABase	1.56.0 NA NA 2021-10-26	Packages
graph	1.72.0 NA NA 2021-10-26	Packages
annotate	1.72.0 NA NA 2021-10-26	Packages
XML	3.99-0.10 CRAN CRAN 2022-06-09 22:40:12 UTC	Packages
AnnotationDbi	1.56.2 NA NA 2021-11-09	Packages
rgl	0.109.2 CRAN CRAN 2022-06-10 06:40:07 UTC	Packages
scatterplot3d	0.3-41 CRAN CRAN 2018-02-13	Packages
genefilter	1.76.0 NA NA 2021-10-26	Packages
msigdb	1.2.0 NA NA 2021-10-30	Packages
forcats	0.5.1 CRAN CRAN 2021-01-27 19:50:02 UTC	Packages
stringr	1.4.0 CRAN CRAN 2019-02-10 03:40:03 UTC	Packages
dplyr	1.0.9 CRAN CRAN 2022-04-28 13:30:02 UTC	Packages
purrr	0.3.4 CRAN CRAN 2020-04-17 12:10:07 UTC	Packages
readr	2.1.2 CRAN CRAN 2022-01-30 22:30:02 UTC	Packages
tidyr	1.2.0 CRAN CRAN 2022-02-01 08:40:02 UTC	Packages
tibble	3.1.7 CRAN CRAN 2022-05-03 07:00:06 UTC	Packages
tidyverse	1.3.1 CRAN CRAN 2021-04-15 13:20:02 UTC	Packages
pheatmap	1.0.12 CRAN CRAN 2018-12-26	Packages
EnhancedVolcano	1.12.0 NA NA 2021-10-26	Packages
VennDiagram	1.7.3 CRAN CRAN 2022-04-11	Packages
futile.logger	1.4.3 CRAN CRAN 2016-07-10	Packages
kableExtra	1.3.4 CRAN CRAN 2021-02-20 05:50:07 UTC	Packages
jsonlite	1.8.0 CRAN CRAN 2022-02-22 11:20:02 UTC	Packages
BiocParallel	1.28.3 NA NA 2021-12-09	Packages
apeglm	1.16.0 NA NA 2021-10-26	Packages

Name	Value	Section
tximport	1.22.0 NA NA 2021-10-26	Packages
rmarkdown	2.14 CRAN CRAN 2022-04-25 19:20:02 UTC	Packages
envDocument	2.4.1 CRAN CRAN 2019-08-08	Packages
knitr	1.39 CRAN CRAN 2022-04-26 23:30:05 UTC	Packages
gsubfn	0.7 CRAN CRAN 2018-03-15	Packages
proto	1.0.0 CRAN CRAN 2016-10-29 00:23:07	Packages
rtracklayer	1.54.0 NA NA 2021-10-26	Packages
biomaRt	2.50.3 NA NA 2022-02-03	Packages
ggrepel	0.9.1 CRAN CRAN 2021-01-15 22:00:02 UTC	Packages
ggplot2	3.3.6 CRAN CRAN 2022-05-03 07:00:14 UTC	Packages
RColorBrewer	1.1-3 CRAN CRAN 2022-04-03	Packages
DESeq2	1.34.0 NA NA 2021-10-26	Packages
SummarizedExperiment	1.24.0 NA NA 2021-10-26	Packages
Biobase	2.54.0 NA NA 2021-10-26	Packages
MatrixGenerics	1.6.0 NA NA 2021-10-26	Packages
matrixStats	0.62.0 CRAN CRAN 2022-04-19 12:12:33 UTC	Packages
GenomicRanges	1.46.1 NA NA 2021-11-18	Packages
GenomeInfoDb	1.30.1 NA NA 2022-01-30	Packages
IRanges	2.28.0 NA NA 2021-10-26	Packages
S4Vectors	0.32.4 NA NA 2022-03-24	Packages
BiocGenerics	0.40.0 NA NA 2021-10-26	Packages
utf8	1.2.2 CRAN CRAN 2021-07-24 15:00:02 UTC	Packages
tidyselect	1.1.2 CRAN CRAN 2022-02-21 16:00:02 UTC	Packages
RSQLite	2.2.14 CRAN CRAN 2022-05-05	Packages
htmlwidgets	1.5.4 CRAN CRAN 2021-09-08 14:00:02 UTC	Packages
munsell	0.5.0 CRAN CRAN 2018-06-12 04:29:06 UTC	Packages
withr	2.5.0 CRAN CRAN 2022-03-03 21:50:02 UTC	Packages
colorspace	2.0-3 CRAN CRAN 2022-02-19	Packages
filelock	1.0.2 CRAN CRAN 2018-10-05 10:30:12 UTC	Packages
ggalt	0.4.0 CRAN CRAN 2017-02-15 18:16:00	Packages
highr	0.9 CRAN CRAN 2021-04-16 21:50:05 UTC	Packages
rstudioapi	0.13 CRAN CRAN 2020-11-12 21:50:02 UTC	Packages
Rttf2pt1	1.3.10 CRAN CRAN 2022-02-07 03:10:02 UTC	Packages
labeling	0.4.2 CRAN CRAN 2020-10-15	Packages
bbmle	1.0.25 CRAN CRAN 2022-05-11 08:20:02 UTC	Packages
GenomeInfoDbData	1.2.7 NA NA NA	Packages
bit64	4.0.5 CRAN CRAN 2020-08-29	Packages
farver	2.1.0 CRAN CRAN 2021-02-28 19:30:02 UTC	Packages
coda	0.19-4 CRAN CRAN 2020-09-30	Packages
vctrs	0.4.1 CRAN CRAN 2022-04-13 10:30:02 UTC	Packages
generics	0.1.2 CRAN CRAN 2022-01-31 23:30:02 UTC	Packages
lambda.r	1.2.4 CRAN CRAN 2019-09-15	Packages
xfun	0.31 CRAN CRAN 2022-05-10 20:30:02 UTC	Packages
BiocFileCache	2.2.1 NA NA 2022-01-23	Packages
R6	2.5.1 CRAN CRAN 2021-08-19 14:00:05 UTC	Packages
ggbeeswarm	0.6.0 CRAN CRAN 2017-08-03	Packages
locfit	1.5-9.5 CRAN CRAN 2022-03-01	Packages
bitops	1.0-7 CRAN CRAN 2021-04-13	Packages
cachem	1.0.6 CRAN CRAN 2021-08-19 21:30:02 UTC	Packages
DelayedArray	0.20.0 NA NA 2021-10-26	Packages
assertthat	0.2.1 CRAN CRAN 2019-03-21 14:53:46 UTC	Packages
promises	1.2.0.1 CRAN CRAN 2021-02-11 19:00:02 UTC	Packages
BiocIO	1.4.0 NA NA 2021-10-26	Packages
scales	1.2.0 CRAN CRAN 2022-04-13 22:40:02 UTC	Packages
vroom	1.5.7 CRAN CRAN 2021-11-30 14:20:06 UTC	Packages
beeswarm	0.4.0 CRAN CRAN 2021-05-07	Packages
gtable	0.3.0 CRAN CRAN 2019-03-25 19:50:02 UTC	Packages

Name	Value	Section
ash	1.0-15 CRAN CRAN 2015-08-27	Packages
rlang	1.0.3 CRAN CRAN 2022-06-27 18:50:02 UTC	Packages
systemfonts	1.0.4 CRAN CRAN 2022-02-11 12:50:02 UTC	Packages
splines	4.1.2 NA NA NA	Packages
extrafontdb	1.0 CRAN CRAN 2012-06-11 15:40:11	Packages
broom	0.8.0 CRAN CRAN 2022-04-13 15:02:34 UTC	Packages
BiocManager	1.30.18 CRAN CRAN 2022-05-18 21:20:02 UTC	Packages
yaml	2.3.5 CRAN CRAN 2022-01-24	Packages
modelr	0.1.8 CRAN CRAN 2020-05-19 20:00:35 UTC	Packages
backports	1.4.1 CRAN CRAN 2021-12-13 11:30:02 UTC	Packages
httpuv	1.6.5 CRAN CRAN 2022-01-05 00:40:11 UTC	Packages
extrafont	0.18 CRAN CRAN 2022-04-12 16:32:29 UTC	Packages
tools	4.1.2 NA NA NA	Packages
tcltk	4.1.2 NA NA NA	Packages
ellipsis	0.3.2 CRAN CRAN 2021-04-29 12:40:02 UTC	Packages
jquerylib	0.1.4 CRAN CRAN 2021-04-26 17:10:02 UTC	Packages
Rcpp	1.0.8.3 CRAN CRAN 2022-03-13	Packages
plyr	1.8.7 CRAN CRAN 2022-03-24 21:50:02 UTC	Packages
base64enc	0.1-3 CRAN CRAN 2015-07-28 08:03:37	Packages
progress	1.2.2 CRAN CRAN 2019-05-16 21:30:03 UTC	Packages
zlibbioc	1.40.0 NA NA 2021-10-26	Packages
RCurl	1.98-1.7 CRAN CRAN 2022-06-09 09:26:30 UTC	Packages
prettyunits	1.1.1 CRAN CRAN 2020-01-24 06:50:07 UTC	Packages
haven	2.5.0 CRAN CRAN 2022-04-15 16:02:30 UTC	Packages
fs	1.5.2 CRAN CRAN 2021-12-08 23:00:08 UTC	Packages
magrittr	2.0.3 CRAN CRAN 2022-03-30 07:30:09 UTC	Packages
futile.options	1.0.1 CRAN CRAN 2018-04-20	Packages
reprex	2.0.1 CRAN CRAN 2021-08-05 04:20:10 UTC	Packages
mvtnorm	1.1-3 CRAN CRAN 2021-10-05	Packages
mime	0.12 CRAN CRAN 2021-09-28 05:00:05 UTC	Packages
hms	1.1.1 CRAN CRAN 2021-09-26	Packages
evaluate	0.15 CRAN CRAN 2022-02-18 17:20:02 UTC	Packages
xtable	1.8-4 CRAN CRAN 2019-04-08	Packages
emdbook	1.3.12 CRAN CRAN 2020-02-19 16:50:02 UTC	Packages
readxl	1.4.0 CRAN CRAN 2022-03-28 18:30:02 UTC	Packages
compiler	4.1.2 NA NA NA	Packages
bdsmatrix	1.3-6 CRAN CRAN 2022-06-03	Packages
maps	3.4.0 CRAN CRAN 2021-09-25	Packages
KernSmooth	2.23-20 CRAN CRAN 2021-05-03	Packages
crayon	1.5.1 CRAN CRAN 2022-03-26 17:30:05 UTC	Packages
htmltools	0.5.2 CRAN CRAN 2021-08-25 13:50:02 UTC	Packages
later	1.3.0 CRAN CRAN 2021-08-18 16:30:06 UTC	Packages
tzdb	0.3.0 CRAN CRAN 2022-03-28 15:00:02 UTC	Packages
geneplotter	1.72.0 NA NA 2021-10-26	Packages
lubridate	1.8.0 CRAN CRAN 2021-10-07 15:20:02 UTC	Packages
DBI	1.1.3 CRAN CRAN 2022-06-18	Packages
formatR	1.12 CRAN CRAN 2022-03-31 20:20:02 UTC	Packages
ExperimentHub	2.2.1 NA NA 2022-01-23	Packages
dbplyr	2.2.1 CRAN CRAN 2022-06-27 15:20:07 UTC	Packages
proj4	1.0-11 CRAN CRAN 2022-01-31 13:39:39 UTC	Packages
MASS	7.3-54 CRAN CRAN 2021-04-17	Packages
rappdirs	0.3.3 CRAN CRAN 2021-01-31 05:40:02 UTC	Packages
Matrix	1.3-4 CRAN CRAN 2021-05-24	Packages
cli	3.3.0 CRAN CRAN 2022-04-25 10:00:06 UTC	Packages
parallel	4.1.2 NA NA NA	Packages
pkgconfig	2.0.3 CRAN CRAN 2019-09-22 09:20:02 UTC	Packages
GenomicAlignments	1.30.0 NA NA 2021-10-26	Packages

Name	Value	Section
numDeriv	2016.8-1.1 CRAN CRAN 2019-06-06 09:51:09 UTC	Packages
xml2	1.3.3 CRAN CRAN 2021-11-30 14:40:02 UTC	Packages
svglite	2.1.0 CRAN CRAN 2022-02-03 09:00:02 UTC	Packages
vipor	0.4.5 CRAN CRAN 2017-03-22	Packages
bslib	0.3.1 CRAN CRAN 2021-10-06 15:10:05 UTC	Packages
webshot	0.5.3 CRAN CRAN 2022-04-14 12:40:02 UTC	Packages
XVector	0.34.0 NA NA 2021-10-26	Packages
rvest	1.0.2 CRAN CRAN 2021-10-16 23:30:07 UTC	Packages
digest	0.6.29 CRAN CRAN 2021-11-30	Packages
Biostrings	2.62.0 NA NA 2021-10-26	Packages
cellranger	1.1.0 CRAN CRAN 2016-07-27 03:17:48	Packages
restfulr	0.0.15 CRAN CRAN 2022-06-16 09:30:02 UTC	Packages
curl	4.3.2 CRAN CRAN 2021-06-23 07:00:06 UTC	Packages
shiny	1.7.1 CRAN CRAN 2021-10-02 04:30:02 UTC	Packages
Rsamtools	2.10.0 NA NA 2021-10-26	Packages
rjson	0.2.21 CRAN CRAN 2022-01-06	Packages
lifecycle	1.0.1 CRAN CRAN 2021-09-24 15:30:02 UTC	Packages
viridisLite	0.4.0 CRAN CRAN 2021-04-13 15:00:02 UTC	Packages
fansi	1.0.3 CRAN CRAN 2022-03-24 07:50:02 UTC	Packages
pillar	1.7.0 CRAN CRAN 2022-02-01 08:30:02 UTC	Packages
lattice	0.20-45 CRAN CRAN 2021-09-18	Packages
ggrastr	1.0.1 CRAN CRAN 2021-12-08 07:30:03 UTC	Packages
KEGGREST	1.34.0 NA NA 2021-10-26	Packages
fastmap	1.1.0 CRAN CRAN 2021-01-25 21:00:02 UTC	Packages
httr	1.4.3 CRAN CRAN 2022-05-04 00:20:02 UTC	Packages
survival	3.2-13 CRAN CRAN 2021-08-23	Packages
interactiveDisplayBase	1.32.0 NA NA 2021-07-30	Packages
glue	1.6.2 CRAN CRAN 2022-02-24 07:50:20 UTC	Packages
png	0.1-7 CRAN CRAN 2013-12-03 22:25:05	Packages
BiocVersion	3.14.0 NA NA 2021-05-19	Packages
bit	4.0.4 CRAN CRAN 2020-08-03	Packages
stringi	1.7.6 CRAN CRAN 2021-11-29	Packages
sass	0.4.1 CRAN CRAN 2022-03-23 17:10:02 UTC	Packages
blob	1.2.3 CRAN CRAN 2022-04-10 06:22:28 UTC	Packages
AnnotationHub	3.2.2 NA NA 2022-03-01	Packages
memoise	2.0.1 CRAN CRAN 2021-11-26 16:11:10 UTC	Packages
Path	/global/online/ohri/ohri1/projects/ahmad_galuta/human_spinal_cord_SC_iPSC/analysis/DESeq2/analysis_report.Rmd	Script
Modified	2022-06-30 13:12:04	Script
All Attributes	Not Available	Git

Appendix: Dispersion plots

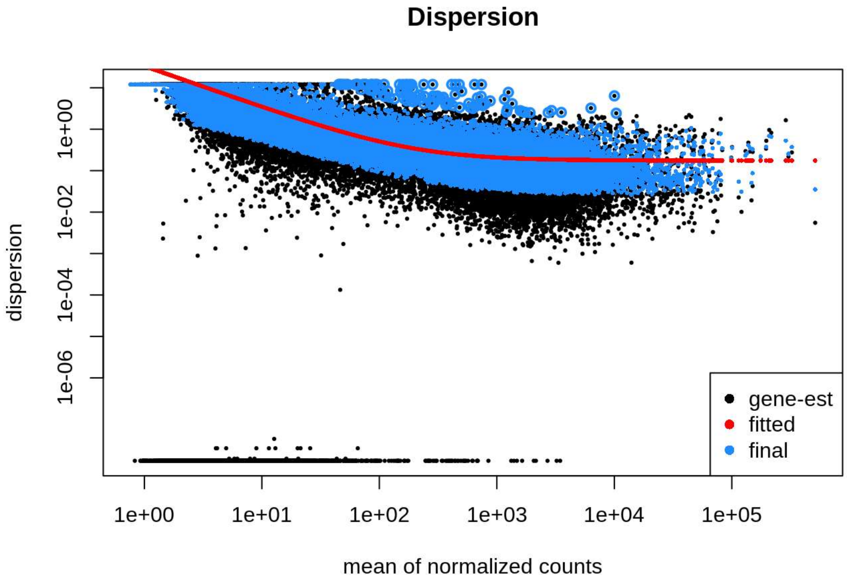

Appendix: MA plots

Appendix: Count scatter plots
