# Supplementary material for: Transcriptomic and Functional Landscape of Adult Human Spinal Cord NSPCs Compared to iPSC-Derived Neural Progenitor Cells
Source: Cells. 2025 Jan 7;14(2):64. doi: 10.3390/cells14020064 (PMC11763936; doi:10.3390/cells14020064)
Supplement: Supplementary file 1 [file cells-14-00064-s001.zip › cells-3390501-supplementary.pdf]

Supplementary Figures

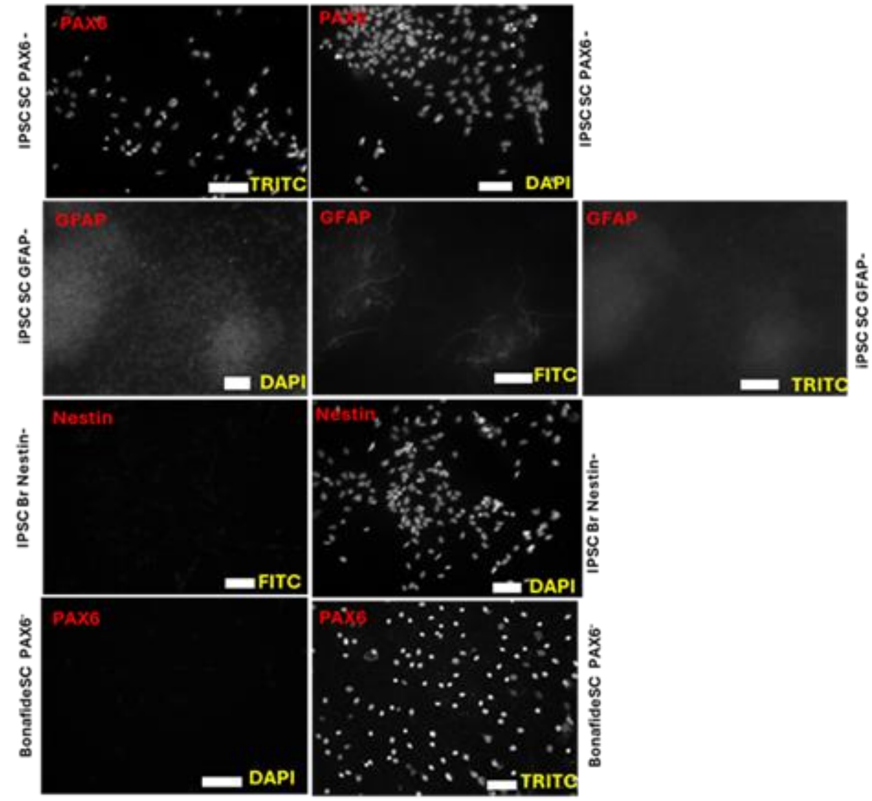

Supplementary Figure S1: **Negative Staining Validation for Neural Stem/Progenitor Cell Markers in iPSC-Derived and Bona Fide NSPCs.** This figure demonstrates the negative staining results for key neural stem/progenitor cell markers (Pax6, GFAP, and Nestin) across iPSC-derived NSPCs (iPSC-Br and iPSC-SC) and bona fide NSPCs. The absence of GFAP in iPSC-SC NSPCs and the absence of Nestin in iPSC-Br NSPCs validate the specificity of the staining protocol.

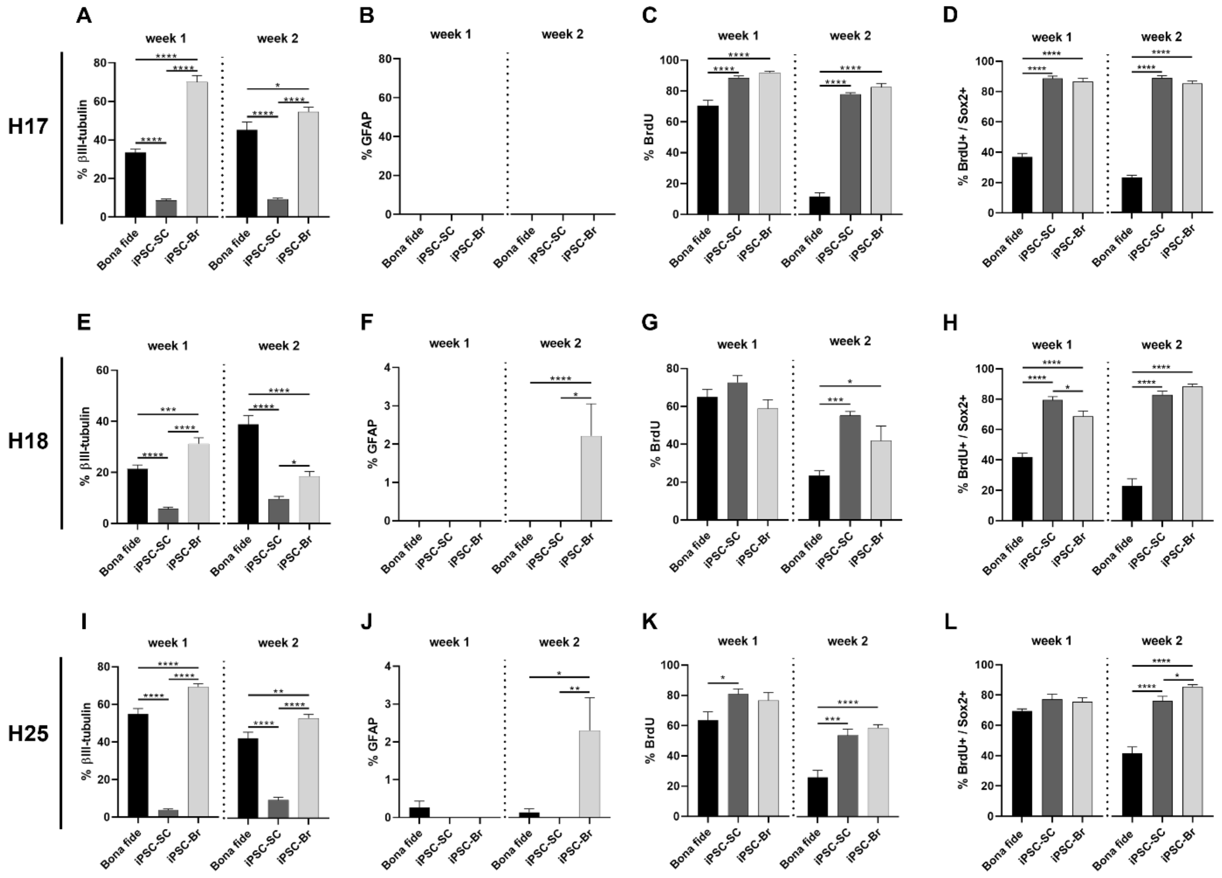

Supplementary Figure S2: Donor-Specific Differentiation and Proliferation Profiles of Bona Fide and iPSC-Derived NSPCs.

Differentiation and proliferation profiles of bona fide NSPCs, iPSC-SC NSPCs, and iPSC-Br NSPCs are presented for technical replicates across three donors (H17, H18, H25). (A, E, I) Neuronal differentiation, measured as the percentage of β-III tubulin+ cells, shows consistently higher levels in iPSC-Br NSPCs compared to bona fide and iPSC-SC NSPCs. (B, F, J) Astrocytic differentiation, indicated by GFAP+ cells, is highest in iPSC-Br NSPCs by week 2, with minimal or no astrocytic differentiation observed in iPSC-SC and bona fide NSPCs. (C, G, K) Proliferation rates, assessed by BrdU+ cells, are significantly higher in iPSC-derived NSPCs compared to bona fide NSPCs. (D, H, L) Self-renewal, measured as BrdU+/Sox2+ cells, remains elevated in iPSC-derived NSPCs across both weeks. Each technical replicate consists of 10 standardized images captured from 96-well plates. Data are presented as mean ± s.e.m.; $**p < 0.01$, $***p < 0.001$, $**p < 0.0001$.

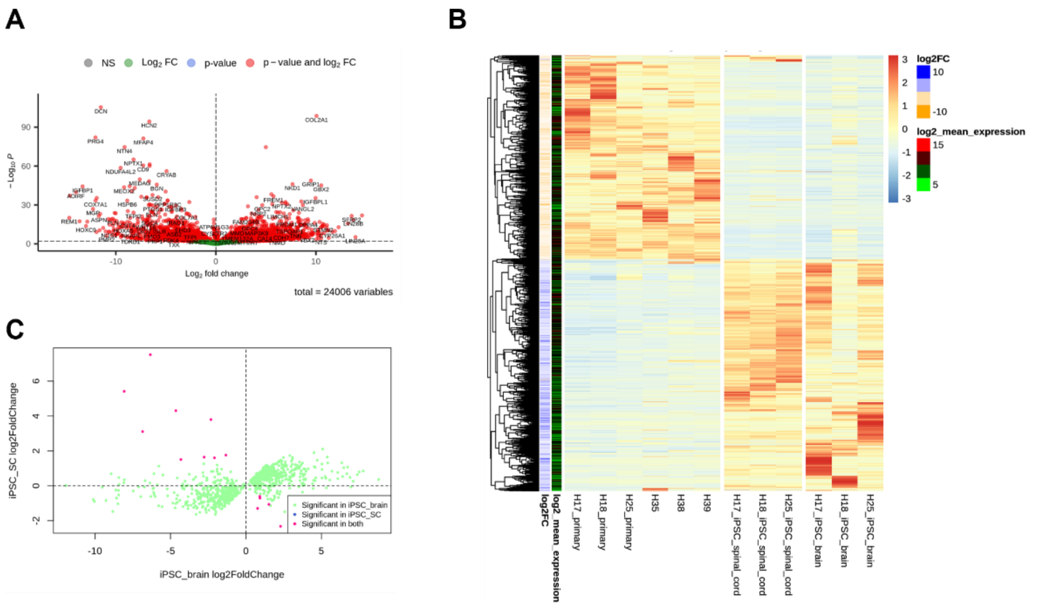

D

Upregulated

NELL2	SYN3
LIN28A	RPRML
LIN28B	UBXN10
SFRP2	TNFRSF8
SLC7A3	CUX2
SYT2	COL2A1
CYP26A1	VRTN
PCDH8	IGFBPL1
RAB3C	FOXB1
KCNK5	TNNT2
TRIM71	RASSF10
BTBD17	FAM181A
MMP9	RHOH
STMN2	AMER2
CAMKV	GRIP1
DSC2	DCX
GDAP1L1	BLACAT1
RNF43	INSM1
GBX2	IL2RB
NTS	ST8SIA3
FBN3	DPPA4
SLC18A3	DCAF12L1
APLNR	ZMAT4
C2CD4C	YBX2
PPP1R17	COL9A1

Downregulated

REM1	C1QTNF7
CST1	CST2
ADIRF	HOXA10
ITIH2	KRBOX1
IGFBP1	CHRM2
HOXC9	ITGBL1
ANGPTL5	NUPR1
MGP	RXFP1
PRG4	SDR42E1
COX7A1	PRELP
HOXB8	OMD
ASPN	CYTIP
FOXD2	OSR2
DCN	NDUFA4L2
FHL5	HOXC6
HTR2A	TDRD9
PMP2	HOXB7
TBX2	DPT
NOS1	ACKR4
EMX2	HOXA5
TSPAN8	MSMP
VSIR	MEOX2
FABP4	IQSEC3
OR51E2	NTN4
PLN	HOXA3

Supplementary Figure S3: iPSC-Br and bona fide NSPCs differentially express 5,555 genes

(A) Volcano plot of genes that are enriched in iPSC-Br (>0) and bona fide (<0) NSPCs. Red dots indicate single DE genes with a fold change ≥2 and a p-value <0.05. A total of 24,006 genes were analyzed. (B) Heatmap and hierarchical clustering of DE genes between iPSC-Br and bona fide NSPCs from (A). The color scale indicates the relative expression: red represents an upregulation, and blue represents a downregulation in iPSC-Br NSPCs. (C) Scatter plot of DE genes exclusively in iPSC-Br NSPCs relative to bona fide NSPCs. 955 genes with a fold change≥2 and a p-value<0.05 are shown. Green dots indicate DE genes only in iPSC-Br NSPCs. (D) The top 50 DE genes upregulated or downregulated in iPSC-Br NSPCs relative to bona fide NSPCs. n=6 bona fide, n=3 iPSC-Br.

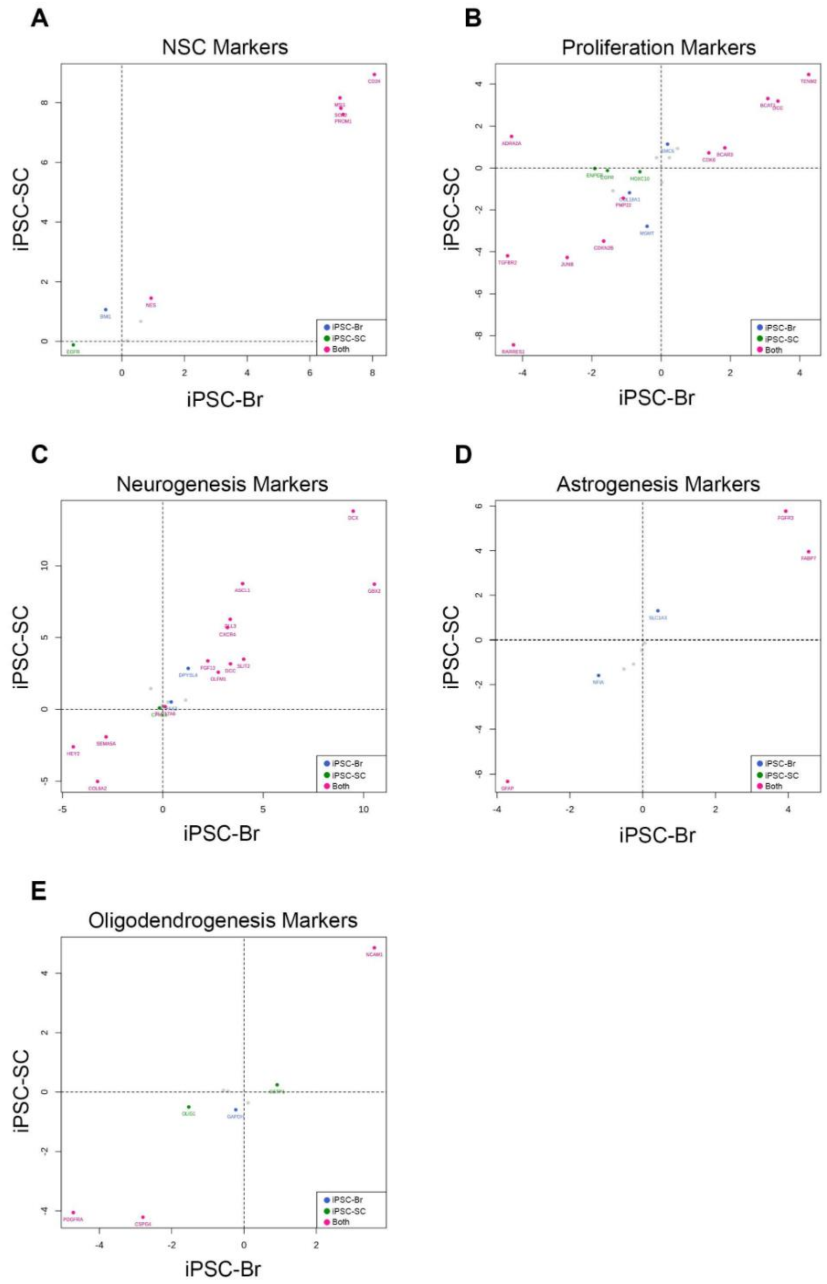

Supplementary Figure S4. Differential Expression of Genetic Markers for NSPCs and Functional Pathways Across Bona Fide, iPSC-SC, and iPSC-Br NSPCs. Scatter plots of differentially expressed (DE) genes unique to iPSC-SC, iPSC-Br, or shared by both iPSC-derived NSPCs relative to bona fide NSPCs. The plots highlight genetic markers for (A) neural stem cells (NSCs), (B) proliferation, (C) neurogenesis, (D) astrogenesis, and (E) oligodendrogenesis. DE genes with a fold change ≥2 or ≤2 and a q-value <0.05 are indicated by colored dots: blue dots represent DE genes exclusive to iPSC-SC NSPCs, green dots to iPSC-Br NSPCs, and pink dots shared by both iPSC-SC and iPSC-Br NSPCs.
